# Beyond absorption: online photoreactor mass spectrometry assessment of new acylphosphine oxide photoinitiators†

**DOI:** 10.1039/d5sc03654b

**Published:** 2025-07-07

**Authors:** Maria Menti-Platten, Brett R. Burns, Oisin J. Shiels, Philip J. Barker, Paul A. Keller, Adam J. Trevitt

**Affiliations:** a Molecular Horizons and School of Science, University of Wollongong NSW 2522 Australia adamt@uow.edu.au

## Abstract

Tools for the rapid and comprehensive characterisation of photoinitiated polymerisation are required to expedite the development of next-generation photoinitiators. For many current workflows, the time consuming synthesis of photoinitiators, in moderate to large scale, impedes progress. This study demonstrates that online photoreactor mass spectrometry facilitates rapid screening of photoinitiated polymerisation on a small scale (*ca.* 1–5 mg). This is demonstrated by photolysis and polymerisation efficiency investigations of nine synthesised monoacylphosphine oxides (MAPOs) compared to a commercial MAPO ((2,4,6-trimethylbenzoyl)diphenylphosphine oxide, (TPO)). All MAPOs undergo photolysis at 395 nm, except for two N(Me)_2_ MAPOs despite their large absorption cross-sections. This highlights how absorptivity alone is an inadequate measure of photoinitiator performance. Additionally, seemingly subtle substitutions and structural differences in the synthesised MAPOs result in drastic changes to the measured polymerisation efficiency. Further analysis attributes this to oxygen inhibition in the initial propagation steps and this demonstrates the advantages of online mass spectrometry to rapidly characterise photoinitiated chemistry.

## Abstract 2

Tools for the rapid and comprehensive characterisation of photoinitiated polymerisation are required to expedite the development of next-generation photoinitiators.

## Graphical abstract

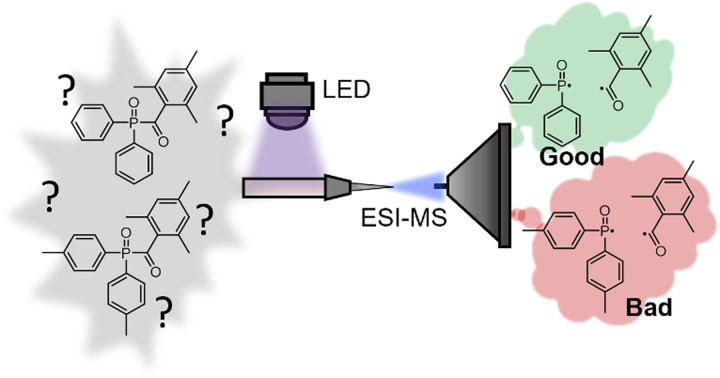

## Introduction

**Scheme 1:**
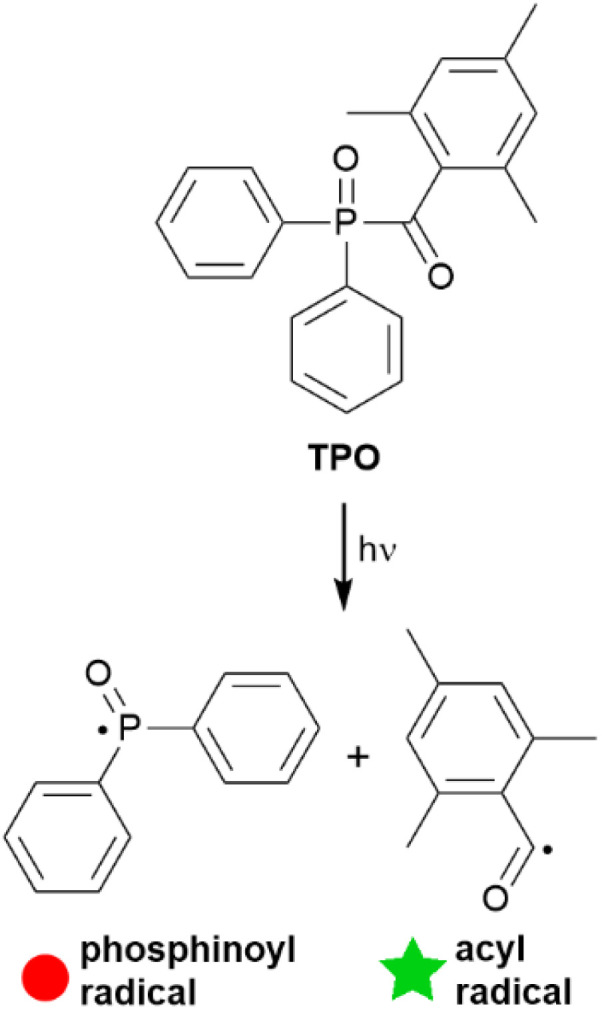
Photolysis of TPO through Norrish type I cleavage to produce the phosphinoyl and acyl radicals. Coloured symbols are assigned for clarity in later discussion.

Photoinitiated radical polymerisation is essential to many applications including coatings,^1^ adhesives,^2^ 3D printing,^3,4^ and biomedical industries.^5,6^ These reactions typically begin with the electronic excitation of a photoinitiator by a photon, and relaxation processes lead to a dissociative state that yields at least one reactive radical capable of polymerisation. For most photoinitiators, radical generation occurs either by bond α-cleavage (Norrish type I) or hydrogen atom abstraction (Norrish type II).^7^ Monoacylphosphine oxides (MAPOs) are a well-established family of photoinitiators, which react *via* typical Norrish type I photodissociation.^8^ Photolysis of MAPOs typically proceeds through a relatively weak S_0_ → S_1_ (nπ*) electronic transition, which is characterised by a low-intensity absorption band at 350–380 nm.^8^ This S_1_ state can relax through intersystem crossing to a dissociative triplet state, where the P–C bond is cleaved to produce a phosphinoyl and acyl radical that can both initiate polymerisation (Scheme 1).^9–15^

The absorption spectra of MAPOs are well matched to the broad emission spectrum of Hg-vapor lamps, however, with the global move toward eliminating Hg-vapour lamps, an alternative irradiation source in this UV-visible range is needed and light emitting diode (LED) technology is the likely replacement. LEDs have several advantages over typical Hg lamps, including no ozone emission, low power needs, low cost, quick turn on, and portability.^16–19^ However, LED emission is narrow and for LED applications, MAPOs that operate efficiently within the narrow band of common LED sources are in demand (*e.g.* bands centered between *λ* = 380 and 420 nm). Reports of modifications to the MAPO scaffold with modified absorption properties and improved polymerisation efficiency are now emerging.^20–26^

While absorption instigates the first step of photoinitiation, the ultimate success of polymerisation depends on the quantum yield of radical cleavage and the subsequent radical reactivity toward monomer addition.^7^ Since accurate theoretical models of photoinitiator performance are yet to be developed, the largest cost associated with tuning the efficiency of new polymerisation systems is the synthesis of new photoinitiators. This often requires many months of laboratory optimisation for the synthesis and purification of practical quantities. Thus, methods that can adequately and rapidly probe photoinitiated chemistry on a small scale are required.

We recently reported a quartz photoreactor (6 cm long tube, 0.5 mm diameter) coupled with an ESI-MS platform that monitors photochemical reactions online. This was benchmarked with the photopolymerisation of methyl methacrylate (MMA) using the industry standard acylphosphine oxide photoinitiators.^27^ The continuous flow reactor arrangement allows for a comprehensive analysis of early-stage intermediates and by-products not otherwise detected with offline techniques. In the current study, this platform is applied to screen a library of newly synthesised MAPO photoinitiators on a small scale (1–5 mg). Relative polymerisation percentages are reported. Termination products, generated from combination and disproportionation reactions are also assigned, as well as unreactive low molecular-weight species that originate from side reactions with molecular oxygen. Ultimately, the characterisation of these intermediates and by-products identifies at which point the photopolymerisation reaction is failing, and aids in the strategic development of future photoinitiators.

## Experimental

MMA and methyl acrylate (MA) (Sigma-Aldrich, 99%) were destabilised by passing through a column of basic alumina and TPO was used as received. Detailed descriptions of synthetic procedures, and compound characterisation data including ^1^H, ^13^C and ^31^P NMR spectra of all photoinitiators are provided in the ESI (S1 and S2).† Complete mass spectra of all polymerisation reactions are also available in Section S3 of the ESI.†

### UV-vis spectroscopy

UV-vis spectra were recorded on a Shimadzu 1800 spectrophotometer at 200–700 nm in a 1 cm quartz cuvette. All photoinitiators were dissolved to a concentration of 5 mM in CH_2_Cl_2_ except for N(Me)_2_ derivatives, which were dissolved to a concentration of 0.05 mM in CH_2_Cl_2_ due to the drastic increase in extinction coefficients. Extinction coefficients were determined using single point measurements of absorption relative to a solvent blank and assumed to follow Beer's Law.

### Polymerisation with online photoreactor ESI-MS

All polymerisations were performed using our previously described online photoreactor coupled with a commercial ESI-MS linear quadrupole ion trap (LTQ) platform,^27^ shown in the image of Fig. 2. Briefly, an LED (395 nm, max power 22 μW mm^−2^, with T-Cube LED Driver, Thorlabs), positioned orthogonally to a 6 cm quartz tube (0.5 mm inner diameter, 11.8 μL volume), irradiates the reaction solution, initiating polymerisation prior to electrospray ionisation. This quartz reactor is connected in-line to a dual tip electrospinning needle as the ESI spray source, which is connected to a high voltage power supply (maintained at +3 kV), charging the needle to generate a Taylor cone required for ESI. Ions are guided into the ion trap where they can be mass-isolated, stored, and (if required) fragmented using collision-induced dissociation (CID) for further characterisation. Polymerisation reactions consisted of a solution of MAPO (5 mM) in MMA or MA (1 mL) which was introduced to the reactor at a flow rate of 20 μL min^−1^ (*ca.* 2 mm s^−1^ flow velocity, *ca.* 30 s residence time). HPLC grade MeOH was used as the ESI solvent, which was also introduced at a flow rate of 20 μL min^−1^ (total 40 μL min^−1^ flow rate). Since both the photoreactor solution and ESI solvent mix at the spray tip, unwanted reactions with methanol in the quartz cell are avoided. The presence of oxygen in the flow reactor is due to ambiently dissolved O_2_ gas in MMA, and the reported [O_2_] in MMA is approximately 10^−3^ M.^28^

From the mass spectrum, the percentage of polymerisation for each reaction was calculated by dividing the integrated intensity of all propagating oligomers by the intensity of all photoproducts. An example of this calculation for TPO is shown in eqn (1). The percentage of polymerisation greater than *n* = 2 and *n* = 3 was calculated by dividing the intensity of all *n* > 2 or *n* > 3 oligomers by the intensity of all photoproducts. In these calculations the ionisation efficiency is assumed to be the same for all species. This assumption will likely hold for small oligomers but may be a less reliable approximation when oligomers increase in length and allow multiple protonation site isomers.1
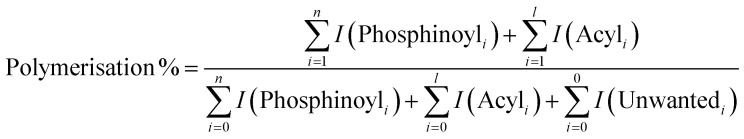
where *I*(*x*) is a function that returns the intensity of the peak *x* in the spectrum and Phosphinoyl_*i*_ or Acyl_*i*_ represents the *m*/*z* of the *i*-th product of species series Phosphinoyl or Acyl. For example, Phosphinoyl_4_ corresponds to the *n* = 4 oligomer of the propagating phosphinoyl radical, *i.e. m*/*z* 601.

## Results and discussion

### Synthesis

**Scheme 2:**
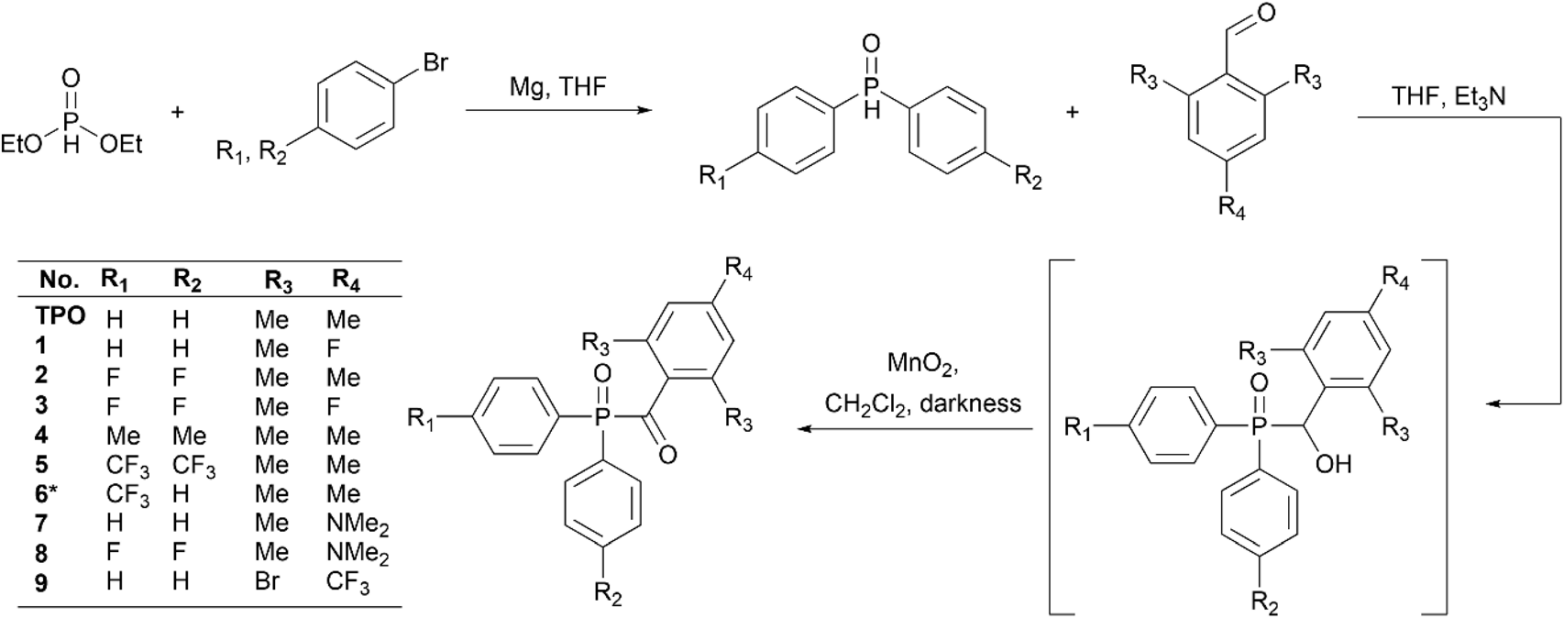
Synthesis of the MAPOs in this study, with the substitution pattern of each compound in this study tabulated. *Ethyl phenyl phosphinate was used as the electrophile in the Grignard reaction to synthesise MAPO 5.

This study examines a series of synthesised MAPO derivatives as practical photoinitiators using a photoreactor coupled mass spectrometer.^27^ These derivatives were designed and synthesised based on the MAPO scaffold with varying electron withdrawing and donating substitutions, which are hypothesised to have an effect on both photolysis and polymerisation reactivity. Substitution at the *ortho* position provides steric bulk and prevents solvolytic cleavage,^22,29^ and thus CH_3_ groups are maintained at these *ortho* positions on the acyl ring. The exception to this is MAPO 9, where bromo groups are introduced to the *ortho* position. In this case, the bromo substituents were intended to be replaced with CH_3_, however, to date this synthesis has been unsuccessful. Modifications to the *para* position follow previous studies that demonstrate significant and intricate effects on photoinitiator performance but nevertheless were rationalised.^7^ The synthesis of MAPOs was optimised (Scheme 2),^30,31^ and the complete synthetic protocols, building block synthesis and characterisation are provided in Section S1 of the ESI.† A Grignard reaction between the *p*-substituted aryl bromide and the phosphite electrophile produced *p*-substituted diphenylphosphine oxides (DPPOs). In the case of the asymmetric DPPO required to produce MAPO 6, ethyl phenylphosphinate was used as the electrophile. The Abramov addition of the *p*-substituted DPPO to a 2,4,6-trisubstituted benzaldehyde produced the α-hydroxyphosphine oxide intermediate. Initially, the α-hydroxy intermediates were purified and isolated. However, those bearing a CF_3_ group on the phosphinoyl moiety were susceptible to a retro-Abramov reaction upon attempted purification by column chromatography, which resulted in the recovery of starting materials. Furthermore, the synthesis of α-hydroxyl compounds with a N(Me)_2_ substituted acyl ring produced in an inseparable mixture when analysed by thin layer chromatography (TLC). Therefore, the α-hydroxyphosphine oxide precursors for MAPOs 5–9 were not isolated, and once complete consumption of the corresponding DPPO and substituted benzaldehyde starting materials was observed by TLC analysis, the reaction mixture was evaporated to dryness and immediately subjected to oxidation with MnO_2_ in CH_2_Cl_2_. Filtration of the reaction through Celite and purification by silica gel column chromatography afforded the desired MAPOs.

**Fig. 1:**
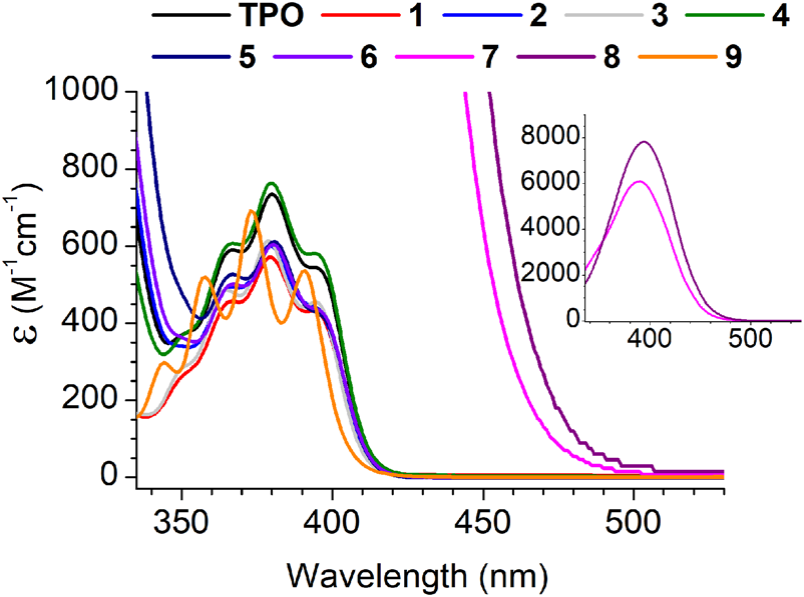
Absorption spectra of all MAPOs in this study, with CH_2_Cl_2_ as the solvent. The inset is plotted for MAPOs 7 and 8 for an almost an order of magnitude larger vertical scale.

Absorption spectra for the MAPOs in this study are presented in Fig. 1. At a photon wavelength of 395 nm, the MAPOs without N(Me)_2_ substitution (1–6 and 9) displayed comparable extinction coefficients (*ca.* 500 M^−1^ cm^−1^) to that of TPO (545 M^−1^ cm^−1^). Additionally, the internal features of the absorption spectrum of TPO are generally retained for these derivatives. However, both N(Me)_2_ derivatives 7 and 8 showed a dramatic increase in extinction coefficient of an order of magnitude greater than TPO, at 5989 and 7829 M^−1^ cm^−1^ (395 nm), respectively. Both MAPOs 7 and 8 exhibit a major change in absorption bandshape, which presents as a large, broad single peak.

This is consistent with previously reported dialkylamino-MAPOs^32,33^ and in accord with the dimethylamino-benzoin system, which is reported to also exhibit a strong and broad band absorption peaks centred at 340 nm (FWHM = *ca.* 40 nm).^7^ The photopolymerisation results below support that absorptivity cannot be directly correlated to reactivity, and a drastic increase in absorptivity can have detrimental effects on polymerisation.

### Photoinitiated polymerisation results

**Fig. 2:**
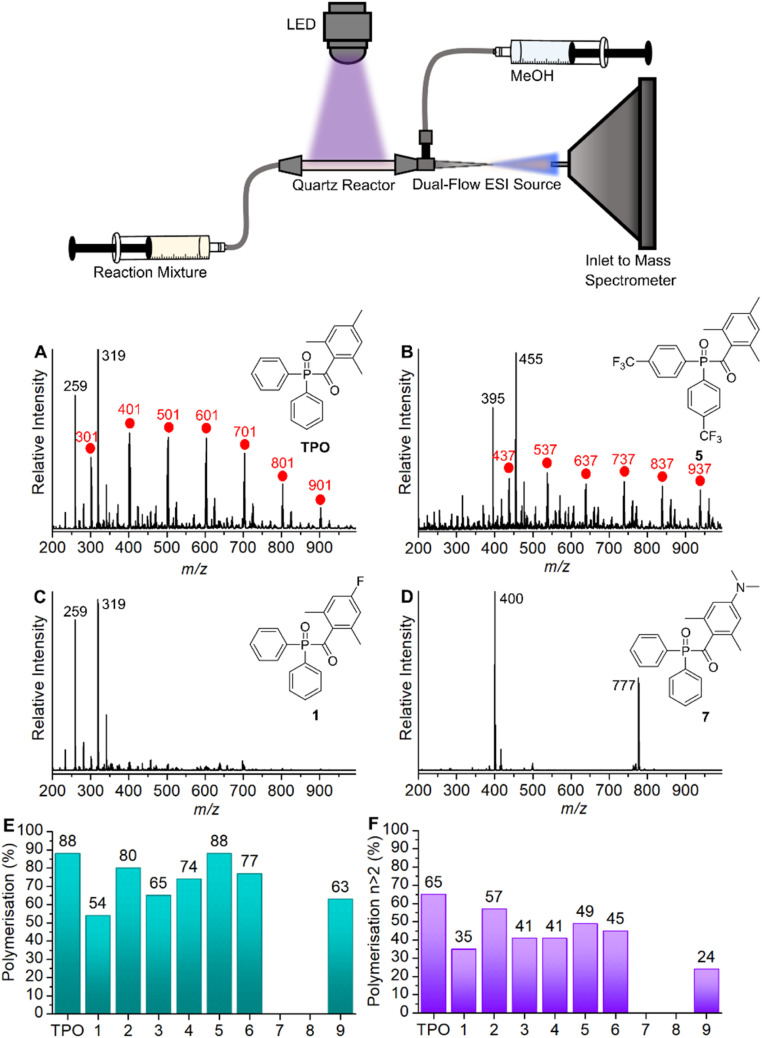
(Top) Schematic of the quartz cell photochemical reactor setup. Representative mass spectra from the reaction of MMA with (A) TPO. (B) MAPO 5. (C) MAPO 1. (D) MAPO 7. (E) Polymerisation percentages of MMA with each MAPO in this study. (F) Polymerisation percentages of MMA with each MAPO in this study, where *n* > 2.

Mass spectra of the photoinitiated polymerisations of MMA (100 Da) with TPO, 1, 5, and 7 are shown in Fig. 2A–D (see Section S3† for all mass spectra). To summarise the degree of MMA polymerisation with each MAPO, Fig. 2E and F shows the value of the polymerisation % derived from the respective mass spectrum, using eqn (1). The reaction of TPO with MMA was performed in triplicates as an indicator of reproducibility, and the results are presented in Table S1 (see ESI).† The representative mass spectrum for the polymerisation of MMA with TPO as the initiator is shown in Fig. 2A. The mass spectrum displays peaks spaced by 100 Da, consistent with previously reported ESI-MS results of MMA polymerisation.^34^

The peaks spaced by 100 Da from *m*/*z* 301 indicate successive additions of MMA to the polymer chain initiated by the phosphinoyl radical (labelled with red circles). A more detailed discussion of the identifiable photoproducts is provided below; however, these mass spectra provide a rapid, relative and semi-quantitative assessment of the photopolymerisation performance of each chemical system. The mass spectrum of trifluoromethylated MAPO 5, shown in Fig. 2B, is similar to the TPO case, where the progression of polymer chain growth starts with a peak at *m*/*z* 437, with repeat units every +100 Da. This again corresponds to radical propagation after initiation by the phosphinoyl radical (red circles). The *m*/*z* peaks are shifted by 136 Da, caused by the substitution of two CF_3_ groups (136 Da) to the phenyl rings. Furthermore, the mass spectra for polymerisation with MAPOs, where the mesitoyl ring was retained and the phosphinoyl moiety was substituted with either F (2) or CF_3_ (5, 6), were comparable to that of TPO. In each case, the *m*/*z* peaks shift by the respective changes to phosphinoyl substitution (*i.e.*, 36 Da shift for F substitution), which indicates that the phosphinoyl moiety is preserved in these systems. While the polymerisation is mostly successful in Fig. 2A and B, the mass spectra also display low mass peaks (*m*/*z* 200–350), which appear unreactive toward further monomer addition.

MMA polymerisation with MAPO 1 is shown in Fig. 2C. Under the same conditions, this reaction appears mostly unsuccessful with low mass peaks (*m*/*z* 200–350) that dominate the spectrum, and no obvious progressions from these peaks. The same low reactivity pattern was also observed in MAPOs 3, 4, and 9, which all varied in phenyl and acyl substitution. For TPO, and MAPOs 1–6 and 9, upon LED irradiation the *m*/*z* signal of the parent photoinitiator was absent and it is presumed that under these conditions the photoinitiators underwent complete photolysis. The mass spectrum obtained from the reaction with N(Me)_2_ MAPO, 7, shows peaks corresponding to the unreactive parent MAPO (Fig. 2D) and no evidence of propagating radicals. The same trend occurs with the second N(Me)_2_ MAPO 8, where photolysis products were not evident, and therefore polymerisation was not initiated. To further confirm the photostability of MAPO 7 and 8 under the experimental conditions, the change in ion count of the parent molecule was monitored relative to the total ion count before and during irradiation. The results are presented in Tables S2 and S3 (ESI),† which support the absence of photolysis in both derivatives.

As mentioned above, to quantify and compare the success of the photoinitiator, the polymerisation % was calculated by integrating each of the assigned polymer peaks and normalising this to the integrated signal of both unreactive species and polymer peaks. This serves as a useful relative assessment of the effectiveness of the photoinitiator under these conditions. For TPO, an 88% conversion to polymeric material was calculated. Substitution to the *para* position of both phenyl rings with CF_3_ (MAPO 5) had no apparent effect on degree of polymerisation at 88%, however, *para*-CF_3_ substitution of only one phenyl ring resulted in a decrease of polymerisation to 77% in MAPO 6. Substituting the *para* position of both phenyl rings with fluorine resulted in a slight decrease to the overall polymerisation to 80% (MAPO 2). Furthermore, the substitution of fluorine for methyl groups resulted in a further decrease to 74% (MAPO 4). Although fluorinating the phenyl rings had a negligible effect on polymerisation, the introduction of fluorine to the acyl ring significantly reduced polymerisation in both MAPOs 1 and 3 with results of 54% and 65%, respectively. In the case of MAPO 9, where the acyl ring was substituted with Br and CF_3_, 63% polymerisation and significant decomposition of the acyl ring were observed.

For both N(Me)_2_ MAPOs 7 and 8, only *m*/*z* signals corresponding to the parent MAPOs were detected and polymerisation was largely unsuccessful. Previous steady state photolysis experiments indicate that the photolysis of MAPO 7 is inefficient,^32^ in accord with the finding reported here. As reported by Le *et al.*, where the NMe_2_ group of MAPO 7 was further methylated, the resulting quaternary ammonium derivative underwent efficient photolysis.^32^ This suggests that the lone pair on the nitrogen atom of NMe_2_ derivatives contributes to competing photochemical pathways by extending the π-system of the benzoyl moiety.

Fig. 2F shows the percentage of *n* > 2 polymerisation in each mass spectrum, that is, the fraction of polymerisation that has undergone at least two monomer additions, and this provides a relative quantitative means to assess and compare photoinitiator performance. Overall, these results generally conform with the same trends as Fig. 2E, with the highest polymerisation yields for TPO, MAPOs 2, and 5, and lowest polymerisation yields for MAPOs 1 and 9. As mentioned above, MAPOs 7 and 8 are non-active as photoinitiators under these conditions, despite their *ε* values being an order of magnitude greater than the other MAPOs in this study. As demonstrated, minor structural modifications to the TPO scaffold induce significant and sometimes major alterations in the overall polymerisation efficiency. Since both electron donating and withdrawing groups contribute to a decrease in overall polymerisation when compared with TPO, these observations cannot be simply attributed to such properties and nor is absorptivity a strong indicator for photoinitiator effectiveness. In fact, absorption of a photon is only the first of many steps in a photochemical process, and there is a fundamental mismatch between absorptivity and reactivity, supported by recent advances in action spectroscopy.^35–39^

**Fig. 3:**
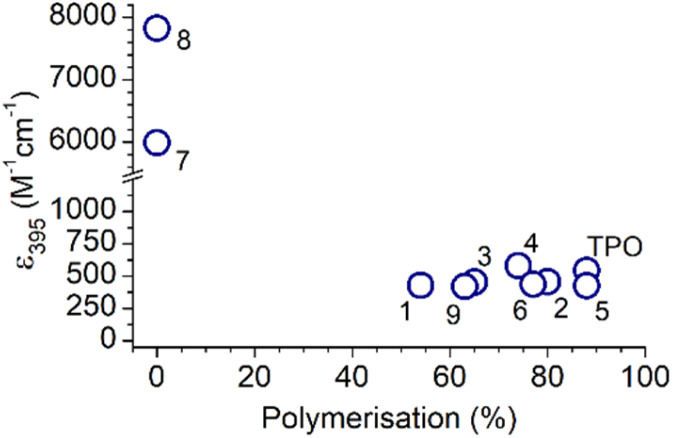
A plot of the molar extinction coefficient against the overall polymerisation percentage of all compounds in this study.

To assess the differences in absorptivity and the effect on reaction outcomes, the absorption coefficients were plotted against the percentage of polymerisation (Fig. 3). No trend is apparent and, in fact, the N(Me)_2_ MAPOs (7 and 8) with the greatest absorptivity were inactive as photoinitiators. For the N(Me)_2_ substituted MAPOs 7 and 8, the significantly higher absorption cross section and poor photoinitiation performance is consistent with the literature similar dimethylamino benzoin-type species,^7^ where quantum chemical calculations and photochemical lifetime measurements explain this observation by the presence of a strongly absorbing ππ* singlet state that, however, is incompatible with intersystem crossing to appropriate dissociative triplet states. Despite the similarities in the absorbance spectra of MAPOs 1–6 and 9 to that of TPO, each derivative displayed significant differences in photoinitiator performance. This further supports a growing body of evidence that suggests the photoinitiation efficiency of a molecule does not simply correlate to absorptivity.^7,36,37,39–41^ Further analysis of the photoreactor mass spectra of these photoinitiation systems provides insight into the origin of incomplete polymerisation.

**Fig. 4:**
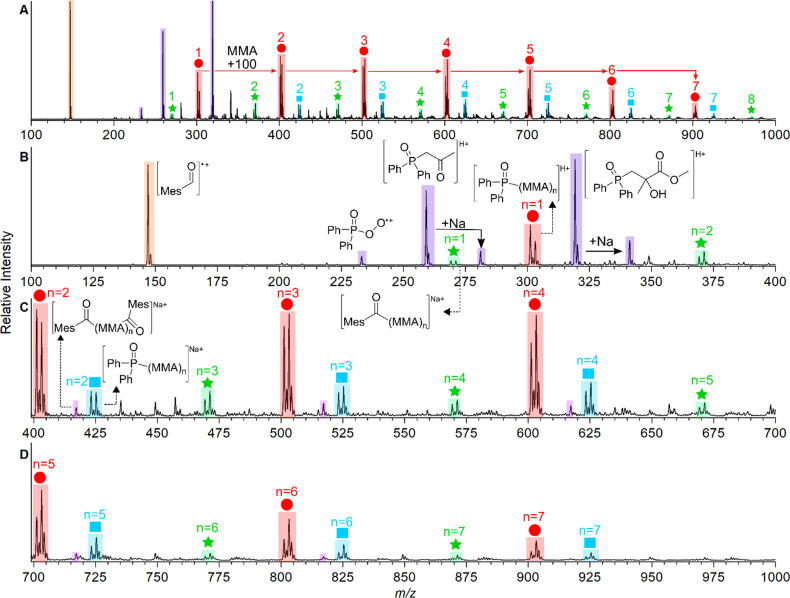
(A) Mass spectrum of the photopolymerisation of TPO with MMA with irradiation at LED395 from *m*/*z* 100–1000, (B) *m*/*z* 100–400, (C) *m*/*z* 400–700, (D) *m*/*z* 700–1000.

A detailed analysis of MMA polymerisation with model compound TPO, outlined in Fig. 4, includes the structural assignment of key adducts. An ESI mass spectrum at *m*/*z* 100–1000 is provided in Fig. 4A, with repeat units denoted by either red circles, blue squares or green stars and the number of MMA additions noted above the symbol (see Scheme 1 for radical structures). Termination products by disproportionation appear as pairs of peaks with a spacing of 2 Da, where termination of the chain results in either an alkene or alkane depending on the role of the end-group in H-abstraction.

An expanded mass spectrum between *m*/*z* 100–400 provided in Fig. 4B shows a peak at *m*/*z* 148, assigned to the mesitoyl (Mes) radical as a radical cation (orange highlight). MMA addition to the propagating acyl radical forms the sodiated (Na^+^) disproportionation species at *m*/*z* 269 and 271 (*n* = 1), which repeat every 100 Da corresponding to successive MMA addition (green highlight/stars). A peak at *m*/*z* 417 (Fig. 4C, pink highlight) corresponds to a sodiated combination product, where the growing polymer that originates from a mesitoyl radical is capped by another mesitoyl radical.

Polymerisation of the phosphinoyl radical is observed from the *n* = 1 ion at *m*/*z* 301 and 303 (M + H^+^) as alkene and alkane disproportionation repeating units (red highlight/circles). Although secondary cleavage is less favourable, the loss of a phenyl ring from the phosphinoyl species could give rise to a biradical. This is observed from *n* = 2 as a pair of disproportionation products at *m*/*z* 423 and 425, however, it is also plausible these peaks are the sodiated adducts of MMA addition to the mono-radical (M + Na^+^, blue highlight/squares). The prominence of the unreacted mesitoyl radical, along with complete consumption of the phosphinoyl radical, implies that acyl radicals are less reactive towards monomer addition than their phosphinoyl counterpart.^42,43^ Expansion of the spectrum between *m*/*z* 700–1000 in Fig. 4D shows consistent addition of MMA to the identified propagating radicals.

Along with the expected propagation products, unreactive non-repeat product peaks were identified. Given the established reactivity of phosphinoyl radicals toward molecular oxygen, the peaks at *m*/*z* 233, 259 and 319 along with their sodiated adducts (Fig. 4B, purple highlight), are thought to originate from O_2_ reactions. It is well known that O_2_ inhibition is a major challenge that thwarts free radical polymerisation and reduces the yield of polymerisation.^1,44^ As such, it is highly likely that these subtle structural modifications have changed the affinity of these propagating radicals towards O_2_. To further verify these assignments, additional structural characterisation experiments were conducted.

### Structural elucidation of unreactive species

**Fig. 5:**
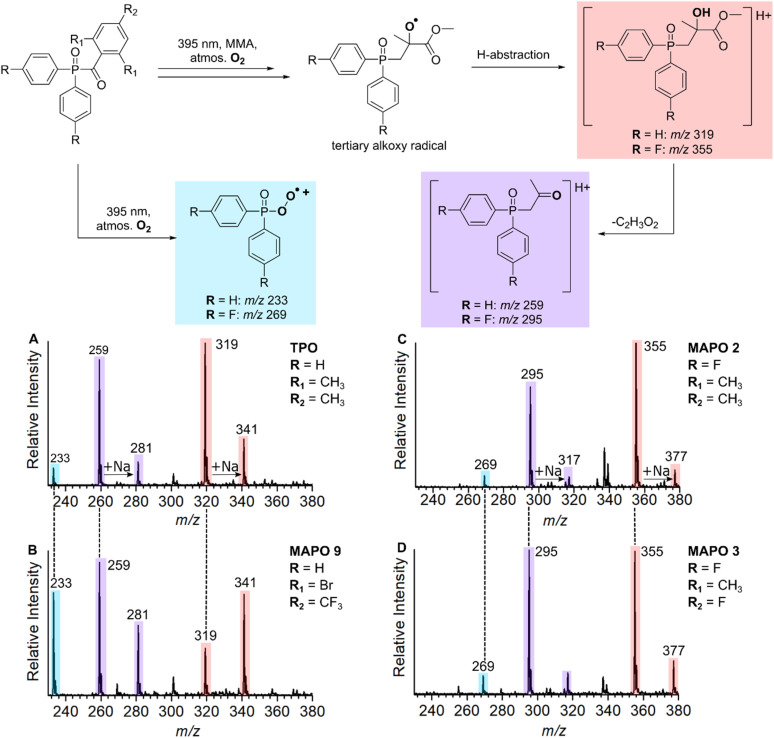
Scheme (Top) – the proposed oxygenated byproducts generated from the reaction of MAPOs with MMA at 395 nm. (Bottom) – Mass spectra at *m*/*z* 320–380 of the reaction of MMA with (A) TPO, (B) MAPO 9, (C) MAPO 2, and (D) MAPO 3.

Although the reactivity of phosphinoyl radicals toward O_2_ has been reported previously,^9,15,45^ structural elucidation of these key oxygenated products has not been investigated. This section characterises these unreactive species with structures that are consistent with the O_2_ quenching of propagating radicals. The assignment of products that originate from O_2_ inhibition is summarised in the reaction scheme in Fig. 5, where it is hypothesised that O_2_ reacts with the *n* = 0 and *n* = 1 adducts to produce unreactive byproducts. Fig. 5A–D shows mass spectra of MMA polymerisation with TPO, and MAPOs 9, 2, and 3 at *m*/*z* 230–380. The photoinitiated reaction of MMA with TPO results in three low molecular weight species at *m*/*z* 233, 259, and 319 as shown in Fig. 5A. The unreactive species of MAPO 9 in Fig. 5B exhibit identical *m*/*z* peaks to TPO (*m*/*z* 233, 259, and 319), despite substitution of the acyl ring with Br and CF_3_ groups. This, along with the absence of Br isotopic patterns suggests that the entire acyl ring is lost, and the *m*/*z* shift of these low molecular weight products may be dependent on phosphinoyl substitution. This hypothesis is supported by the analysis of MAPO 2 in Fig. 5C, where in comparison to TPO, the mesitoyl ring is retained and the phenyl rings of the phosphinoyl moiety are substituted with fluorine. Compared with TPO and 9, the reaction of MMA with MAPO 2 shifts the unreactive byproducts by 36 Da, which produces peaks at *m*/*z* 269, 295, and 355. This corresponds to the substitution of two fluorine atoms, which is concordant with the substitution pattern of the phosphinoyl moiety. Compared to the mesitoyl ring of 2, the acyl ring of 3 is substituted with fluorine in the *para*-position, while both 2 and 3 contain the same fluorinated phosphinoyl moiety. As shown in Fig. 5D, the reaction of MAPO 3 with MMA results in oxygenated species with the same *m*/*z* as those in Fig. 5C. This supports that the entire phosphinoyl moiety is retained, and that the acyl ring does not contribute to the structure of the unreactive byproducts.

**Fig. 6:**
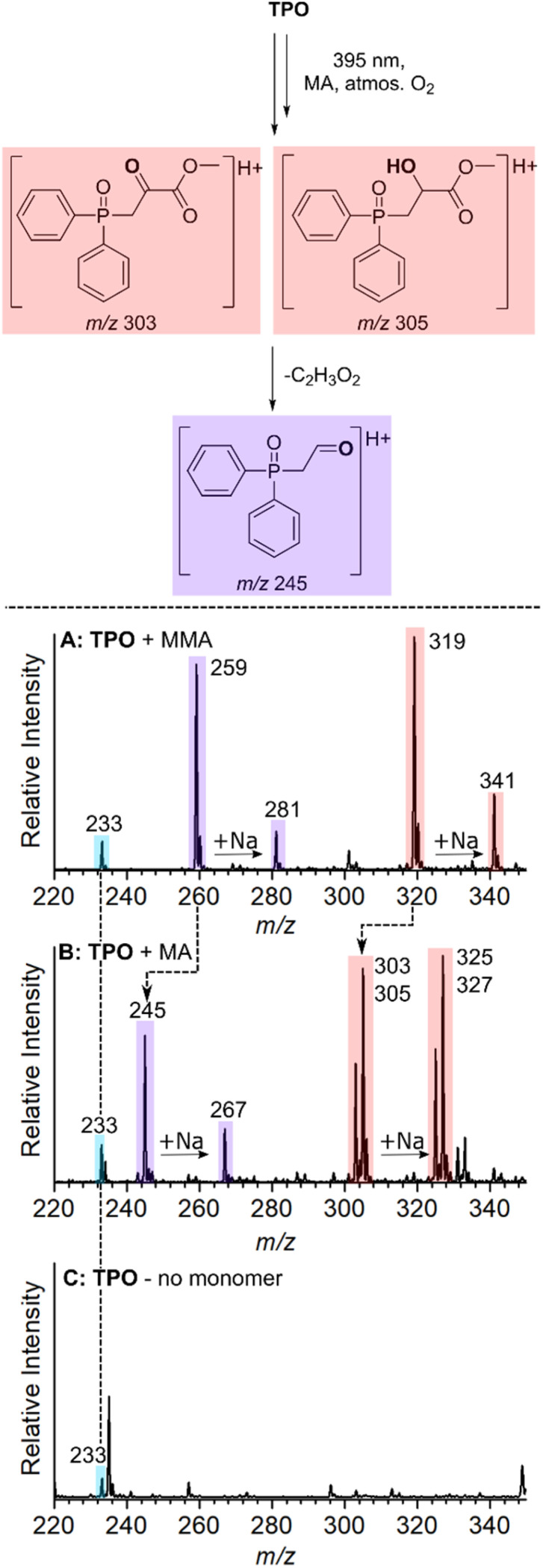
Scheme (Top) – the proposed oxygenated byproducts generated from the reaction of MAPOs with MA at 395 nm. (Bottom) – Mass spectra at *m*/*z* 220–340 of the reaction of TPO with (A) MMA, (B) MA, and (C) no monomer (MeOH was used as the solvent where no monomer was used).

It is proposed that the unreactive products originate from the reaction of O_2_ with either the phosphinoyl radical or the *n* = 1 radical, where one monomer unit has added to the phosphinoyl radical. The proposed O_2_ inhibition products are shown in the scheme of Fig. 5 and are highlighted in blue, red, and purple. Addition of O_2_ directly to the phosphinoyl radical produces the peroxyphosphorus radical, which in the case of TPO, gives rise to the radical cation at *m*/*z* 233 highlighted in blue. It is proposed that the products highlighted in red and purple originate from initial addition of O_2_ to the *n* = 1 propagating alkyl radical to generate a peroxyl radical. This peroxyl radical can undergo bimolecular termination to produce the tertiary alkoxy radical shown in the scheme of Fig. 5. Termination of the alkoxy radical occurs by hydrogen abstraction to produce the alcohol highlighted in red, and subsequent α-cleavage of the methyl ester produces the ketone highlighted in purple. For TPO, hydrogen abstraction gives rise to the peaks at *m*/*z* 319 (M + H^+^) and *m*/*z* 341 (M + Na^+^), and subsequent loss of C_2_H_3_O_2_ results in the peaks at *m*/*z* 259 (M + H^+^) and *m*/*z* 281 (M + Na^+^) highlighted in Fig. 5A. The CID activation of *m*/*z* 319 produced a fragment at *m*/*z* 259 (see ESI 3.2†). Further CID of this *m*/*z* 259 peak produced a peak at *m*/*z* 201, which is assigned to the phosphinoyl radical (M + H^+^). The CID of *m*/*z* 259 generated from both the reaction and from the CID of *m*/*z* 319 have identical fragmentation patterns, which supports that, in the reaction, the peak at *m*/*z* 259 is formed from the parent ion at *m*/*z* 319. We hypothesise that the byproducts discussed above are a result of O_2_ addition either to the phosphinoyl radical or to the *n* = 1 adduct. To probe the mechanism further, while retaining TPO as the photoinitiator, three experiments were carried out using MMA, MA, and a control sample containing TPO without monomer. The objective here was to see whether the *m*/*z* 259 and *m*/*z* 319 peaks were shifted due to a change in monomer. The results are presented in Fig. 6A–C and the proposed products of O_2_ inhibition are shown in the scheme above the mass spectra. Inspection of Fig. 6 reveals that the peak at *m*/*z* 233 is retained whether TPO reacts with MMA (Fig. 6A), MA (Fig. 6B) or indeed, in the absence of monomer (Fig. 6C). This strongly suggests that direct addition of O_2_ to the parent phosphinoyl radical of Scheme 1 occurs to yield the same phosphorusperoxyl radical (*m*/*z* 233), highlighted blue in Fig. 5, in each case. The peaks at *m*/*z* 259 and *m*/*z* 319 that are present in Fig. 6A shift by 14 Da when MA is used as the monomer (Fig. 6B). Further, as shown in Fig. 6C, these peaks are absent where TPO is reacted without the presence of monomer, confirming that the formation of *m*/*z* 259 and *m*/*z* 319 relies on the addition of one monomer unit followed by O_2_ inhibition. The pathway for O_2_ inhibition where MA is the monomer is proposed to be similar to that described above where the addition of O_2_ to the *n* = 1 propagating radical produces a peroxyl radical. However, unlike MMA, termination of the peroxyl radical would not produce a tertiary alkoxy radical. Instead, in accord with the work of Coote and co-workers,^46,47^ bimolecular termination of peroxyl radicals originating from secondary alkyl radicals, such as those from MA, produces both a ketone and an alcohol. Both products are shown in the scheme of Fig. 6, highlighted in red. The ketone gives rise to peaks at *m*/*z* 303 (M + H^+^) and *m*/*z* 325 (M + Na^+^), while the alcohol results in peaks at *m*/*z* 305 (M + H^+^) and *m*/*z* 327 (M + Na^+^), shown in Fig. 6B. α-Cleavage of the methyl ester (–C_2_H_3_O_2_) is also observed in the reaction with MA, resulting in the aldehyde product that is highlighted purple and assigned to the peak at *m*/*z* 245 (M + H^+^) and *m*/*z* 267 (M + Na^+^) in Fig. 6B. Curiously, oxygenated products were absent from the *n* = 2 propagating radical onwards, which indicates that O_2_ inhibition is less favourable for larger alkyl chains. No apparent trend can be deduced by simple comparison of the molecular substitution, and it remains unclear why the same phosphinoyl radical generated from each MAPO with a different acyl substitution, changes the susceptibility of O_2_ inhibition to the phosphinoyl radical. However, the degree of O_2_ inhibition ranges from 12–46% in these systems, which significantly impedes the polymerisation reaction. In this study, the fundamental issue of the photoinitiated polymerisation of MAPO systems is clearly displayed in the mass spectra and, ultimately, it is O_2_ inhibition at the phosphinoyl and *n* = 1 propagating radicals that is the root cause of incomplete photoinitiated polymerisation.

## Conclusions

A robust theoretical model for the performance of MAPOs is yet to be developed, and the inability to accurately predict the effect of substitution on photoinitiator efficiency remains a challenge in designing photoinitiators. Therefore, experimental methods that can rapidly and comprehensively screen newly synthesised photoinitiators on a small reaction scale are required. In this study, nine MAPO derivatives were synthesised with substitution of electron withdrawing (F, CF_3_) and electron donating (NMe_2_, Me) groups to the acyl and phosphinoyl rings. The mild and optimised synthetic route would be suitable for the upscale of promising candidates. This study demonstrates the applicability of an online photoreactor coupled ESI-MS to rapidly screen these synthesised MAPOs with only 1–5 mg of compound. Both photolysis and polymerisation of each MAPO were monitored and benchmarked against TPO, and the results are summarised in Table 1. Although all the synthesised derivatives were less effective than TPO, this method identified at which point each reaction failed. For NMe_2_ substituted compounds, 7 and 8, which displayed the greatest absorptivity, the limiting step is photolysis. This demonstrates that absorptivity alone is an inadequate indicator of photoinitiation efficiency. Instead, photochemical action plots should be considered to map the wavelength-resolved photochemical reactivity of these MAPOs, which is part of ongoing work in our laboratory. For TPO, and MAPOs 1–6 and 9 complete photolysis was observed, indicating that the substitution with F, CF_3_ and Me, at either the chromophore or the phosphinoyl moiety did not negatively affect photodissociation. However, for each photoinitiator, the polymerisation reaction was impeded by the formation of three unreactive species, which for TPO were present at *m*/*z* 233, 259 and 319. Analysis of the mass spectrum of TPO polymerisation identified the peak at *m*/*z* 233 as direct O_2_ addition to the phosphinoyl radical (*n* = 0). The peak at *m*/*z* 319 was assigned to O_2_ addition followed by alkoxyl termination at the first monomer addition (*n* = 1) and ensuing methyl ester loss resulted in the peak at *m*/*z* 259. Oxygen inhibition remained unchanged even when MMA was substituted for MA, highlighting that this process is independent of the monomer. The extent of oxygen inhibition between MAPO derivatives could not be explained by modifications to either the acyl or phosphinoyl moiety. However, it is evident that O_2_ inhibition is most crucial in the first two steps of polymer initiation, that is, radical formation and the first monomer addition. Therefore, future work on understanding how O_2_ interacts with the photoinitiator would be beneficial in developing oxygen-resistant photoinitiators. For example, non-covalent interactions between O_2_ and the MAPO prior to photolysis could explain why seemingly minor structural changes to the molecular scaffold impart drastic differences in O_2_ inhibition.

**Table 1:** Properties and polymerisation results of the nine synthesised MAPOs compared with TPO

MAPO	*λ*_max_ (nm)	*ε*_395_ (M^−1^ cm^−1^)	Photolysis	Polymerisation (%)	Polymerisation *n* > 2 (%)
TPO	380	545	✓	88	65
1	379	430	✓	54	35
2	379	458	✓	80	57
3	379	454	✓	65	41
4	379	580	✓	74	41
5	381	427	✓	88	49
6	380	436	✓	77	45
7	390	5989	✗	0	0
8	395	7829	✗	0	0
9	373	421	✓	63	24

## Author contributions

MMP: data curation, methodology, investigation, formal analysis, methodology, writing – original draft, writing – review and editing. BRB: data curation, methodology, formal analysis, writing – review and editing. OJS: data curation, methodology, formal analysis, writing – review and editing. PJB: conceptualisation, funding acquisition, supervision, writing – review and editing. PAK: conceptualisation, funding acquisition, supervision, writing – review and editing. AJT: conceptualisation, funding acquisition, supervision, writing – review and editing.

## Conflicts of interest

There are no conflicts to declare.

## Supplementary Material

SC-016-D5SC03654B-s001

## Data Availability

Detailed procedures, characterisation, and NMR spectra of intermediates and final products along with mass spectra of MAPOs and CID spectra are provided in the ESI.†
