# Supplementary material for: Beyond absorption: online photoreactor mass spectrometry assessment of new acylphosphine oxide photoinitiators
Source: Chem Sci. 2025 Jul 7;16(32):14625–34. doi: 10.1039/d5sc03654b (PMC12257168; doi:10.1039/d5sc03654b)
Supplement: SC-016-D5SC03654B-s001 [file SC-016-D5SC03654B-s001.pdf]

Supplementary Information (SI) for Chemical Science.
This journal is © The Royal Society of Chemistry 2025

Electronic Supporting Information for:

Beyond Absorption: Online Photoreactor Mass Spectrometry Assessment of New Acylphosphine Oxide Photoinitiators

Maria Menti-Platten,[a] Brett R. Burns,[a] Oisin J. Shiels,[a] Philip J. Barker,*[a] Paul A. Keller,*[a] and Adam J. Trevitt*[a]

[a]Molecular Horizons and School of Science, University of Wollongong, Australia, 2522

E-mail: adamt@uow.edu.au, keller@uow.edu.au, pbarker@uow.edu.au

Phone: +61 2 4221 5545, +61 2 4221 4692

Contents

ESI 1 Experimental Details 2

ESI 1.1 Photoreactor Mass Spectrometry 2

ESI 1.2 General Chemistry 3

ESI 1.3 General Synthesis 5

ESI 1.4 Building Block Synthesis 5

Synthesis of secondary phosphine oxides 5

Synthesis of substituted benzaldehydes 8

ESI 1.5 General procedure A: Synthesis of α-hydroxyphosphine oxides 11

ESI 1.6 General procedure B: Oxidation of α-hydroxyphosphine oxides to synthesise monoacylphosphine oxides 11

ESI 1.7 General procedure C: Two-step synthesis of monoacylphosphine oxides 11

ESI 2 NMR Characterisation 20

ESI 2.1 NMR spectra of secondary phosphine oxides 20

ESI 2.2 NMR Spectra of substituted benzaldehydes and arene intermediates 25

ESI 2.3 NMR spectra of α-hydroxyphosphine oxides 30

ESI 2.4 NMR spectra of monoacylphosphine oxides 36

ESI 3 Mass spectra 50

ESI 3.1 Mass spectra of all photoinitiators before and after irradiation at 395 nm 50

ESI 3.2 CID mass spectra....54

ESI 4 References....55

ESI 1 Experimental Details

ESI 1.1 Photoreactor Mass Spectrometry

Photoreactor Irradiation Source

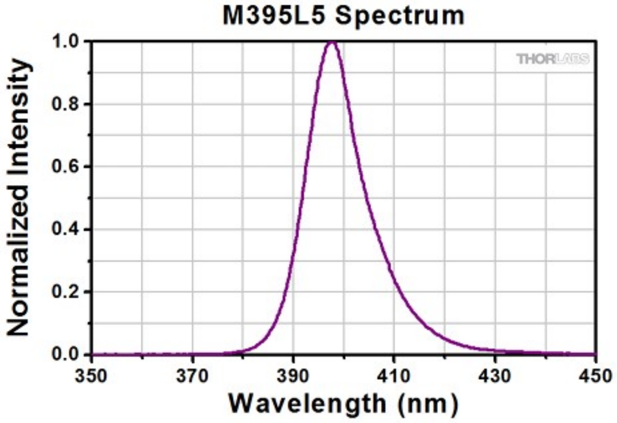

Figure S1. The emission spectrum of the 395 nm LED source.[1]

Polymerisation % Uncertainty

To assess the uncertainty in the Polymerisation % determination, triplicate measurements of **TPO** were performed across different days and each integrated over three separate time intervals. These measurements are summarised in Table S1. The total flow rates and the same photoinitiator concentrations were constant. Between the three triplicate measurements, one standard deviation in the polymerisation percent is ca. 2–4%. In addition, for each triplicate, the stability of the polymerisation percent across three-time intervals results is within a standard deviation of *ca*. 1%.

Table S1. Triplicate data of the polymerisation % of **TPO** measured across three days and three-time intervals after the reaction is initiated.

	2.5 – 5 mins	5 – 7.5 mins	7.5 – 10 mins	Average
Replicate I	88.1	87.5	89.5	88 ± 1 %
Replicate II	85.3	86.1	84.2	85 ± 1 %
Replicate III	89.6	89.4	91.0	90 ± 1 %
Average	88 ± 2 %	88 ± 2 %	88 ± 4 %	88 ± 2 %

Photostability of MAPOs 7 and 8

To further assess the photostability of MAPOs **7** and **8**, the change in the parent ion as a percent of the total ion counts was monitored with and without the LED, and the results are tabulated in Tables S2 and S3. For MAPO **7**, the parent percentage remains consistent before (51%) and after (49%) irradiation. MAPO **8** shows an increase in the percentage when the LED is on, however this is likely due to contaminants present in the spectrum when the LED is off. Since these contaminants were flushed during irradiation, MAPO **8** comprised a larger portion of the total ion count. Crucially, these results demonstrate that there is not a large reduction in the intensity of the parent peak when the LED is on for either photoinitiator, supporting the assignment that MAPOs **7** and **8** do not undergo photolysis and are largely photostable under these experimental conditions.

Table S2. The ion counts for the $[M+H]^+$ and $[2M+Na]^+$ ions of MAPO **7** as a percentage of the total ion count before and during irradiation.

MAPO **7**	*m/z* 400 $[M+Na]^+$ (%)	*m/z* 777 $[2M+Na]^+$ (%)	Sum (%)
LED395 Off	24	27	51
LED395 On	27	22	49

Table S3. The ion counts for the $[M+H]^+$ and $[2M+Na]^+$ ions of MAPO **8** as a percentage of the total ion count before and during irradiation.

MAPO **8**	*m/z* 436 $[M+Na]^+$ (%)	*m/z* 849 $[2M+Na]^+$ (%)	Sum (%)
LED395 Off	20	30	50
LED395 On	31	31	62

ESI 1.2 General Chemistry

Reagents and solvents

Solvents and celite 545 were purchased from Chemsupply Pty Ltd and were used as received unless otherwise specified. Anhydrous THF was obtained by pre-drying reagent grade THF over KOH overnight prior to distillation *in vacuo*. Distilled THF was stored over activated 3Å molecular sieves (20% v/v) for a minimum of 48 h before use. Molecular sieves were activated by heating in a furnace at >300 °C for a minimum of 24 h prior to use. All reagents were purchased from Chemsupply, Ambeed, Sigma-Aldrich or AK Scientific and used as received unless otherwise specified. 2,6-Dibromo-4-(trifluoromethyl)aniline was purchased from Ambeed. Diphenylphosphine oxide, di-*p*-tolylphosphine oxide, mesitaldehyde, 2,6-dimethyl-4-fluorobenzaldehyde, *N*,*N*,3,5-tetramethylaniline, 4-bromobenzotrifluoride, 4-bromofluorobenzene, and ethyl formate were purchased from AK Scientific. *N*-Bromosuccinimde, Mg for Grignard reactions, triethylamine (anhydrous), *N*,*N*-dimethylformamide (anhydrous), diethyl phosphite, ethyl phenylphosphinate, MnO_2 (activated, 85%, > 10 μm), and *n*-BuLi solution (2.5 M in hexanes) were purchased from Sigma-Aldrich. *n*-BuLi was titrated before use.[2] Deionised (RO) water was used for workup requiring extractions, and salt solutions such as brine were prepared as saturated solutions from commercially available salts. TPO was provided by industry partner, A&I Coatings Pty Ltd.

Reactions and purification

Glassware was cleaned using commercial detergent, acetone and, for removal of MnO_2, 36% HCl. Anhydrous and air-free conditions were achieved using Schlenk techniques previously described,[3] under a positive pressure of N_2. The N_2 used in reactions was passed through a 20 cm tube of anhydrous $CaCl_2$, which was prepared by heating $CaCl_2$ in a furnace at >300 °C for a minimum of 2 h before use. Cold reaction temperatures were achieved in an ice water bath (0 °C), or in a liquid nitrogen (LN_2)/EtOAc slurry (-84 °C) or LN_2/acetone slurry (-94 °C). Reactions at elevated temperatures were carried out in paraffin oil baths. Where sonication was required, a UC-S3200H Ultrasonic Cleaner with a 40 KHz frequency was used. Reactions were monitored by thin layer chromatography (TLC) on Merck silica gel 60 F_{254} aluminium-backed plates and were visualised using UV light at 254 nm and 365 nm. Purification by column chromatography was performed using silica gel 60 (40–63 μM, 230–400 mesh), under gravity elution or a positive pressure of air or N_2.

Characterisation

Infrared (IR) spectra were recorded in the absence of solvent using a Bruker Vertex 70 FTIR. Melting points were measured using a Buchi M-560 melting point apparatus. Electrospray ionisation (ESI) low resolution mass spectrometry (LRMS) for characterisation was performed using a Shimadzu LCMS-2020 spectrometer, and compounds were dissolved in MeOH. Where solubility was an issue in MeOH, CH_2Cl_2 was used as a co-solvent, and for compounds which were not readily ionisable, formic acid (10 μL) was added to the sample. ESI high resolution mass spectrometry (HRMS) was performed using a Waters QTOF Xevo. Ion mass to charge values are reported with their relative abundance, and bromine isotopes are indicated where appropriate. The molecular ion is denoted as M, and peaks are assigned to the molecular ion as $[M+H]^+$ or $[M+Na]^+$. ^1H, ^{13}C and ^{31}P nuclear magnetic resonance (NMR) spectra were acquired at 400, 100, and 162 MHz, respectively on a Bruker Ascend spectrometer. $CDCl_3$ was used with 0.05% v/v TMS. Where $CDCl_3$ was used as the NMR solvent, chemical shifts (δ) are reported relative to TMS (^1H δ = 0.00 ppm) and $CDCl_3$ (^{13}C δ = 77.16 ppm), and where DMSO-d_6 was used chemical shifts are reported relative to DMSO-d_6 (^1H δ = 2.50 ppm, ^{13}C δ = 39.52 ppm).

ESI 1.3 General Synthesis

Monoacylphosphine oxides (MAPOs) were synthesised as previously described, according to Scheme S1.[4]

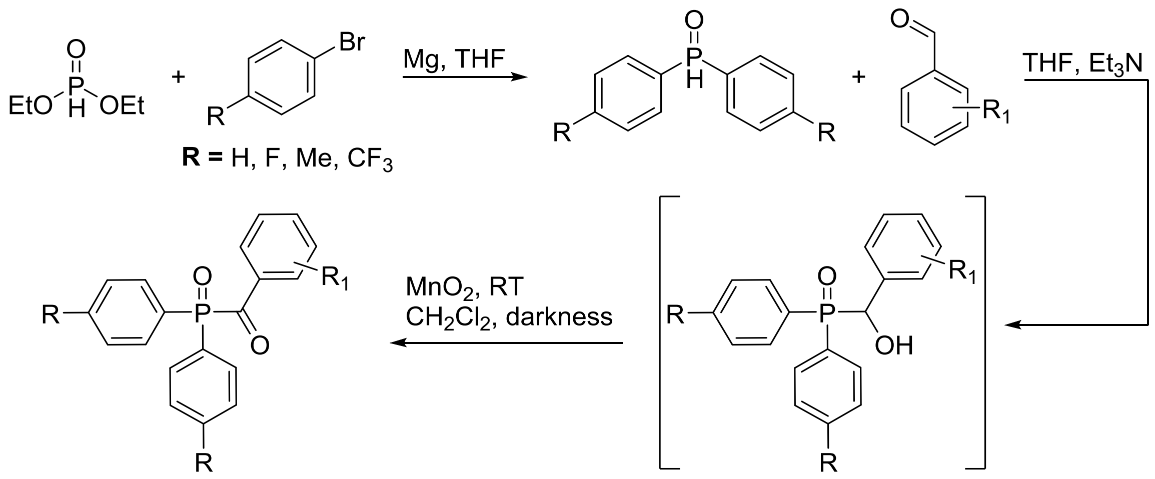

Scheme S1. Synthetic scheme to access substituted monoacylphosphine oxides.

ESI 1.4 Building Block Synthesis

Synthesis of secondary phosphine oxides

Secondary phosphine oxides were synthesised with modifications to literature reports (see below).[5]

bis(*p*-fluorophenyl)phosphine oxide (10)

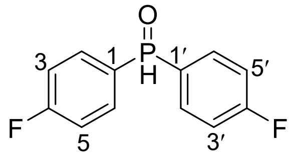

In a 25 mL Schlenk flask under N_2, a solution of 4-bromofluorobenzene (3.3 eq., 4.20 g, 24.0 mmol) in anhydrous THF (10 mL) was prepared. A 100 mL oven-dried two-neck round bottom flask was cooled under N_2 and fitted with a reflux condenser and magnetic stir bar. The flask was charged with Mg turnings (3.3 eq., 583 mg, 24.0 mmol) and then evacuated under reduced pressure and backfilled with N_2 (3x). Anhydrous THF (5 mL) was added by syringe, followed by the dropwise addition of 1 mL of the aryl bromide solution by cannulation. Upon initiation by sonication, the remaining aryl bromide solution was added dropwise to maintain reflux. The mixture was stirred for 1 h and cooled to 0 °C in an ice bath. A solution of diethyl phosphite (1 eq., 1.01 g, 7.27 mmol) in anhydrous THF (8 mL) was added dropwise at 0 °C over 15 minutes. The ice bath was removed, and the reaction was stirred at RT for a further 2 h. The mixture was again cooled to 0 °C and slowly quenched with 1 M HCl (30 mL). The white precipitate was filtered, and the filtrate was extracted with $CHCl_3$ (3 x 30 mL). The combined organic layers were washed with brine (2 x 30 mL), dried (Na_2SO_4), and concentrated to dryness *in vacuo*. The crude material was purified by column chromatography (3% v/v MeOH in CH_2Cl_2) to afford the product as a clear, viscous oil (4.90 g 85% yield). Spectral data is in accordance with literature.[5] $R_f = 0.26$ (3% MeOH in CH_2Cl_2). ^{1}H NMR (400 MHz, $CDCl_3$) δ 8.72 (d, $^1J_{H\text{-}P}$ = 485.2 Hz, 1H, PH), 7.72 (ddd, J = 13.1, 8.7, 5.5 Hz, 4H, H2, H6, H2′, and H6′), 7.21 (td, J = 8.7, 2.1 Hz, 4H, H3, H5, H3′, and H5′). ^{13}C NMR (101 MHz, $CDCl_3$) δ 165.5 (dd, $^1J_{C\text{-}F}$ = 254.5, $^4J_{C\text{-}P}$ = 3.3 Hz, C4 and C4′), 133.3 (dd, $^2J_{C\text{-}P}$ = 13.1, $^3J_{C\text{-}F}$ = 9.0 Hz, C2, C6, C2′, and C6′), 127.2 (dd, $^1J_{C\text{-}P}$ = 104.8, $^4J_{C\text{-}F}$ = 3.4 Hz, C1 and C1′), 116.5 (dd, $^2J_{C\text{-}F}$ = 21.7, $^3J_{C\text{-}P}$ = 14.2 Hz, C3, C5, C3′, and C5′). ^{31}P NMR (162 MHz, $CDCl_3$) δ 18.60. LRMS (ESI^+) *m/z* 239 (35%) $[M+H]^+$.

bis(*p*-(trifluoromethyl)phenyl)phosphine oxide (11)

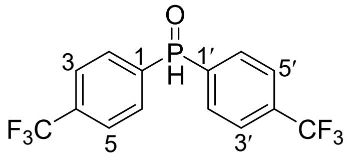

In a 25 mL Schlenk flask under N_2, a solution of 4-bromobenzotrifluoride (3.3 eq., 1.12 g, 4.98 mmol) in anhydrous THF (5 mL) was prepared. A separate 25 mL oven-dried Schlenk flask was cooled under N_2 and fitted with a magnetic stir bar. The flask was charged with Mg turnings (3.3 eq., 121 mg, 4.98 mmol) and then evacuated under reduced pressure and backfilled with N_2 (3x). Anhydrous THF (1 mL) was added by syringe, and the suspension was cooled to 0 °C in an ice bath. The solution of aryl bromide was added slowly by cannulation, and the reaction was stirred for 15 min at 0 °C. The ice bath was removed, and the reaction stirred at RT for a further 4 h. The reaction was cooled to 0 °C and a solution of diethyl phosphite (1 eq., 208 mg, 1.51 mmol) in anhydrous THF (2 mL) was added dropwise. The ice bath was removed, and the reaction was stirred at RT for a further 2 h. The mixture was again cooled to 0 °C and slowly quenched with 1 M HCl (5 mL) and the reaction was extracted with $CHCl_3$ (3 x 20 mL). The combined organic layers were washed with brine (2 x 20 mL), dried (Na_2SO_4), and concentrated to dryness *in vacuo*. The crude material was partially purified by column chromatography (40% v/v EtOAc in hexane to 60% v/v EtOAc in hexane) to obtain a yellow oil in 56% crude yield (284 mg), which was used without further purification in the next step. R_f = 0.30 (40% EtOAc in hexane). ^{1}H NMR (400 MHz, $CDCl_3$) δ 8.82 (d, J = 492.6 Hz, 1H, PH), 7.88 (dd, J = 13.3, 8.0 Hz, 4H, H2, H6, H2′, and H6′), 7.80 (dd, J = 8.4, 2.6 Hz, 4H, H3, H5, H3′, and H5′). ^{13}C NMR (101 MHz, $CDCl_3$) δ 134.5 (qd, $^2J_{C\text{-}F}$ = 32.8, $^4J_{C\text{-}P}$ = 3.0 Hz, C4 and C4′), 134.2 (d, $^1J_{C\text{-}P}$ = 100.2 Hz, C1 and C1′), 131.2 (d, $^2J_{C\text{-}P}$ = 12.0 Hz, C2, C6, C2′, and C6′), 126.1 (dq, $^3J_{C\text{-}P}$ = 13.2, $^3J_{C\text{-}F}$ = 3.8 Hz, C3, C5, C3′, and C5′). 123.8 (q, $^1J_{C\text{-}F}$ = 275.8 Hz, 2 x CF_3). ^{31}P NMR (162 MHz, $CDCl_3$) δ 17.85. LRMS (ESI$^-$) *m/z* 337 (100%) $[M\text{-}H]^-$. *Significant tautomerisation is observed, therefore duplicate resonances are present in the NMR spectra. Assigned resonances reported are concordant with literature values.[5]

phenyl(4-(trifluoromethyl)phenyl)phosphine oxide (12)

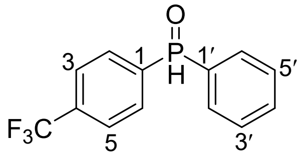

In a 25 mL Schlenk flask under N_2, a solution of 4-bromobenzotrifluoride (2 eq., 1.13 g, 5.02 mmol) in anhydrous THF (5 mL) was prepared. A separate 25 mL oven-dried Schlenk flask was cooled under N_2 and fitted with a magnetic stir bar. The flask was charged with Mg turnings (2 eq., 122 mg, 5.27 mmol), evacuated under reduced pressure and

backfilled with N_2 (3x). Anhydrous THF (1 mL) was added by syringe, and the suspension was cooled to 0 °C in an ice bath. The solution of aryl bromide was added slowly by cannulation, and the reaction stirred for 15 min at 0 °C. The ice bath was removed, and the reaction was stirred at RT for a further 4 h. The reaction was cooled to 0 °C and a solution of ethyl phenylphosphinate (1 eq., 425 mg, 2.50 mmol) in anhydrous THF (2 mL) was added dropwise by syringe. The ice bath was removed, and the reaction was stirred at RT for a further 16 h. The mixture was again cooled to 0 °C and slowly quenched with 1 M HCl (5 mL) and the reaction was extracted with $CHCl_3$ (3 x 20 mL). The combined organic layers were washed with brine (2 x 20 mL), dried (Na_2SO_4), and concentrated to dryness *in vacuo*. The crude material was partially purified by column chromatography (10% v/v hexane in EtOAc) to obtain a yellow oil in 24% crude yield (324 mg), which was used without further purification in the next step. Spectral data is in accordance with literature.[6] R_f = 0.41 (10% hexane in EtOAc). ^{1}H NMR (400 MHz, $CDCl_3$) δ 8.74 (d, $^1J_{H\text{-}P}$ = 486.2 Hz, 1H, PH), 7.85 (dd, J = 13.0, 8.0 Hz, 2H, H2, and H6, or H3 and H5), 7.76 (dd, J = 8.4, 2.5 Hz, 2H, H3′ and H5′), 7.73 – 7.67 (m, 2H, H2, and H6, or H3 and H5), 7.61 (dt, J = 7.3, 3.7 Hz, 1H, H4′), 7.53 (dt, J = 7.3, 3.2 Hz, 2H, H2′ and H6′). ^{13}C NMR (101 MHz, $CDCl_3$) δ 135.8 (d, $^1J_{C\text{-}P}$ = 98.4 Hz, C1′), 134.3 (qd, $^2J_{C\text{-}F}$ = 32.8, $^4J_{C\text{-}P}$ = 2.9 Hz, C4), 133.1 (d, $^4J_{C\text{-}P}$ = 2.9 Hz, C4′), 131.2 (d, $J_{C\text{-}P}$ = 11.7 Hz, C2′ and C6′, or C3′ and C5′), 130.9 (d, $^1J_{C\text{-}P}$ = 103.2 Hz, C1), 130.6 (d, $J_{C\text{-}P}$ = 11.7 Hz, C2′ and C6′, or C3′ and C5′), 129.2 (d, $^3J_{C\text{-}P}$ = 13.1 Hz, C2 and C6), 125.8 (dq, $^4J_{C\text{-}P}$ = 12.9, $^3J_{C\text{-}F}$ = 3.8 Hz, C3 and C5), 123.4 (q, $^1J_{C\text{-}F}$ = 274.5 Hz, CF_3). ^{31}P NMR (162 MHz, $CDCl_3$) δ 19.72. LRMS (ESI^+) *m/z* 303 (45%) $[M+H+MeOH]^+$.

Synthesis of substituted benzaldehydes

Synthesis of 2,4,6-trisubstituted benzaldehydes was achieved based on modified literature procedures.[7, 8]

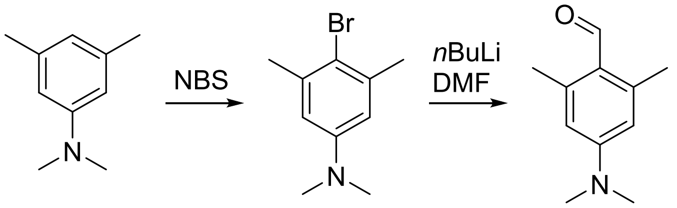

Scheme S2. Reaction scheme to produce 4-(dimethylamino)-2,6-dimethylbenzaldehyde.

4-bromo-*N*,*N*,3,5-tetramethylaniline (13)

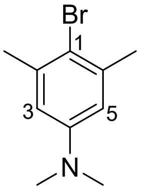

A solution of *N*,*N*,3,5-tetramethylaniline (1 eq., 1.00 g, 6.70 mmol) in anhydrous MeCN (15 mL) was cooled to 0 °C in an ice bath. A solution of NBS (1 eq., 1.19 g, 6.70 mmol) in anhydrous MeCN (15 mL) was added to the aniline dropwise over 15 min. The ice bath was removed, and the reaction stirred at RT for 16 h. The reaction mixture was evaporated to dryness, and the oily residue was dissolved in CH_2Cl_2 (60 mL) and washed with brine (2 x 30 mL). The organic layer was dried over Na_2SO_4, filtered, and the filtrate was concentrated *in vacuo*. The crude material was purified by column chromatography (45% v/v CH_2Cl_2 in hexane to 50% v/v CH_2Cl_2 in hexane) to afford 4-bromo-*N*,*N*,3,5-tetramethylaniline as a clear oil (1.41 g, 92%). Spectral data is in accordance with literature.[9] R_f = 0.71 (50% hexane in CH_2Cl_2). ^{1}H NMR (400 MHz, $CDCl_3$) δ 6.46 (s, 2H, H2 and H6), 2.88 (s, 6H, 2 x NCH_3), 2.36 (s, 6H, 2 x CH_3). ^{13}C NMR (101 MHz, $CDCl_3$) δ 149.3 (C1), 138.3 (C3 and C5), 114.6 (C4), 112.8 (C2 and C6), 40.7 (2 x NCH_3), 24.3 (2 x CH_3). LRMS (ESI$^+$) *m/z* 228 (50%) [^{79}Br, M+H]$^+$, 230 (50 %) [^{81}Br, M+H]$^+$.

4-(dimethylamino)-2,6-dimethylbenzaldehyde (14)

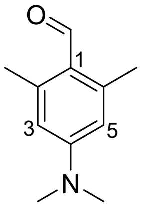

To a dried 25 mL Schlenk flask with a magnetic stir bar was added 4-bromo-*N*,*N*,3,5-tetramethylaniline (1 eq., 687 mg, 2.99 mmol). The flask was evacuated and backfilled with N_2 (3x). Anhydrous THF (15 mL) was added by syringe and the clear solution was cooled to -84 °C in an EtOAc/LN_2 cold bath. *n*BuLi (2.02 M in hexanes) (2 eq., 2.90 mL) was added dropwise over 15 min and the solution was stirred for 1 h at -84 °C before the dropwise addition of anhydrous DMF (2 eq., 439 mg, 6 mmol). The cold bath was removed, and the reaction stirred at RT for 30 min. The reaction was exposed to air, 1 M HCl (5 mL) was added, and the aqueous layer was extracted with Et_2O (2 x 20 mL). The combined organic layers were washed with brine (2 x 20 mL) and dried over Na_2SO_4. The solvent was removed *in vacuo*, and the crude product was purified by column chromatography (80% v/v CH_2Cl_2 in hexane to 100% CH_2Cl_2) to afford the aldehyde as an off-white solid (530 mg, 67%). Spectral data is in accordance with literature.[7] R_f = 0.50 (80% hexane in CH_2Cl_2). ^{1}H NMR (400 MHz, $CDCl_3$) δ 10.36 (s, 1H, HCO), 6.31 (s, 2H, H3 and H5), 3.05 (s, 6H, 2 x NCH_3), 2.60 (s, 6H, 2 x CH_3). ^{13}C NMR (101 MHz, $CDCl_3$) δ 190.6 (CO), 152.9 (C4), 144.2 (C2 and C6), 121.6 (C1), 112.0 (C3 and C5), 39.9 (2 x NCH_3), 21.6 (2 x CH_3). LRMS (ESI$^+$) *m/z* 178 (45%) [M+H]$^+$.

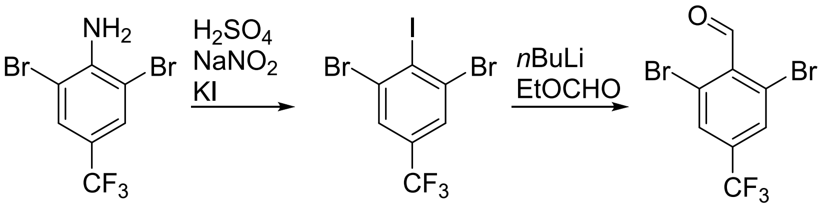

Scheme S3. Reaction scheme to produce 2,6-dibromo-4-(trifluoromethyl)benzaldehyde.

1,3-dibromo-2-iodo-5-(trifluoromethyl)benzene (15)

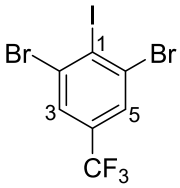

In a 100 mL RBF, a solution of 2,6-dibromo-4-(trifluoromethyl)aniline (1 eq., 765 mg, 2.40 mmol) in glacial acetic acid (4 mL) was cooled to 0 °C in an ice bath. In a separate ice bath, $NaNO_2$ (1.6 eq., 262 mg, 3.8 mmol) was added to conc. H_2SO_4 (2 mL) portion wise. The suspension was added to the aniline mixture dropwise over 10 min at 0 °C. The ice bath was removed, and the reaction was stirred at RT for 16 h. After this time all solids had dissolved, and the reaction mixture was a pale-yellow solution. Excess nitrosonium ion was quenched slowly with urea (3.6 eq., 509 mg, 8.48 mmol). An aqueous solution of KI (1.4 eq., 565 mg, 3.40 mmol in 5 mL H_2O) was added to the reaction mixture dropwise and the solution turned dark brown. The reaction stirred at 85 °C for 1 h. Once cooled to RT, the product was extracted into CH_2Cl_2 (3 x 25 mL), and the combined organic layers were washed with saturated NaS_2O_5 (25 mL). The organic layer was washed with 1 M HCl (2 x 25 mL), washed with brine (1 x 25 mL), dried over Na_2SO_4, and concentrated *in vacuo*. This produced the title compound as an orange crystalline solid (773 mg, 75%). R_f = 0.86 (30% EtOAc in hexane). mp: 47 °C. FTIR (neat): ν_{max} 3093 (w), 1303 (s), 1118 (s), 1094 (s), 739 (m), 702 (s), 694 (m). ^{1}H NMR (400 MHz, $CDCl_3$) δ 7.78 (d, $^4J_{H\text{-}F}$ = 0.8 Hz, 1H, H4 and H6). ^{13}C NMR (101 MHz, $CDCl_3$) δ 132.7 (q, $^2J_{C\text{-}F}$ = 34.0 Hz, C5), 132.1 (C2), 127.6 (q, $^3J_{C\text{-}F}$ = 3.7 Hz, C4 and C6), 122.3 (q, $^1J_{C\text{-}F}$ = 273.3 Hz, CF_3), 114.6 (d, $^4J_{C\text{-}F}$ = 1.5 Hz, C3 and C5). ^{19}F NMR (376 MHz, $CDCl_3$) δ -63.08. LRMS (EI) *m/z* 428 (19%) [^{79}Br, ^{79}Br, M]$^{\bullet+}$, 430 (40%) [^{79}Br, ^{81}Br, M]$^{\bullet+}$, 432 (20%) [^{81}Br, ^{81}Br, M]$^{\bullet+}$. HRMS (ASAP) M+H not found.

2,6-dibromo-4-(trifluoromethyl)benzaldehyde (16)

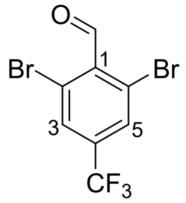

To a dried 25 mL Schlenk flask with a magnetic stir bar was added 1,3-dibromo-2-iodo-5-(trifluoromethyl)benzene (1 eq., 430 mg, 0.923 mmol). The flask was evacuated and backfilled with N_2 (3x). Anhydrous THF (5 mL) was added by syringe and the pale pink solution was cooled to -84 °C in an EtOAc/LN_2 bath. *n*BuLi (2.02 M in hexanes) (1 eq., 495 μL) was added dropwise and the reaction was left to stir at -84 °C for 15 min. Ethyl formate (2 eq., 148 mg, 2.00 mmol) was added dropwise by syringe, the cooling bath was removed, and the reaction stirred at RT for 30 min. The reaction was exposed to air, 1 M HCl (5 mL) was added, and the aqueous layer was extracted with Et_2O (2 x 20 mL). The combined organic layers were washed with brine (2 x 20 mL), dried over Na_2SO_4, and dried *in vacuo*. The crude product was purified by column chromatography (10% v/v CH_2Cl_2 in hexane) to afford the aldehyde (175 mg, 53%) as a pale-yellow oil. FTIR (neat): ν_{max} 3498 (w), 2875 (b/w), 1715 (m), 1305 (s), 1095 (s), 740 (m), 693 (m). ^{1}H NMR (400 MHz, $CDCl_3$) δ 10.23 (s, 1H, HCO), 7.89 (s, 2H, H3 and H5). ^{13}C NMR (101 MHz, $CDCl_3$) δ 190.1 (CO), 136.2 (C1), 135.3 (q, $^2J_{C\text{-}F}$ = 34.2 Hz, C4), 130.4 (q, $^3J_{C\text{-}F}$ = 3.7 Hz, C3 and C5), 124.6 (C2 and C6), 120.4 (d, $^1J_{C\text{-}F}$ = 274.2 Hz, CF_3). ^{19}F NMR (376 MHz, $CDCl_3$) δ -63.43. LRMS (EI) *m/z* 329 (19%) [^{79}Br, ^{79}Br, M]$^{\bullet+}$, 331 (40%) [^{79}Br, ^{81}Br, M]$^{\bullet+}$, 333 (20%) [^{81}Br, ^{81}Br, M]$^{\bullet+}$. HRMS (ASAP) M+H not found.

ESI 1.5 General procedure A: Synthesis of α-hydroxyphosphine oxides

The α-hydroxyphosphine oxide precursors (**17**–**20**) for MAPOs **1**–**4** were synthesised as reported.[10] In a 25 mL round bottom flask equipped with a magnetic stir bar, *p*-substituted diphenylphosphine oxide (1 eq.) was dissolved in THF. The corresponding benzaldehyde (1 eq.) was added to the solution, followed by the addition of Et_3N (1 drop). The reaction was stirred at RT and monitored by TLC analysis until completion (2–24 h). The reaction mixture was evaporated to dryness *in vacuo* and subject to further purification to afford the corresponding α-hydroxyphosphine oxide.

ESI 1.6 General procedure B: Oxidation of α-hydroxyphosphine oxides to synthesise monoacylphosphine oxides

MAPOs **1**–**4** were synthesised in a similar fashion, by oxidation of their respective precursors (**17**–**20**). In a 25 mL round bottom flask equipped with a magnetic stir bar, α-hydroxyphosphine oxide (1 eq.) was dissolved in minimal CH_2Cl_2 and the flask was wrapped with aluminium foil.

Activated MnO_2 (20 eq.) was added, and the reaction was stirred at RT and was monitored by TLC analysis until completion (2–16 h). The suspension was filtered through a pad of celite and washed with CH_2Cl_2. The crude mixture was dried *in vacuo* and purified by silica gel column chromatography to afford products **1**–**4**.

ESI 1.7 General procedure C: Two-step synthesis of monoacylphosphine oxides

MAPOs **5**–**9** were synthesised over two steps, without isolation of the corresponding α-hydroxyphosphine oxide intermediates.[4] In a 25 mL round bottom flask equipped with a magnetic stir bar, *p*-substituted diphenylphosphine oxide (1 eq.) was dissolved in THF. The corresponding benzaldehyde (1 eq.) was added to the solution, followed by the addition of Et_3N (1 drop). The reaction was stirred at RT and monitored by TLC analysis until completion (2–24 h). The reaction mixture was evaporated to dryness *in vacuo*. The crude reaction mixture was dissolved in minimal CH_2Cl_2 and the flask was wrapped with aluminium foil. Activated MnO_2 (20 eq.) was added, and the reaction was stirred at RT and was monitored by TLC analysis until completion (2–16 h). The suspension was filtered through a pad of celite and washed with CH_2Cl_2. The crude mixture was dried *in vacuo* and purified by silica gel column chromatography to afford products **5**–**9**.

((4-fluoro-2,6-dimethylphenyl)(hydroxy)methyl)diphenylphosphine oxide (17)

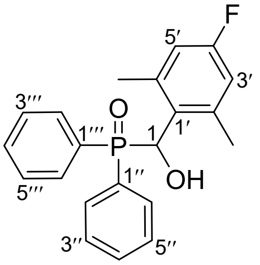

Compound **17** was synthesised according to general procedure A using diphenylphosphine oxide (1 eq., 216 mg, 1.07 mmol) and 4-fluoro-2,6-dimethylbenzaldehyde (1 eq., 163 mg, 1.07 mmol). The precipitate was filtered and washed with cold THF (3 x 5 mL) and hexane (2 mL), to afford **17** as a white amorphous solid (85%, 320 mg). mp: 106–107 °C. R_f = 0.18 (3% MeOH in CH_2Cl_2). FTIR (neat): ν_{max} 3114 (w), 1600 (w), 1477 (w). ^{1}H NMR (400 MHz, DMSO-d_6) δ 7.88 (ddd, $^3J_{H\text{-}P}$ = 10.4, J = 7.5, 1.4 Hz, 2H, H2″ and H6″, or H2‴ and H6‴), 7.72 – 7.60 (m, 3H, H2″, H4″, and H6″, or H2‴, H4‴, and H6‴), 7.60 – 7.50 (m, 3H, H3″, H4″, and H5″, or H3‴, H4‴, and H5‴), 7.49 – 7.41 (m, 2H, H3″ and H5″, or H3‴ and H5‴), 6.74 (d, $^3J_{H\text{-}F}$ = 9.7 Hz, 2H, H3′ and H5′), 6.27 (dd, $^3J_{H\text{-}P}$ = 20.7, J = 5.4 Hz, 1H, OH), 5.91 (dd, $^2J_{H\text{-}P}$ = 7.7, J = 5.4 Hz, 1H, H1), 2.11 (br s, 6H, 2 x CH_3). ^{13}C NMR (101 MHz, DMSO-d_6) δ 161.1 (dd, $^1J_{C\text{-}F}$ = 243.2, $^5J_{C\text{-}P}$ = 2.4 Hz, C4′), 133.7 (d, $^1J_{C\text{-}P}$ = 92.7 Hz, C1″ and C1‴), 132.7 (d, $^2J_{C\text{-}P}$ = 8.3 Hz, C2″ and C6″, or C2‴ and C6‴), 132.3 (d, $^4J_{C\text{-}P}$ = 2.8 Hz, C4″ or C4‴), 132.2 (d, $^4J_{C\text{-}P}$ = 2.7 Hz, C4″ or C4‴), 131.4 (d, $^2J_{C\text{-}P}$ = 8.8 Hz, C2″ and C6″, or C2‴ and C6‴), 130.6 (d, $^4J_{C\text{-}F}$ = 2.9 Hz, C1′), 128.8 (d, $^3J_{C\text{-}P}$ = 10.9 Hz, C3″, C5″, C3‴,

and C5‴), 70.5 (d, $^{1}J_{C\text{-}P}$ = 88.2 Hz, C1), 21.4 (2 x CH_3). ^{31}P NMR (162 MHz, DMSO-d_6) δ 27.83 (d, $^{6}J_{P\text{-}F}$ = 4.2 Hz). LRMS (ESI$^+$) *m/z* 355 (M+H, 65%). HRMS (ESI) *m/z* for $C_{20}H_{21}O_2FP$ $(M+H)^+$: calc. 355.1253; found 355.1263.

bis(4-fluorophenyl)(hydroxy(mesityl)methyl)phosphine oxide (18)

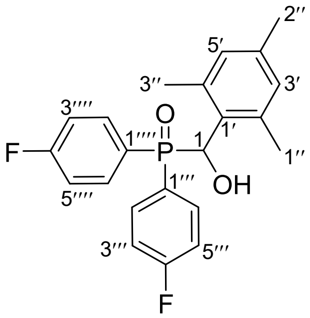

Compound **18** was synthesised according to general procedure A using bis(4-fluorophenyl)phosphine oxide (1 eq., 551 mg, 2.31 mmol) and mesitaldehyde (1 eq., 342 mg, 2.31 mmol). The solid was collected by vacuum filtration and washed with hexane (3 x 5 mL) to afford compound **18** as a white, chalky solid (87%, 751 mg). mp: 143–144 °C. R_f = 0.26 (3% MeOH in CH_2Cl_2). FTIR (neat): ν_{max} 3106 (w), 1588 (m). ^{1}H NMR (400 MHz, DMSO-d_6) δ 7.89 (ddd, $^{3}J_{H\text{-}P}$ = 10.0, J = 8.7, $^{4}J_{H\text{-}F}$ = 5.9 Hz, 2H, H2‴ and H6‴ or H2⁗ and H6⁗), 7.74 (ddd, $^{3}J_{H\text{-}P}$ = 10.0, J = 8.7, $^{4}J_{H\text{-}F}$ = 5.7 Hz, 2H, H2‴ and H6‴ or H2⁗ and H6⁗), 7.39 (td, $^{3}J_{H\text{-}F}$ and J = 9.0, $^{4}J_{H\text{-}P}$ = 2.1 Hz, 2H, H3‴ and H5‴ or H3⁗ and H5⁗), 7.31 (td, $^{3}J_{H\text{-}F}$ and J = 8.9, $^{4}J_{H\text{-}P}$ = 2.0 Hz, 2H, H3‴ and H5‴ or H3⁗ and H5⁗), 6.70 (s, 2H, H3′ and H5′), 6.32 (dd, $^{3}J_{H\text{-}P}$ = 21.7, J = 5.2 Hz, 1H, OH), 5.90 (dd, $^{2}J_{H\text{-}P}$ = 8.4, J = 5.2 Hz, 1H, H1), 2.17 (s, 3H, H2″), 2.04 (br s, 6H, H1″ and H3″). ^{13}C NMR (101 MHz, DMSO-d_6) δ 166.2 (dd, $^{1}J_{C\text{-}F}$ = 249.8, $^{4}J_{C\text{-}P}$ = 3.3 Hz, C4‴ or C4⁗), 165.9 (dd, $^{1}J_{C\text{-}F}$ = 249.8, $^{4}J_{C\text{-}P}$ = 3.0 Hz, C4‴ or C4⁗), 136.6 (d, $^{5}J_{C\text{-}P}$ = 2.8 Hz, C4′), 135.5 (t, $^{2}J_{C\text{-}P}$ and $^{3}J_{C\text{-}F}$ = 9.3 Hz, C2‴ and C6‴ or C2⁗ and C6⁗), 134.3 (t, $^{2}J_{C\text{-}P}$ and $^{3}J_{C\text{-}F}$ = 9.4 Hz C2‴ and C6‴ or C2⁗ and C6⁗), 130.8 (C1′), 130.2 (dd, $^{1}J_{C\text{-}P}$ = 94.8, $^{4}J_{C\text{-}F}$ = 3.3 Hz, C1‴ or C1⁗), 128.2 (dd, J = 94.5, 3.2 Hz, C1‴ or C1⁗), 116.2 (dd, $^{2}J_{C\text{-}F}$ = 11.8, $^{3}J_{C\text{-}P}$ = 7.1 Hz, C3‴ and C5‴ or C3⁗ and C5⁗), 116.0 (dd, $^{2}J_{C\text{-}F}$ = 11.8, $^{3}J_{C\text{-}P}$ = 7.1 Hz, C3‴ and C5‴ or C3⁗ and C5⁗), 71.0 (d, $^{1}J_{C\text{-}P}$ = 89.5 Hz, C1), 21.3 (C2″), 20.8 (C1″ and C3″). The resonance for C2′ and C6′ appears as a broad bump along the baseline spanning 131.5 – 127.5 ppm. ^{31}P NMR (162 MHz, DMSO) δ 26.7 (s). LRMS (ESI$^+$) *m/z* 387 (M+H, 30%). HRMS (ESI) *m/z* for $C_{22}H_{21}O_2F_2PNa$ $(M+Na)^+$: calc. 409.1145; found 409.1142.

((4-fluoro-2,6-dimethylphenyl)(hydroxy)methyl)bis(4-fluorophenyl)phosphine oxide (19)

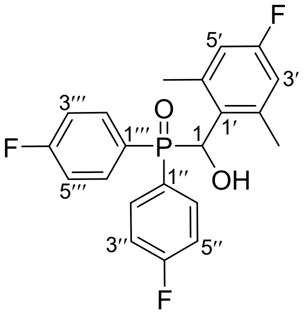

Compound **19** was synthesised according to general procedure A using di-*p*-tolylphosphine oxide (1 eq., 104 mg, 0.436 mmol) and 4-fluoro-2,6-dimethylbenzaldehyde (1 eq., 67.2 mg, 0.436 mmol). Purification by column chromatography (2% v/v MeOH in CH_2Cl_2 to 3% MeOH in CH_2Cl_2) afforded **19** as a white amorphous solid (84%, 142 mg). mp: 130–132 °C. R_f = 0.28 (3% MeOH in CH_2Cl_2). FTIR (neat): ν_{max} 3173 (w) 1589 (m). ^{1}H NMR (400 MHz, DMSO-d_6) δ 7.91 (ddd, $^3J_{H\text{-}P}$ = 10.0, J = 8.7, $^4J_{H\text{-}F}$ = 5.8 Hz, 2H, C2″ and C6″, or C2‴ and C6‴), 7.71 (ddd, $^3J_{H\text{-}P}$ = 10.1, J = 8.7, $^4J_{H\text{-}F}$ = 5.8 Hz, 2H, C2″ and C6″, or C2‴ and C6‴), 7.41 (td, J = 8.9, $^4J_{H\text{-}P}$ = 2.1 Hz, 2H, C3″ and C5″, or C3‴ and C5‴), 7.31 (td, J = 8.9, $^4J_{H\text{-}P}$ = 2.0 Hz, 2H, C3″ and C5″, or C3‴ and C5‴), 6.75 (d, $^3J_{H\text{-}F}$ = 9.7 Hz, 2H, H3′ and H5′), 6.39 (dd, $^3J_{H\text{-}P}$ = 21.3, J = 5.3 Hz, 1H, OH), 5.90 (dd, $^2J_{H\text{-}P}$ = 8.0, J = 5.2 Hz, 1H, H1), 2.10 (s, 6H, 2 x CH_3). ^{13}C NMR (101 MHz, DMSO-d_6) δ 164.5 (dd, $^1J_{C\text{-}F}$ = 250.2, $^4J_{C\text{-}P}$ = 3.3 Hz, C4″ or C4‴), 164.2 (dd, $^1J_{C\text{-}F}$ = 250.4, $^4J_{C\text{-}P}$ = 3.2 Hz), 160.7 (dd, $^1J_{C\text{-}F}$ = 243.3, $^5J_{C\text{-}P}$ = 2.8 Hz, C4′), 135.0 (t, $^2J_{C\text{-}P}$ and $^3J_{C\text{-}F}$ = 9.3 Hz, C3″ and C5″, or C3‴ and C5‴), 133.8 (t, $^2J_{C\text{-}P}$ and $^3J_{C\text{-}F}$ = 9.4 Hz, C3″ and C5″, or C3‴ and C5‴), 129.7 (d, $^4J_{C\text{-}F}$ = 2.9 Hz, C1′), 129.3 (dd, $^1J_{C\text{-}P}$ = 95.4, $^4J_{C\text{-}F}$ = 3.3 Hz, C1″ or C1‴), 127.4 (dd, $^1J_{C\text{-}P}$ = 95.4, $^4J_{C\text{-}F}$ = 3.2 Hz, C1″ or C1‴), 115.7 (dd, $^2J_{C\text{-}F}$ = 21.3, $^3J_{C\text{-}P}$ = 12.0 Hz, C3″ and C5″, or C3‴ and C5‴), 115.7 (dd, $^2J_{C\text{-}F}$ = 21.3, $^3J_{C\text{-}P}$ = 12.2 Hz, C3″ and C5″, or C3‴ and C5‴), 70.1 (d, $^1J_{C\text{-}P}$ = 89.6 Hz, C1), 20.9 (2 x CH_3). ^{31}P NMR (162 MHz, DMSO-d_6) δ 26.94 (d, $^6J_{P\text{-}F}$ = 4.4 Hz). LRMS (ESI$^+$) *m/z* 391 (M+H, 50%). HRMS (ESI) *m/z* for $C_{21}H_{18}O_2F_3PNa$ $(M+Na)^+$: calc. 413.0901; found 413.0894.

(hydroxy(mesityl)methyl)di-*p*-tolylphosphine oxide (20)

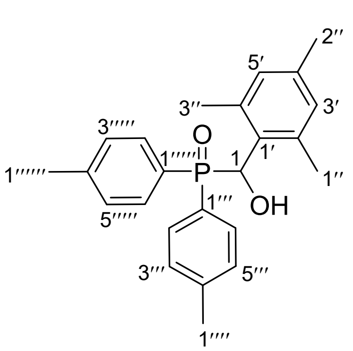

Compound **20** was synthesised according to general procedure A using di-*p*-tolylphosphine oxide (1 eq., 251 mg, 1.10 mmol) and mesitaldehyde (1 eq., 162 mg, 1.10 mmol). Purification by column chromatography (3% v/v MeOH in CH_2Cl_2) afforded compound **20** as an amorphous white solid (71% yield, 310 mg). Evidence of residual CH_2Cl_2 was observed in the ^{1}H NMR spectrum after drying under reduced pressure for 4 d. Therefore, the final yield was adjusted for the inclusion of 2 mol% CH_2Cl_2, determined by integration of

the methylene proton relative to the product resonances. mp: 120-121 °C. R_f = 0.21 (3% MeOH in CH_2Cl_2). FTIR (neat): ν_{max} 3136 (w), 1602 (w). ^{1}H NMR (400 MHz, DMSO-d_6) δ 7.72 (dd, $^3J_{H\text{-}P}$ = 10.3, J = 8.1 Hz, 2H, H2‴and H6‴, or H2′′′′′ and H6′′′′′), 7.52 (dd, $^3J_{H\text{-}P}$ = 10.4, J = 8.1 Hz, 2H, H2‴ and H6‴, or H2′′′′′ and H6′′′′′), 7.35 (dd, J = 8.2, $^4J_{H\text{-}P}$ = 2.1 Hz, 2H, H3‴ and H5‴, or H3′′′′′ and H5′′′′′), 7.24 (dd, J = 8.1, $^4J_{H\text{-}P}$ = 2.4 Hz, 2H, H3‴ and H5‴, or H3′′′′′ and H5′′′′′), 6.69 (s, 2H, H3′ and H5′) 6.10 (dd, $^3J_{H\text{-}P}$ = 20.7, J = 5.4 Hz, 1H, OH), 5.86 (dd, $^2J_{H\text{-}P}$ = 8.2, J = 5.4 Hz, 1H, H1), 2.39 (s, 3H, H1⁗ or H1′′′′′′), 2.32 (s, 3H, H1⁗ or H1′′′′′′), 2.18 (s, 3H, H2″), 2.06 (br s, 6H, H1″ and H3″). ^{13}C NMR (101 MHz, DMSO-d_6) δ 142.0 (d, $^4J_{C\text{-}P}$ = 2.6 Hz, C4‴ or C4′′′′′), 141.8 (d, $^4J_{C\text{-}P}$ = 2.8 Hz, C4‴ or C4′′′′′), 136.3 (d, $^5J_{C\text{-}P}$ = 2.6 Hz, C4′), 132.7 (d, $^2J_{C\text{-}P}$ = 8.6 Hz, C2‴ and C6‴ or C2′′′′′ and C6′′′′′), 131.7 (d, $^1J_{C\text{-}P}$ = 94.4 Hz, C1‴ or C1′′′′′), 131.54 (C1′), 131.47 (d, $^2J_{C\text{-}P}$ = 8.8 Hz, C2‴ and C6‴ or C2′′′′′ and C6′′′′′), 129.7 (d, J = 94.2 Hz, C1‴ or C1′′′′′), 129.4 (d, $^3J_{C\text{-}P}$ = 10.9 Hz, C3‴ and C5‴ or C3′′′′′ and C5′′′′′), 129.3 (d, $^3J_{C\text{-}P}$ = 11.2 Hz, C3‴ and C5‴ or C3′′′′′ and C5′′′′′), 71.0 (d, $^1J_{C\text{-}P}$ = 88.3 Hz, C1), 21.6 (C1⁗ or C1′′′′′′), 21.5 (C1⁗ or C1′′′′′′), 21.4 (C2″), 20.9 (C1″ and C3″). ^{31}P NMR (162 MHz, DMSO) δ 27.8 (s). LRMS (ESI$^+$) *m/z* (M+H, 100%) 379. HRMS (ESI) *m/z* for $C_{24}H_{27}O_2NaP$ (M+Na)$^+$: calc.401.1639; found 401.1646.

(diphenylphosphoryl)(4-fluoro-2,6-dimethylphenyl)methanone (1)

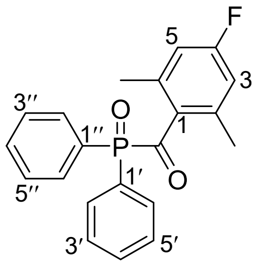

Compound **1** was synthesised according to general procedure B using **17** (1 eq., 302 mg, 0.853 mmol) and activated MnO_2 (20 eq., 1.49 g, 17.1 mmol). The reaction was carried out using a suspension of **17** in CH_2Cl_2 due to solubility issues. Purification by column chromatography (1% v/v MeOH in CH_2Cl_2 to 3% v/v MeOH in CH_2Cl_2) afforded compound **1** as a white amorphous solid (55%, 164 mg). mp: 104–106 °C. R_f = 0.58 (3% MeOH in CH_2Cl_2). FTIR (neat): ν_{max} 3058 (w), 1671 (m), 1588 (m). ^{1}H NMR (400 MHz, $CDCl_3$) δ 8.05 – 7.94 (m, 2H, H2′, H6′, H2″, and H6″), 7.63 – 7.55 (m, 1H, C4′ and C4″), 7.55 – 7.47 (m, 2H, H3′, H5′, H3″, and H5″), 6.71 (d, $^3J_{H\text{-}F}$ = 9.4 Hz, 1H, H3 and H5), 2.05 (s, 6H, 2 x CH_3). ^{13}C NMR (101 MHz, $CDCl_3$) δ 219.4 (d, $^1J_{C\text{-}P}$ = 73.7 Hz, CO), 163.4 (d, $^1J_{C\text{-}F}$ = 250.0 Hz, C4), 138.1 (d, $^3J_{C\text{-}F}$ = 9.2 Hz, C2 and C6), 135.1 (dd, $^2J_{C\text{-}P}$ = 40.1, $^4J_{C\text{-}F}$ = 3.0 Hz, C1), 132.6 (d, $^4J_{C\text{-}P}$ = 3.0 Hz, C4′ and C4″), 131.9 (d, $^2J_{C\text{-}P}$ = 8.8 Hz, C2′, C6′, C2″, and C6″), 129.8 (d, $^1J_{C\text{-}P}$ = 93.9 Hz, C1′ and C1″), 128.8 (d, $^3J_{C\text{-}P}$ = 11.9 Hz, C3′, C5′, C3″, and C5″), 115.0 (d, $^2J_{C\text{-}F}$ = 21.4 Hz, C3 and C5), 19.9 (d, $^4J_{C\text{-}F}$ = 1.7 Hz, 2 x CH_3). ^{31}P NMR (162 MHz, $CDCl_3$) δ 13.42. LRMS (ESI$^+$)

m/z 375 (28%) [M+Na]$^+$. HRMS (ESI$^+$) *m/z* for $C_{21}H_{18}FO_2PNa$ [M+Na]$^+$: calc. 375.0926, found 375.0916.

(bis(4-fluorophenyl)phosphoryl)(mesityl)methanone (2)

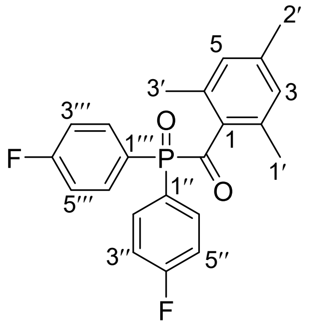

Compound **2** was synthesised according to general procedure B using **19** (1 eq., 248 mg, 0.642 mmol) and activated MnO_2 (20 eq., 1.12 g, 12.8 mmol). Purification by column chromatography (CH_2Cl_2 to 2% v/v MeOH in CH_2Cl_2) afforded compound **2** as a white amorphous solid (62%, 154 mg). Reported previously,[11] however characterisation data published is incomplete. mp 143–144 °C. R_f = 0.73 (2% MeOH in CH_2Cl_2). FTIR (neat): ν_{max} 3094 (w), 3054 (w), 1667 (m), 1609 (m). ^{1}H NMR (400 MHz, $CDCl_3$) δ 7.99 (ddd, $^3J_{H\text{-}P}$ = 10.6, J = 8.9, $^4J_{H\text{-}F}$ = 5.5 Hz, 4H, H2′′, H6′′, H2′′′, and H6′′′), 7.21 (ddt, J = 8.7, $^3J_{H\text{-}F}$ = 6.7, $^4J_{H\text{-}P}$ = 2.3 Hz, 4H, H3′′, H5′′, H3′′′, and H5′′′), 6.83 (s, 2H, H3 and H5), 2.27 (s, 3H, H2′), 2.02 (s, 6H, H1′ and H3′). ^{13}C NMR (101 MHz, $CDCl_3$) δ 219.8 (d, $^1J_{C\text{-}P}$ = 74.1 Hz, CO), 165.5 (dd, $^1J_{C\text{-}F}$ = 254.5, $^4J_{C\text{-}P}$ = 3.9 Hz, C4′′ and C4′′′), 141.0 (C4), 135.9 (d, $^2J_{C\text{-}P}$ = 40.5 Hz, C1), 134.9 (C2 and C6), 134.4 (dd, $^2J_{C\text{-}P}$ = 10.0, $^3J_{C\text{-}F}$ = 9.4 Hz, C2′′, C6′′, C2′′′, and C6′′′), 129.0 (C3 and C5), 125.5 (dd, $^1J_{C\text{-}P}$ = 96.6, $^4J_{C\text{-}F}$ = 3.2 Hz), 116.3 (dd, $^2J_{C\text{-}F}$ = 21.5, $^3J_{C\text{-}P}$ = 12.8 Hz), 21.2 (C2′), 19.7 (C1′ and C3′). ^{31}P NMR (162 MHz, $CDCl_3$) δ 11.28. LRMS (ESI$^+$) *m/z* 407 (30%) [M+Na]$^+$. HRMS (ESI$^+$) *m/z* for $C_{22}H_{19}F_2O_2PNa$ [M+Na]$^+$: calc. 407.0988, found 407.0987.

(bis(4-fluorophenyl)phosphoryl)(4-fluoro-2,6-dimethylphenyl)methanone (3)

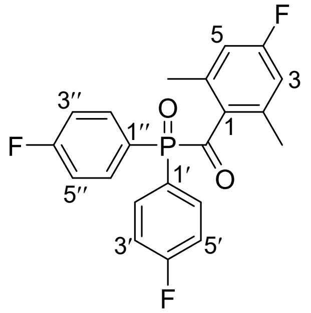

Compound **3** was synthesised according to general procedure B using **19** (1 eq., 62.4 mg, 0.159 mmol) and activated MnO_2 (20 eq., 270 mg, 3.18 mmol). Purification by column chromatography (CH_2Cl_2 to 2% v/v MeOH in CH_2Cl_2) afforded compound **3** as a white amorphous solid (33%, 20 mg). mp 132 °C. R_f= 0.33 (CH_2Cl_2). FTIR (neat): ν_{max} 3037 (w), 1674 (m), 1586 (m). ^{1}H NMR (400 MHz, $CDCl_3$) δ 7.99 (ddd, $^3J_{H\text{-}P}$ = 10.7, J = 8.8, $^4J_{H\text{-}F}$ = 5.5 Hz, 4H, H2′, H6′, H2′′, and H6′′), 7.23 (ddt, J = 8.7, $^3J_{H\text{-}F}$ = 6.7, $^4J_{H\text{-}P}$ = 2.4 Hz, 4H, H3′, H5′, H3′′, and H5′′), 6.73 (d, $^3J_{H\text{-}F}$ = 9.3 Hz, 2H, H3 and H5), 2.05 (s, 6H, 2 x CH_3). ^{13}C NMR (101 MHz, $CDCl_3$) δ 219.1 (d, $^1J_{C\text{-}P}$ = 75.5

Hz, CO), 166.9 (dd, $^{1}J_{C\text{-}F}$ = 257.2, $^{4}J_{C\text{-}P}$ = 3.6 Hz, C4′ and C4″), 163.9 (d, $^{1}J_{C\text{-}F}$ = 250.5 Hz, C4), 138.1 (d, $^{3}J_{C\text{-}F}$ = 9.1 Hz, C2 and C6), 134.9 (dd, $^{2}J_{C\text{-}P}$ = 39.9, $^{4}J_{C\text{-}F}$ = 3.5 Hz, C1), 134.4 (dd, $^{2}J_{C\text{-}P}$ = 9.8, $^{3}J_{C\text{-}F}$ = 9.2 Hz, C2′, C6′, C2″, and C6″), 125.1 (dd, $^{1}J_{C\text{-}P}$ = 97.2, $^{4}J_{C\text{-}F}$ = 3.3 Hz, C1′ and C1″), 116.5 (dd, $^{2}J_{C\text{-}F}$ = 21.6, $^{3}J_{C\text{-}P}$ = 12.9 Hz, C3′, C5′, C3″, and C5″), 115.1 (d, $^{2}J_{C\text{-}F}$ = 21.5 Hz, C3 and C5), 19.9 (d, $^{4}J_{C\text{-}F}$ = 1.8 Hz, 2 x CH_3). ^{31}P NMR (162 MHz, $CDCl_3$) δ 11.45. LRMS (ESI$^+$) *m/z* 389 (50%) [M+H]$^+$. HRMS (ESI$^+$) *m/z* for $C_{21}H_{17}F_3O_2P$ [M+H]$^+$: calc. 389.0918, found 389.0916.

(di-*p*-tolylphosphoryl)(mesityl)methanone (4)

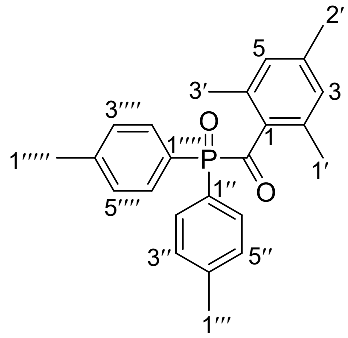

Compound **4** was synthesised according to general procedure B using **20** (1 eq., 147 mg, 0.388 mmol) and activated MnO_2 (20 eq., 674 mg, 7.76 mmol). Purification by column chromatography (2% v/v MeOH in CH_2Cl_2) afforded compound **4** as a white amorphous solid (79%, 116 mg). Previously reported,[12, 13] however full spectral data is not reported. mp 118–119 °C. R_f = 0.27 (2% MeOH in CH_2Cl_2). FTIR (neat): ν_{max} 2919 (w), 1668 (m), 1599 (m). ^{1}H NMR (400 MHz, $CDCl_3$) δ 7.85 (dd, $^{3}J_{H\text{-}P}$ = 11.0, J = 8.2 Hz, 4H, H2″, H6″, H2′′′′ and H6′′′′), 7.30 (dd, J = 8.2, $^{4}J_{H\text{-}P}$ = 2.7 Hz, 4H, H3″, H5″, H3′′′′ and H5′′′′), 6.80 (d, J = 1.1 Hz, 2H, H3 and H5), 2.40 (d, J = 0.9 Hz, 6H, H1′′′ and H1′′′′′), 2.26 (s, 3H, H2′), 2.03 (s, 6H, H1′ and H3′). ^{13}C NMR (101 MHz, $CDCl_3$) δ 220.6 (d, $^{1}J_{C\text{-}P}$ = 73.1 Hz, CO), 142.9 (d, $^{4}J_{C\text{-}P}$ = 2.8 Hz, C4″ and C4′′′′), 140.4 (C4), 136.5 (d, $^{2}J_{C\text{-}P}$ = 39.6 Hz, C1), 134.9 (C2 and C6), 131.9 (d, $^{2}J_{C\text{-}P}$ = 9.2 Hz, C2″, C6", C2′′′′ and C6′′′′), 129.5 (d, $^{3}J_{C\text{-}P}$ = 12.1 Hz, C3″, C5″, C3′′′′, and C5′′′′), 128.9 (C3 and C5), 126.6 (d, $^{1}J_{C\text{-}P}$ = 96.0 Hz, C1″ and C1′′′′), 21.6 (C1′′′ and C1′′′′′), 21.2 (C2′), 19.7 (C1′ and C3′). ^{31}P NMR (162 MHz, $CDCl_3$) δ 14.33. LRMS (ESI$^+$) *m/z* 399 (50%) [M+Na]$^+$. HRMS (ESI$^+$) *m/z* for $C_{24}H_{24}O_2PNa$ [M+Na]$^+$: calc. 399.1490, found 399.1480.

(bis(4-(trifluoromethyl)phenyl)phosphoryl)(mesityl)methanone (5)

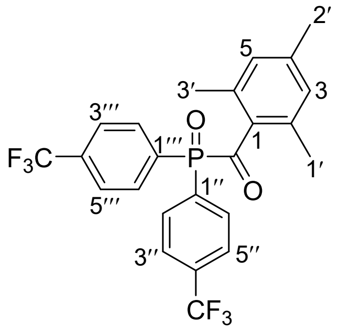

Compound **5** was synthesised according to general procedure A using bis(4-(trifluoromethyl)phenyl)phosphine oxide (**11**) (1 eq., 339 mg, 1.01 mmol) and mesitaldehyde (1 eq., 149 mg, 0.500 mmol). Purification by column chromatography (10% v/v hexane in CH_2Cl_2 to CH_2Cl_2) afforded compound **5** as a white amorphous solid (26% yield over two steps, 127 mg). Previously reported,[13] however full spectral data is not reported. mp 133–134 °C. R_f = 0.61 (10% hexane in CH_2Cl_2). FTIR (neat): ν_{max} 2925 (w), 1673 (w), 1609 (w). ^{1}H NMR (400 MHz, $CDCl_3$) δ 8.17 (dd, $^3J_{H\text{-}P}$ = 10.6, J = 8.1 Hz, 4H, H2″, H6″, H2‴, and H6‴), 7.79 (dd, J = 8.3, $^4J_{H\text{-}P}$ = 2.5 Hz, 4H, H3″, H5″, H3‴, and H5‴), 6.85 (s, 2H, H3 and H5), 2.28 (s, 3H, H2′), 2.04 (s, 6H, H1′ and H3′). ^{13}C NMR (101 MHz, $CDCl_3$) δ 218.5 (d, $^1J_{C\text{-}P}$ = 72.3 Hz, CO), 141.5 (C4), 135.4 (d, $^2J_{C\text{-}P}$ = 41.1 Hz, C1), 135.1 (C2 and C6), 134.5 (dd, $^2J_{C\text{-}F}$ = 32.7, $^4J_{C\text{-}P}$ = 3.2 Hz, C4″ and C4‴), 133.8 (d, $^1J_{C\text{-}P}$ = 90.5 Hz, C1″ and C1‴), 132.3 (d, $^2J_{C\text{-}P}$ = 8.9 Hz, C2″, C6″, C2‴, and C6‴), 129.2 (C3 and C5), 125.8 (dq, $^3J_{C\text{-}P}$ = 11.7, $^3J_{C\text{-}F}$ = 3.7 Hz, C3″, C5″, C3‴, and C5‴), 125.2 (q, $^1J_{C\text{-}F}$ = 273.0 Hz, 2 x CF_3), 21.3 (C2′), 19.9 (C1′ and C3′). ^{31}P NMR (162 MHz, $CDCl_3$) δ 8.33. LRMS (ESI$^+$) *m/z* 507 (30%) $[M+Na]^+$.

mesityl(phenyl(4-(trifluoromethyl)phenyl)phosphoryl)methanone (6)

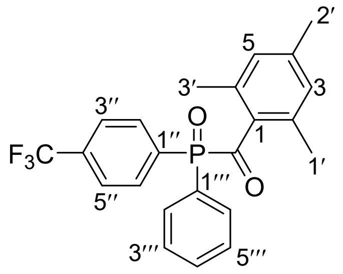

Compound **6** was synthesised according to general procedure A using phenyl(4-(trifluoromethyl)phenyl)phosphine oxide (**12**) (1 eq., 313 mg, 1.16 mmol) and mesitaldehyde (1 eq., 171 mg, 1.16 mmol). Purification by column chromatography (2% v/v MeOH in CH_2Cl_2) afforded compound **6** as a white amorphous solid (48% yield over two steps, 146 mg). mp 139–140 °C. R_f = 0.31 (2% MeOH in CH_2Cl_2). FTIR (neat): ν_{max} 2922 (w), 1667 (m), 1608 (m). ^{1}H NMR (400 MHz, $CDCl_3$) δ 8.23 – 8.11 (m, 2H, H2″ and H6″), 8.07 – 7.92 (m, 2H, H2‴ and H6‴), 7.77 (dd, J = 8.1, $^4J_{H\text{-}P}$ = 2.2 Hz, 2H, H3″ and H5″), 7.64 – 7.57 (m, 1H, H4‴), 7.53 (td, J = 7.4, $^4J_{H\text{-}P}$ = 3.1 Hz, 2H, H3‴ and H5‴), 6.83 (s, 2H, H3 and H5), 2.27 (s, 3H, H2'), 2.03 (s, 6H, H1′ and H3′). ^{13}C NMR (101 MHz, $CDCl_3$) δ 219.3 (d, $^1J_{C\text{-}P}$ = 76.2 Hz, CO), 141.1 (C4), 135.8 (d, $^2J_{C\text{-}P}$ = 40.3 Hz, C1), 135.0 (C2 and C6), 134.6 (d, $^1J_{C\text{-}P}$ = 96.1 Hz, C1″), 134.1 (dd, $^2J_{C\text{-}F}$ = 32.4, $^4J_{C\text{-}P}$ = 2.6 Hz, C4″), 132.8 (d, $^4J_{C\text{-}P}$ = 2.5 Hz, C4‴), 132.4 (d, $^2J_{C\text{-}P}$ = 8.8 Hz, C2″ and C6″), 131.7 (d, $^2J_{C\text{-}P}$ = 8.5 Hz, C2‴ and C6‴), 129.5 (d, $^1J_{C\text{-}P}$ = 93.2 Hz, C1‴), 129.0 (C3‴ and C5‴), 128.9 (C3 and C5), 125.5 (dq, $^3J_{C\text{-}P}$ = 11.5, $^3J_{C\text{-}F}$ = 3.8 Hz, C3″ and C5″), 123.5 (q, $^1J_{C\text{-}F}$ = 272.9 Hz, CF_3), 21.2 (C1′ and C3′), 19.8 (C2′). ^{31}P NMR (162 MHz, $CDCl_3$) δ 10.79. LRMS (ESI$^+$) *m/z* 439 (19%) [M+Na]$^+$. HRMS (ESI$^+$) *m/z* for $C_{23}H_{20}F_3O_2PNa$ [M+Na]$^+$: calc. 439.1051, found 439.1067.

(4-(dimethylamino)-2,6-dimethylphenyl)(diphenylphosphoryl)methanone (7)

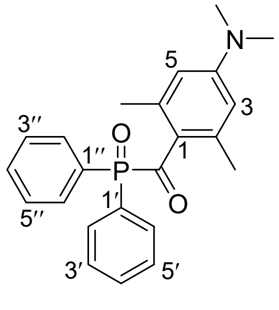

Compound **7** was synthesised according to general procedure A using diphenylphosphine oxide (1 eq., 114 mg, 0.564 mmol) and 4-(dimethylamino)-2,6-dimethylbenzaldehyde (**14**) (1 eq., 101 mg, 0.564 mmol). Purification by column chromatography (50% v/v hexane in EtOAc) afforded compound **7** as a yellow crystalline solid (14% yield over two steps, 29.7 mg). Previously reported,[9] however full spectral data is not reported. mp decomp. at 150 °C. R_f = 0.42 (50% hexane in EtOAc). FTIR (neat): ν_{max} 2919 (w), 1636 (m), 1593 (m). ^{1}H NMR (400 MHz, $CDCl_3$) δ 8.03 – 7.92 (m, 4H, H2′, H6′, H2″, and H6″), 7.57 – 7.51 (m, 2H, H4′ and H4‴), 7.51 – 7.45 (m, 4H, H3′, H5′, H3″, and H5″), 6.29 (s, 2H, H3 and H5), 2.96 (s, 6H, 2 x NCH_3), 2.15 (s, 6H, 2 x CH_3). ^{13}C NMR (101 MHz, $CDCl_3$) δ 217.0 (d, $^1J_{C\text{-}P}$ = 75.2 Hz, CO), 152.0 (C4), 138.1 (C2 and C6), 132.0 (d,

J = 2.8 Hz, C4′ and C4″), 131.9 (d, $^{2}J_{C\text{-}P}$ = 8.8 Hz, C2′, C6′, C2″, and C6″), 130.7 (d, $^{1}J_{C\text{-}P}$ = 92.9 Hz, C1′ and C1″), 128.6 (d, $^{3}J_{C\text{-}P}$ = 11.6 Hz, C3′, C5′, C3″, and C5″), 127.2 (d, $^{2}J_{C\text{-}P}$ = 42.7 Hz, C1), 111.5 (C3 and C5), 40.0 (2 x NCH_3), 21.0 (2 x CH_3). ^{31}P NMR (162 MHz, $CDCl_3$) δ 13.47. LRMS (ESI$^+$) *m/z* 400 (50%) $[M+Na]^+$. HRMS (ESI$^+$) *m/z* for $C_{23}H_{25}NO_2P$ $[M+H]^+$: calc. 378.1623, found 378.1613.

(bis(4-fluorophenyl)phosphoryl)(4-(dimethylamino)-2,6-dimethylphenyl)methanone (8)

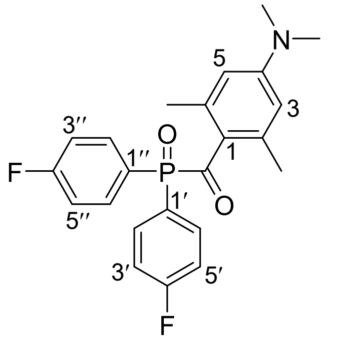

Compound **8** was synthesised according to general procedure A using bis(4-fluorophenyl)phosphine oxide (**10**) (1 eq., 162 mg, 0.677 mmol) and 4-(dimethylamino)-2,6-dimethylbenzaldehyde (**14**) (1 eq., 120 mg, 0.677 mmol). Purification by column chromatography (50% v/v hexane in EtOAc) afforded compound **8** as a yellow amorphous solid (42% yield over two steps, 118 mg). 143–144 °C. R_f = 0.37 (50% hexane in EtOAc). FTIR (neat): ν_{max} 2924 (w), 1591 (s), 1496 (m). ^{1}H NMR (400 MHz, $CDCl_3$) δ 7.98 (dddd, $^{3}J_{H\text{-}P}$ = 10.5, 8.8, $^{4}J_{H\text{-}F}$ = 5.5, 2.0 Hz, 4H, H2′, H6′, H2″ and H6″), 7.18 (ddt, J = 8.8, $^{3}J_{H\text{-}F}$ = 6.8, $^{4}J_{H\text{-}P}$ = 2.3 Hz, 4H, H3′, H5′, H3″, H5″), 6.30 (s, 2H, H3 and H5), 2.97 (s, 6H, 2 x NCH_3), 2.14 (s, 6H, 2 x CH_3). ^{13}C NMR (101 MHz, $CDCl_3$) δ 216.3 (d, $^{1}J_{C\text{-}P}$ = 76.8 Hz, CO), 165.3 (dd, $^{1}J_{C\text{-}F}$ = 254.1, $^{1}J_{C\text{-}P}$ = 3.4 Hz, C4′ and C4″), 152.1 (C4), 138.2 (C2 and C6), 134.4 (q, $^{2}J_{C\text{-}P}$ = 9.8, $^{3}J_{C\text{-}F}$ = 9.0 Hz, C2′, C6′, C2″, and C6″), 126.6 (dd, $^{1}J_{C\text{-}P}$ = 96.8, $^{4}J_{C\text{-}F}$ = 3.4 Hz, C1′ and C1″), 126.5 (d, $^{2}J_{C\text{-}P}$ = 43.5 Hz, C1), 116.1 (dd, $^{2}J_{C\text{-}F}$ = 21.4, $^{3}J_{C\text{-}P}$ = 12.6 Hz, C3′, C5′, C3″, and C5″), 111.5 (C3 and C5), 40.0 (2 x NCH_3), 21.1 (2 x CH_3). ^{31}P NMR (162 MHz, $CDCl_3$) δ 11.54. LRMS (ESI$^+$) *m/z* 436 (50%) $[M+Na]^+$. HRMS (ESI$^+$) *m/z* for $C_{23}H_{23}F_2NO_2P$ $[M+H]^+$: calc. 414.1434, found 414.1425.

(2,6-dibromo-4-(trifluoromethyl)phenyl)(diphenylphosphoryl)methanone (9)

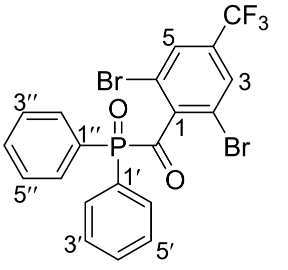

Compound **9** was synthesised according to general procedure A using diphenylphosphine oxide (1 eq., 62.6 mg, 0.309 mmol) and 2,6-dibromo-4-(trifluoromethyl)benzaldehyde (**16**) (1 eq., 103 mg, 0.309 mmol). Purification by column chromatography (30% v/v hexane in EtOAc) afforded compound **9** as an off-white amorphous solid (27% yield over two steps, 45.7 mg). 143–144 °C. R_f = 0.75 (30% hexane in EtOAc).

FTIR (neat): ν_{max} 3059 (w), 2925 (w), 1688 (w), 1546 (m). ^{1}H NMR (400 MHz, $CDCl_3$) δ 8.14 – 8.00 (m, 4H, H2′, H6′, H2″, and H6″), 7.77 (s, 2H, H3 and H5), 7.62 – 7.56 (m, 2H, H4′ and H4″), 7.56 – 7.49 (m, 4H, H3′, H5′, H3″, and H5″). ^{13}C NMR (101 MHz, $CDCl_3$) δ 212.3 (d, $^{1}J_{C\text{-}P}$ = 81.4 Hz, CO), 144.1 (d, $^{2}J_{C\text{-}P}$ = 39.3 Hz, C1), 134.4 (q, $^{2}J_{C\text{-}F}$ = 34.1 Hz, C4), 132.8 (d, $^{4}J_{C\text{-}P}$ = 3.0 Hz, C4′ and C4″), 131.7 (d, $^{2}J_{C\text{-}P}$ = 9.0 Hz, C2′, C6′, C2″, and C6″), 129.4 (d, $^{1}J_{C\text{-}P}$ = 93.7 Hz, C1′ and C1″), 128.8 (d, $^{3}J_{C\text{-}P}$ = 12.7 Hz, C3′, C5′, C3″, and C5″), 128.8 (C3, C5), 121.9 (q, $^{1}J_{C\text{-}F}$ = 273.8 Hz, CF_3), 119.6 (C2 and C6). ^{31}P NMR (162 MHz, $CDCl_3$) δ 12.66. Note, the resonance for C3 and C5 should be a quartet, however, resonances for C1′ and C1″, C3′, C5′, C3″, and C5″, C3, and C5 overlap, thus the coupling constant could not be calculated. LRMS (ESI^{+}) *m/z* 531 (19%) [^{79}Br, ^{79}Br, M+H]$^{+}$, 533 (40%) [^{79}Br, ^{81}Br, M+H]$^{+}$, 535 (20%) [^{81}Br, ^{81}Br, M+H]$^{+}$. HRMS (ESI^{+}) *m/z* for $C_{20}H_{13}{}^{79}Br_2F_3O_2P$ [M+H]$^{+}$: calc. 530.8972, found 530.8986.

ESI 2 NMR Characterisation

ESI 2.1 NMR spectra of secondary phosphine oxides

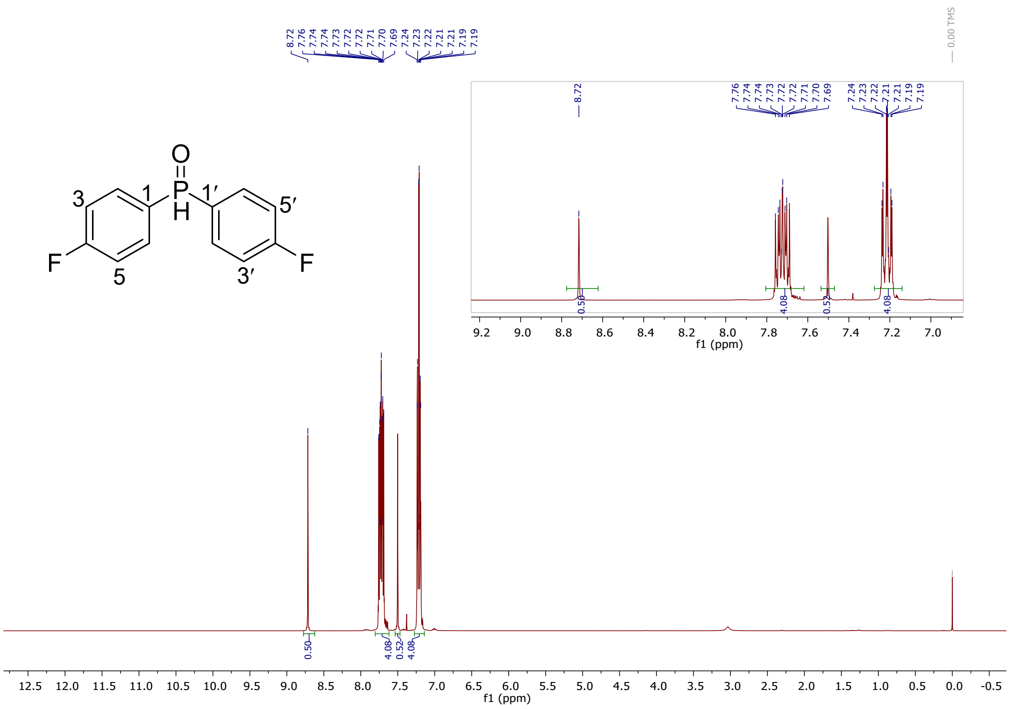

Figure S2. ^{1}H NMR spectrum of bis(*p*-fluorophenyl)phosphine oxide (**10**) ($CDCl_3$, 400 MHz).

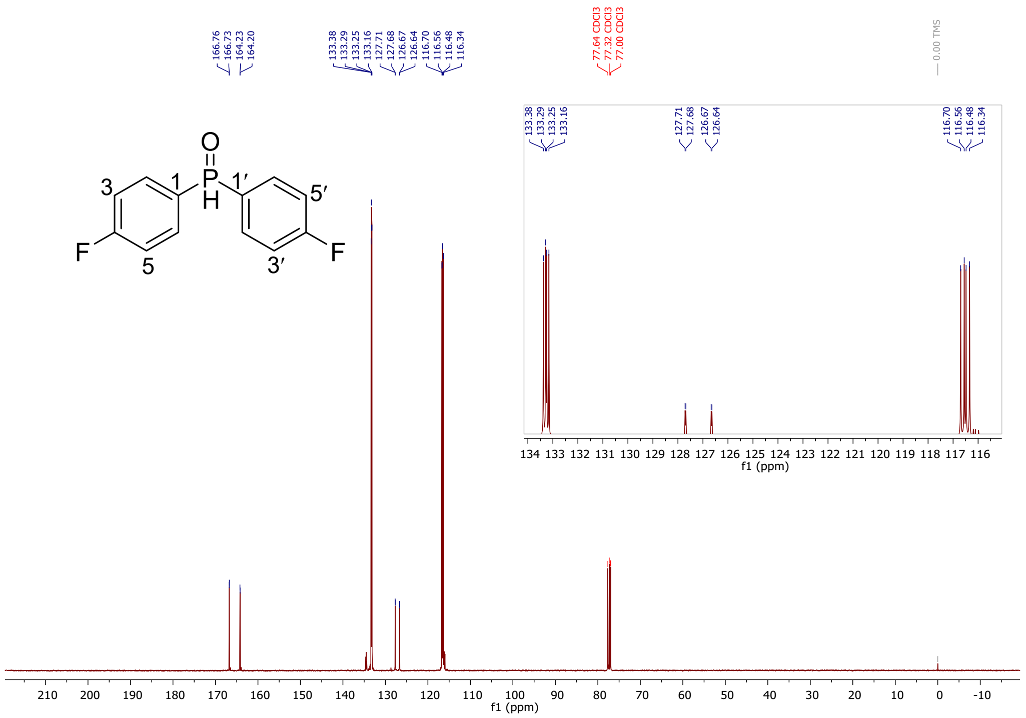

Figure S3. ^{13}C NMR spectrum of bis(*p*-fluorophenyl)phosphine oxide (**10**) ($CDCl_3$, 101 MHz).

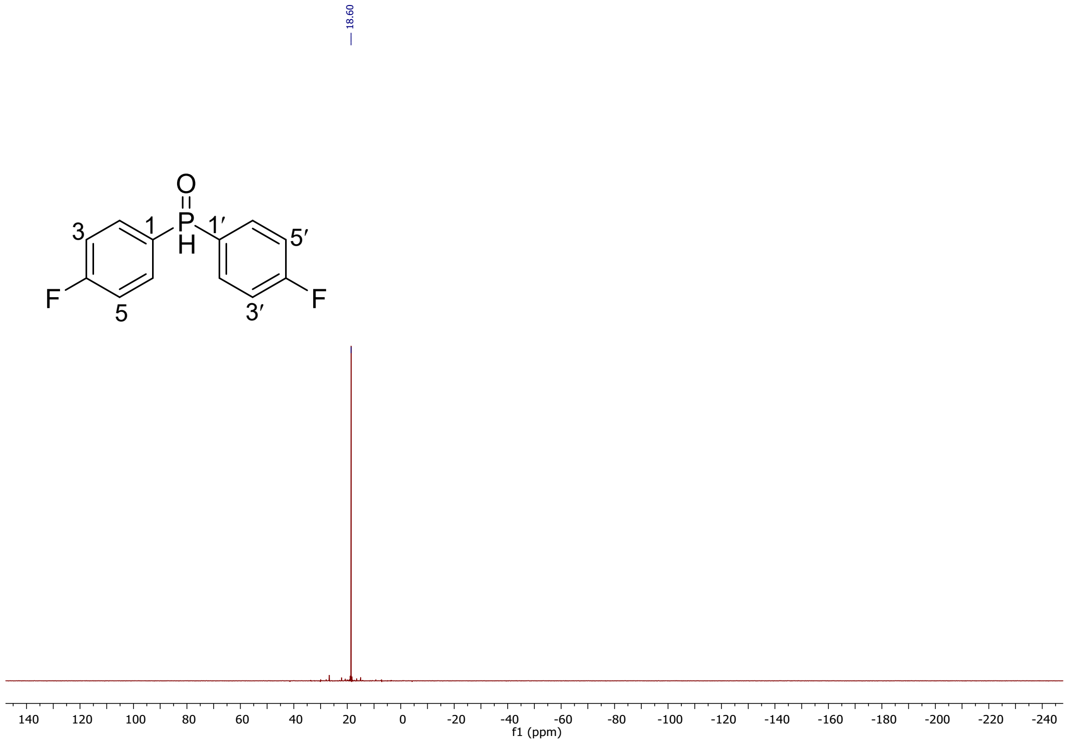

Figure S4. 3^{1}P NMR spectrum of bis(*p*-fluorophenyl)phosphine oxide (**10**) ($CDCl_3$, 162 MHz).

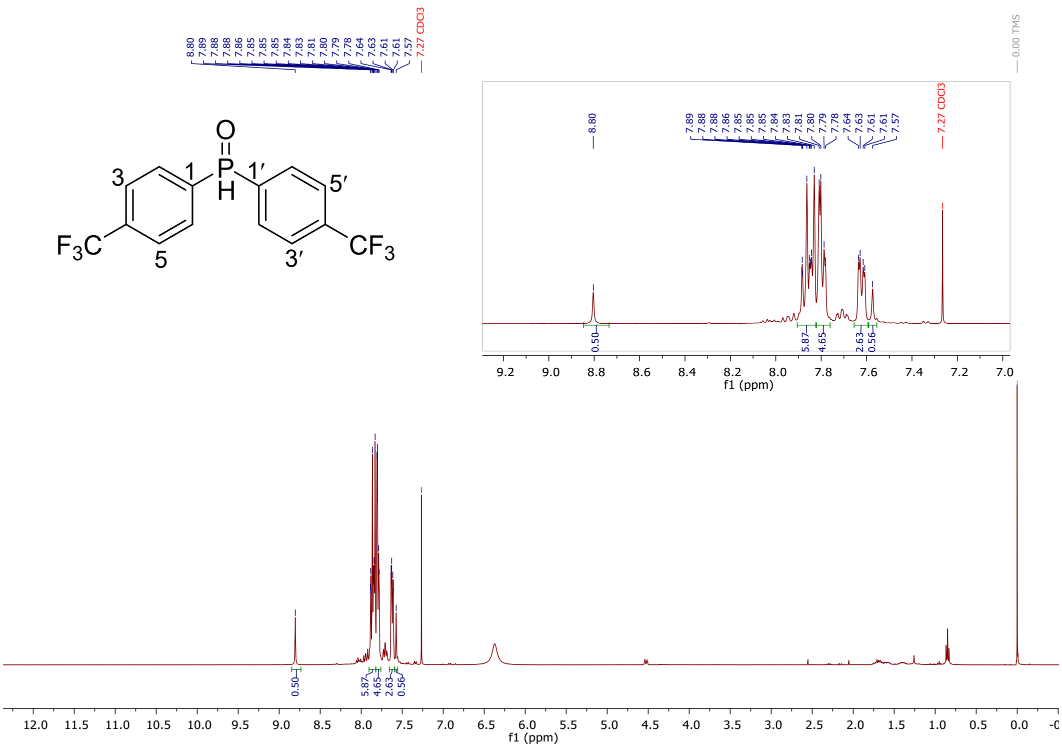

Figure S5. ^{1}H NMR spectrum of bis(*p*-(trifluoromethyl)phenyl)phosphine oxide (**11**) partially purified ($CDCl_3$, 400 MHz).

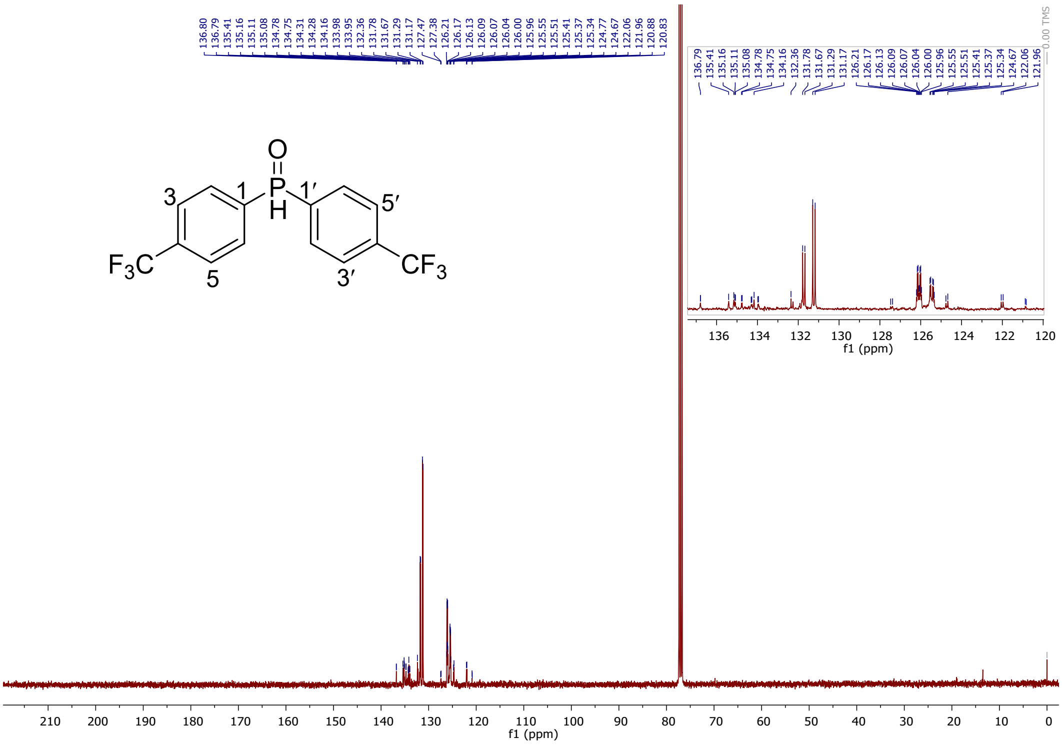

Figure S6. ^{13}C NMR spectrum of bis(*p*-(trifluoromethyl)phenyl)phosphine oxide (**11**) partially purified ($CDCl_3$, 101 MHz).

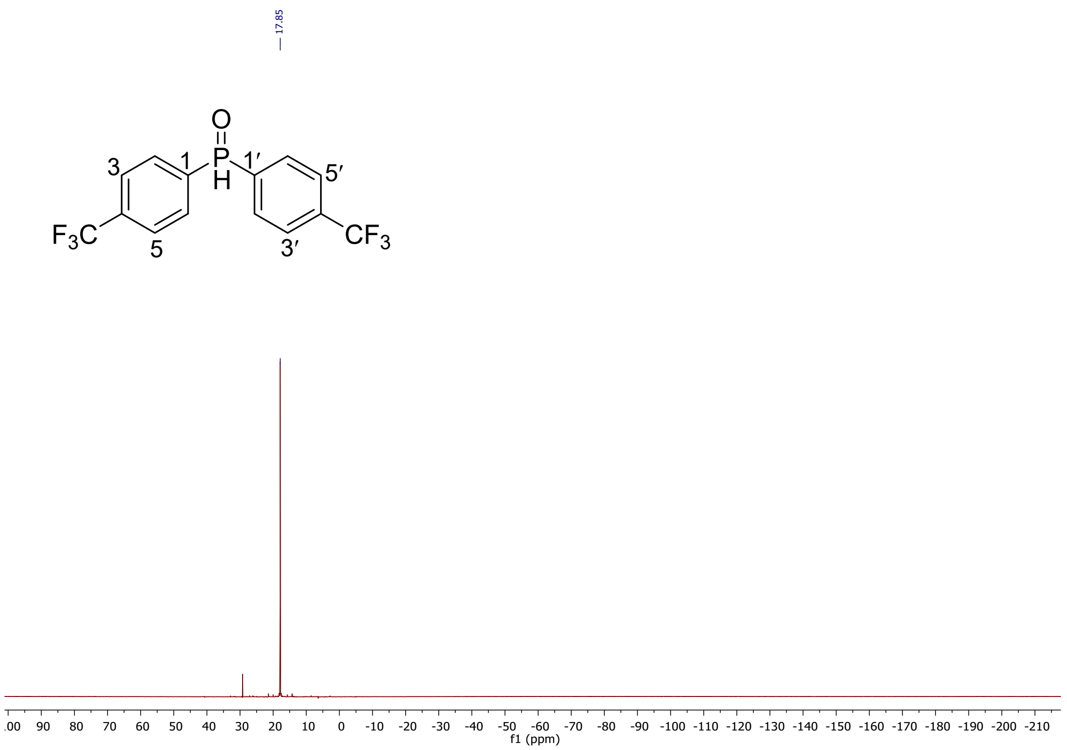

Figure S7. 3[1]P NMR spectrum of bis(*p*-(trifluoromethyl)phenyl)phosphine oxide (**11**) partially purified ($CDCl_3$, 162 MHz).

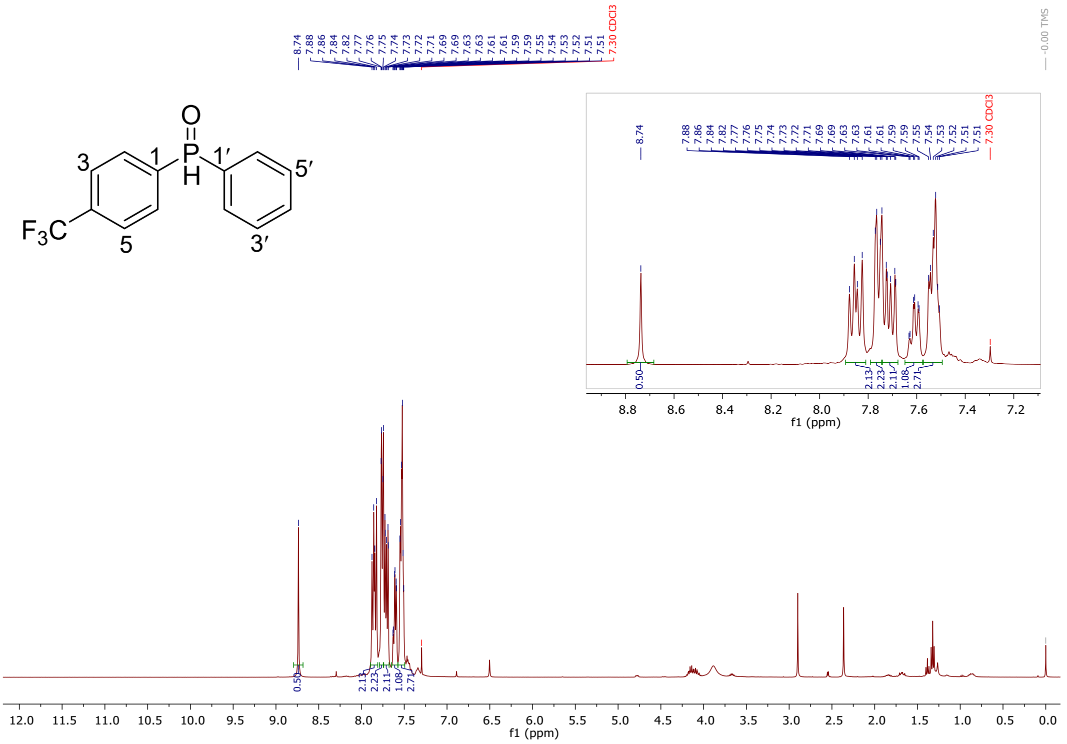

Figure S8. ^{1}H NMR spectrum of phenyl(4-(trifluoromethyl)phenyl)phosphine oxide (**12**) partially purified ($CDCl_3$, 400 MHz).

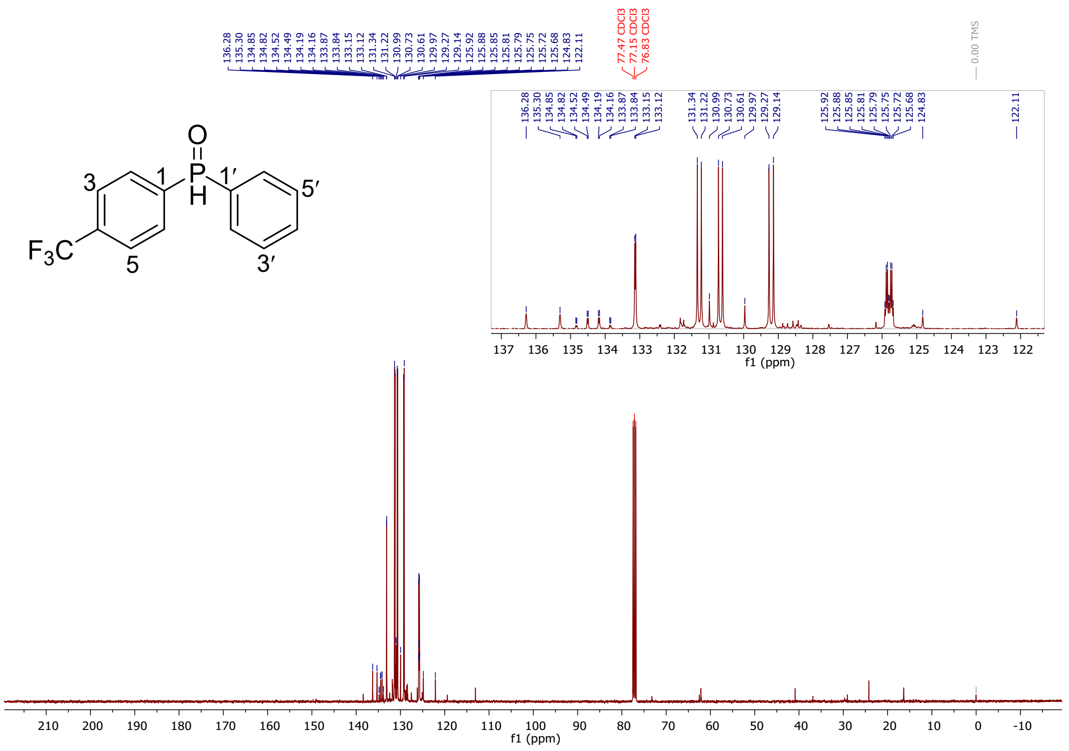

Figure S9. ^{13}C NMR spectrum of phenyl(4-(trifluoromethyl)phenyl)phosphine oxide (**12**) partially purified ($CDCl_3$, 101 MHz).

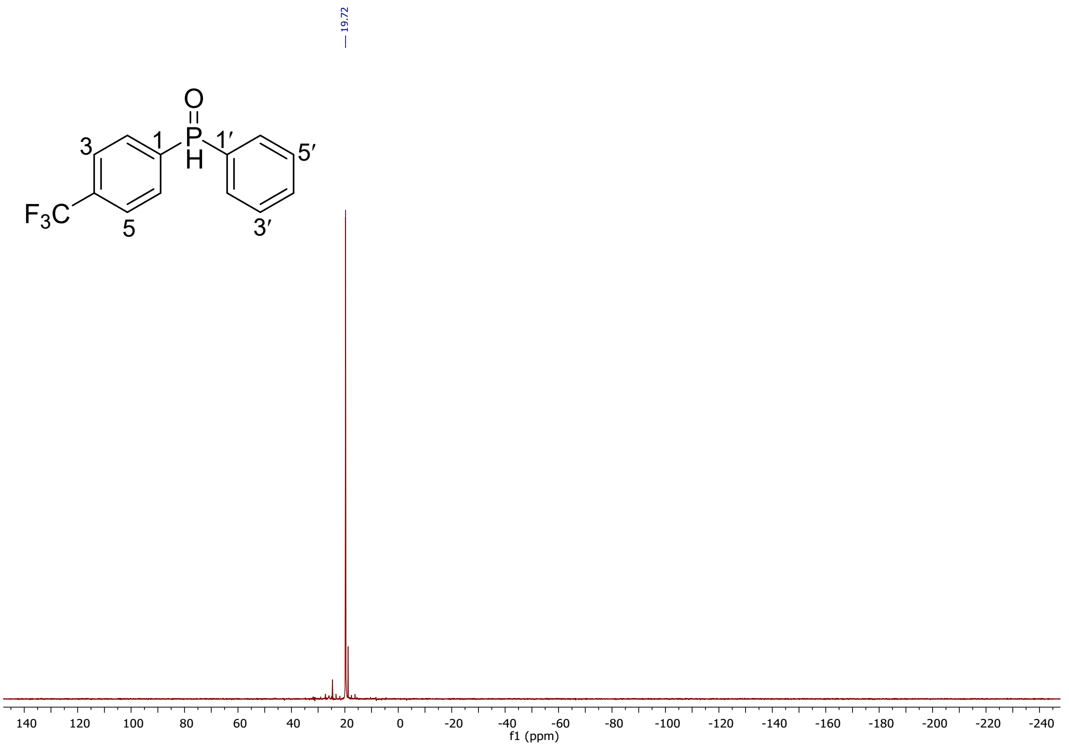

Figure S10. ^{31}P NMR spectrum of phenyl(4-(trifluoromethyl)phenyl)phosphine oxide (**12**) partially purified ($CDCl_3$, 162 MHz).

ESI 2.2 NMR Spectra of substituted benzaldehydes and arene intermediates

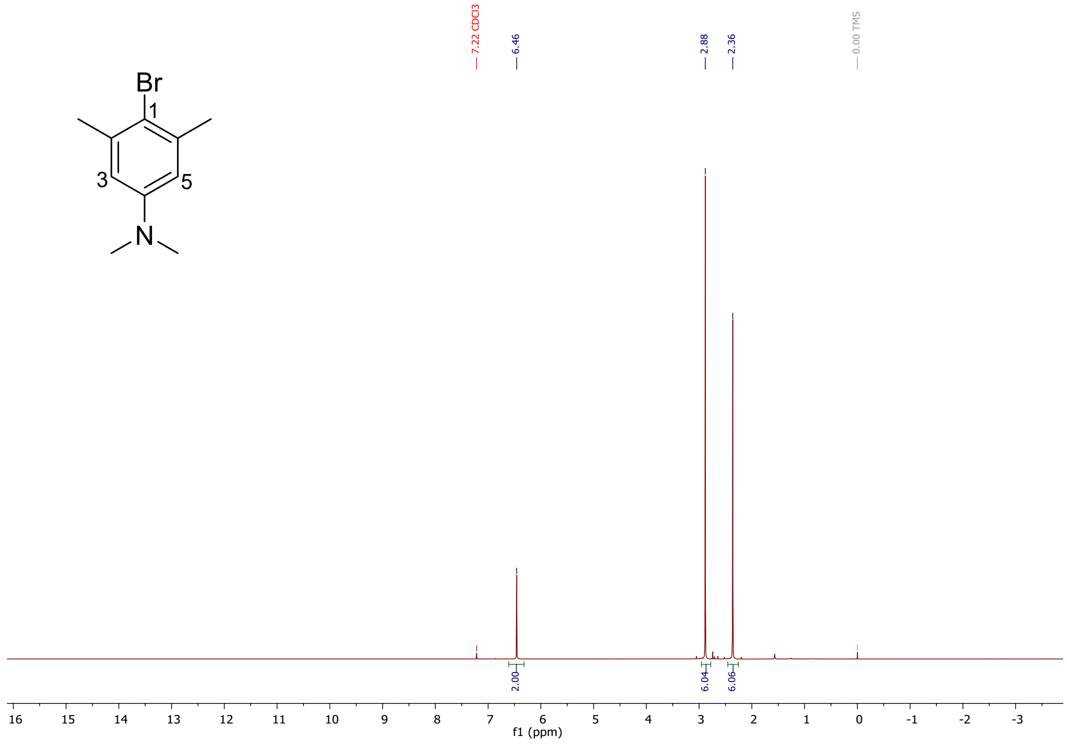

Figure S11. ^{1}H NMR spectrum of 4-bromo-*N*,*N*,3,5-tetramethylaniline (**13**) ($CDCl_3$, 400 MHz).

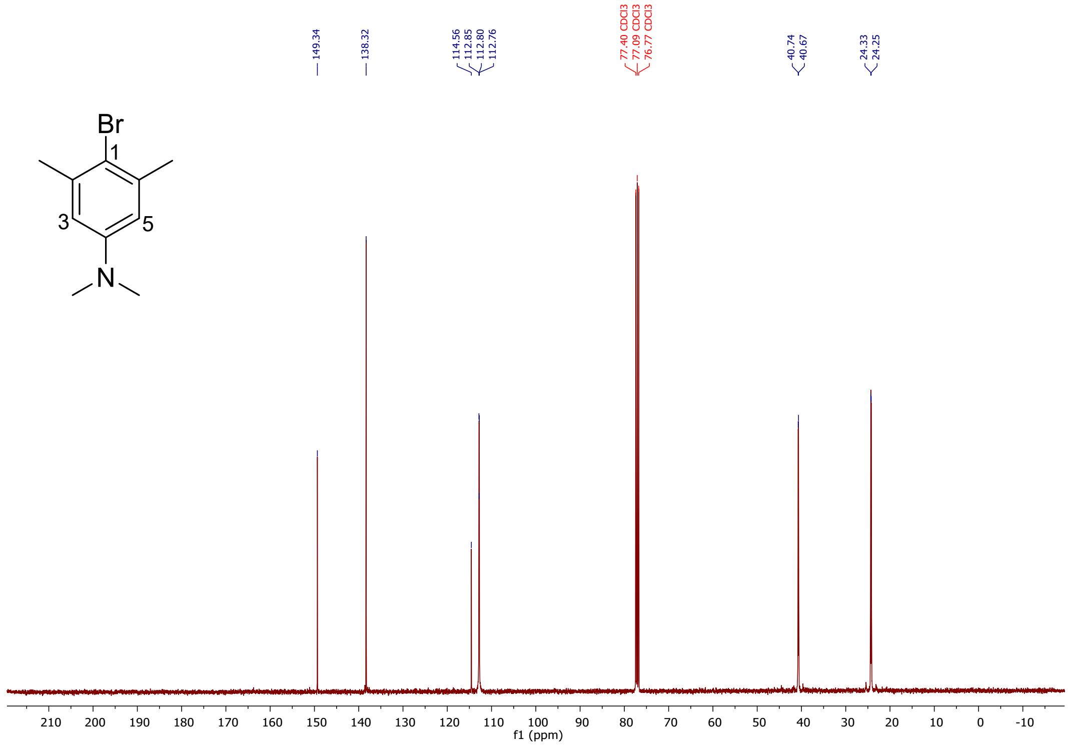

Figure S12. ^{13}C NMR spectrum of 4-bromo-*N*,*N*,3,5-tetramethylaniline (**13**) ($CDCl_3$, 101 MHz).

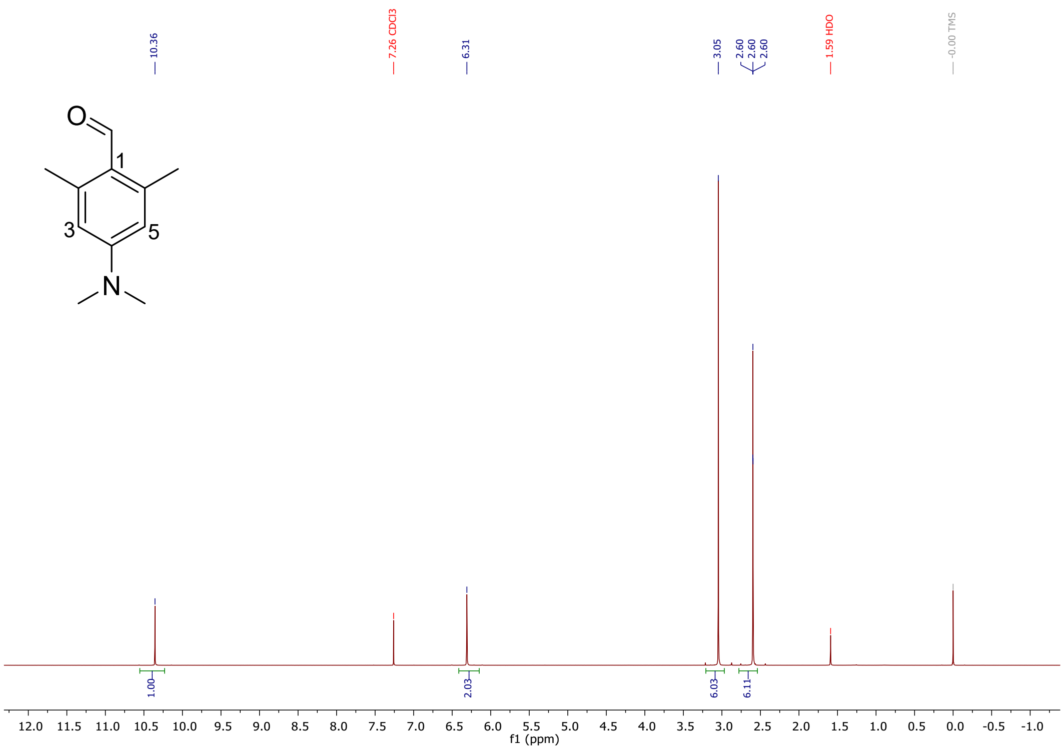

Figure S13. ^{1}H NMR spectrum of 4-(dimethylamino)-2,6-dimethylbenzaldehyde (**14**) ($CDCl_3$, 400 MHz).

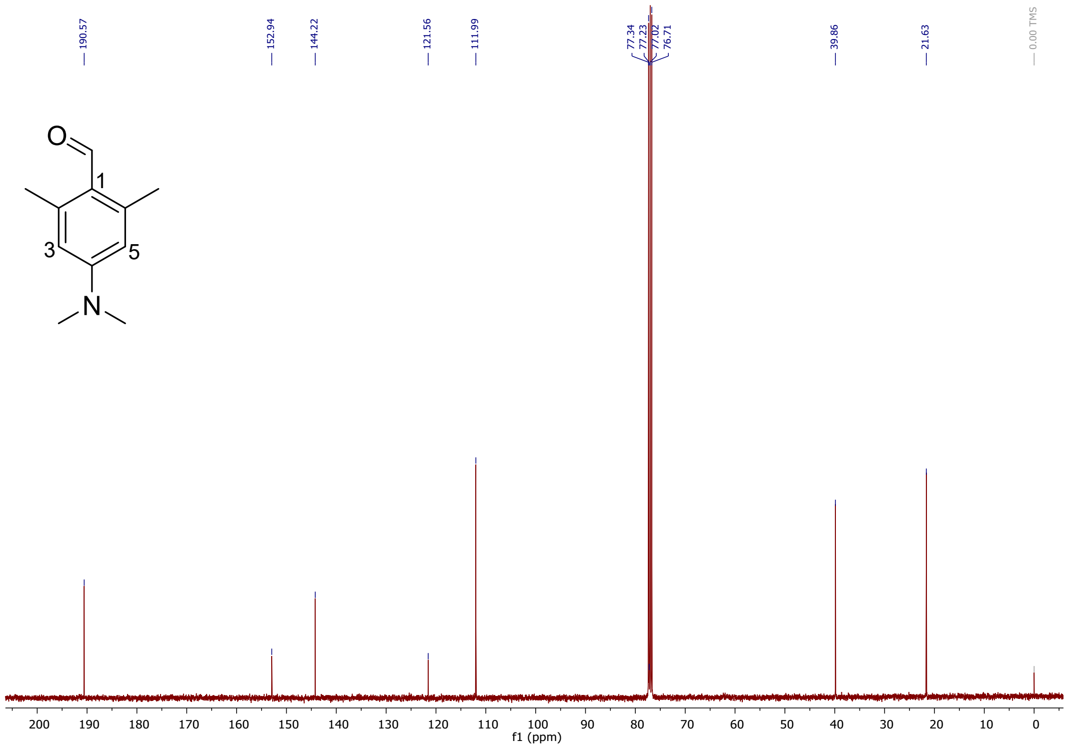

Figure S14. ^{13}C NMR spectrum of 4-(dimethylamino)-2,6-dimethylbenzaldehyde (**14**) ($CDCl_3$, 101 MHz).

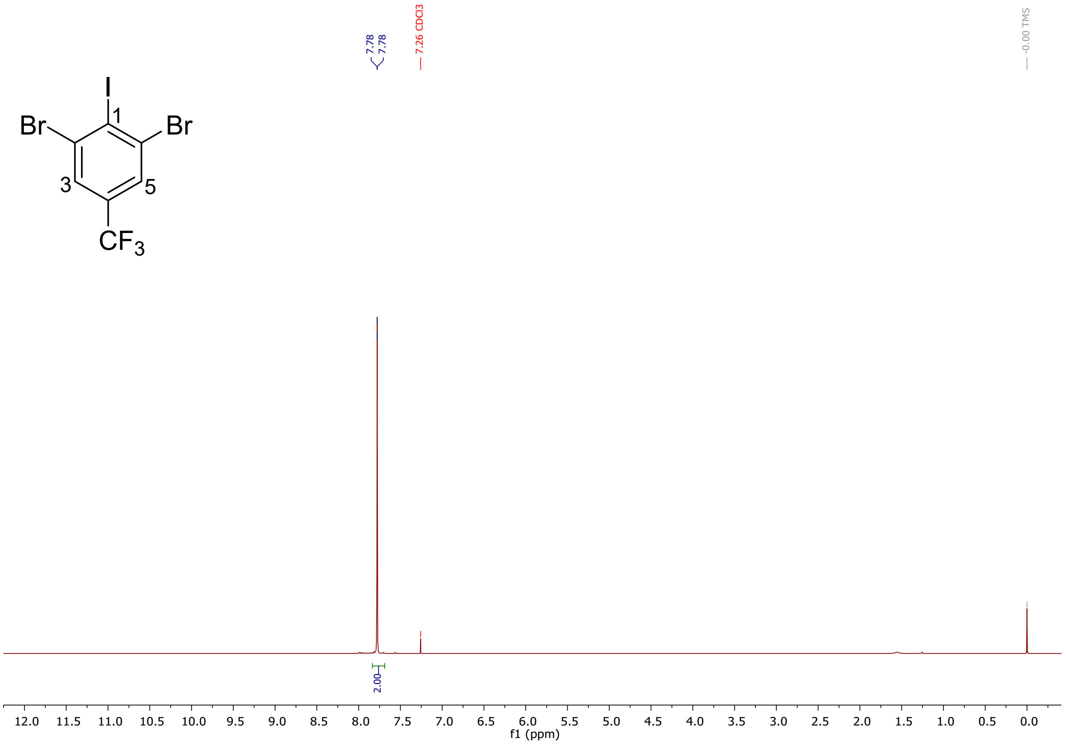

Figure S15. ^{1}H NMR spectrum of 1,3-dibromo-2-iodo-5-(trifluoromethyl)benzene (**15**) ($CDCl_3$, 400 MHz).

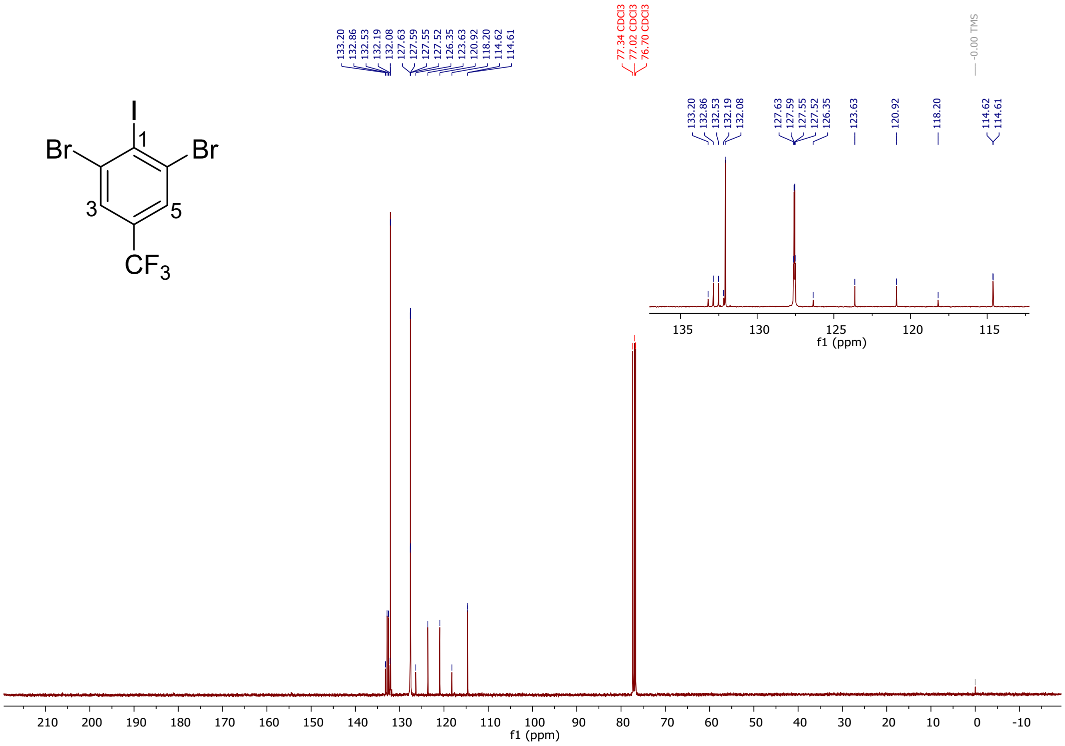

Figure S16. ^{13}C NMR spectrum of 1,3-dibromo-2-iodo-5-(trifluoromethyl)benzene (**15**) ($CDCl_3$, 101 MHz).

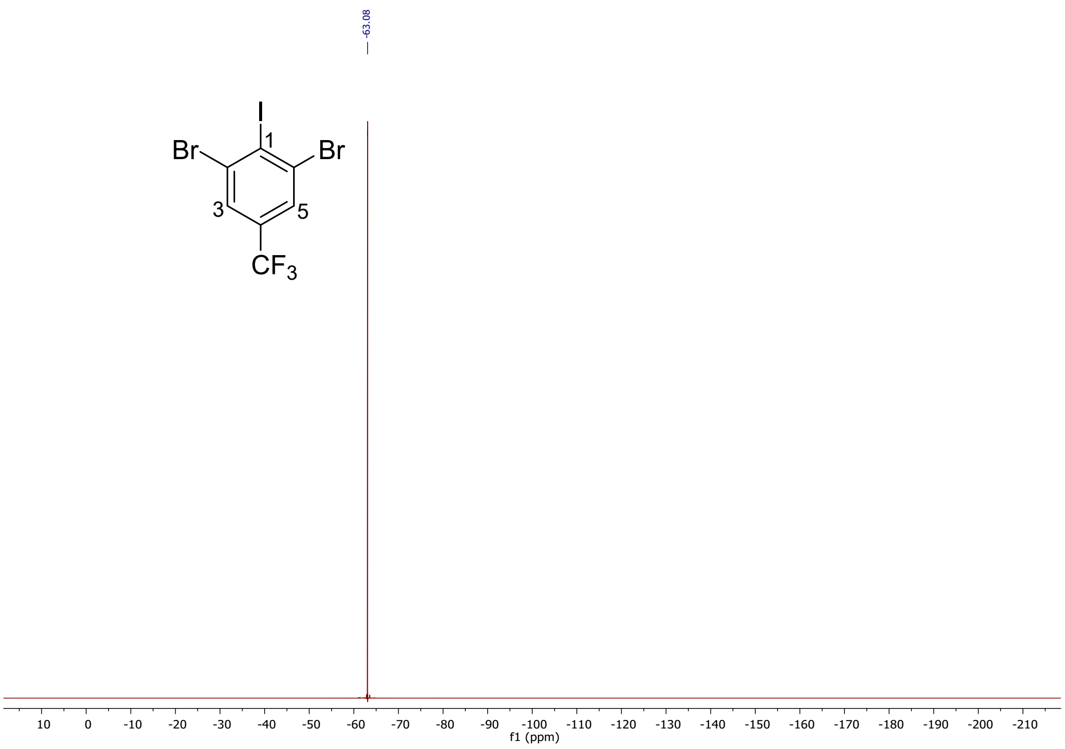

Figure S17. ^{19}F NMR spectrum of 1,3-dibromo-2-iodo-5-(trifluoromethyl)benzene (**15**) ($CDCl_3$, 376 MHz).

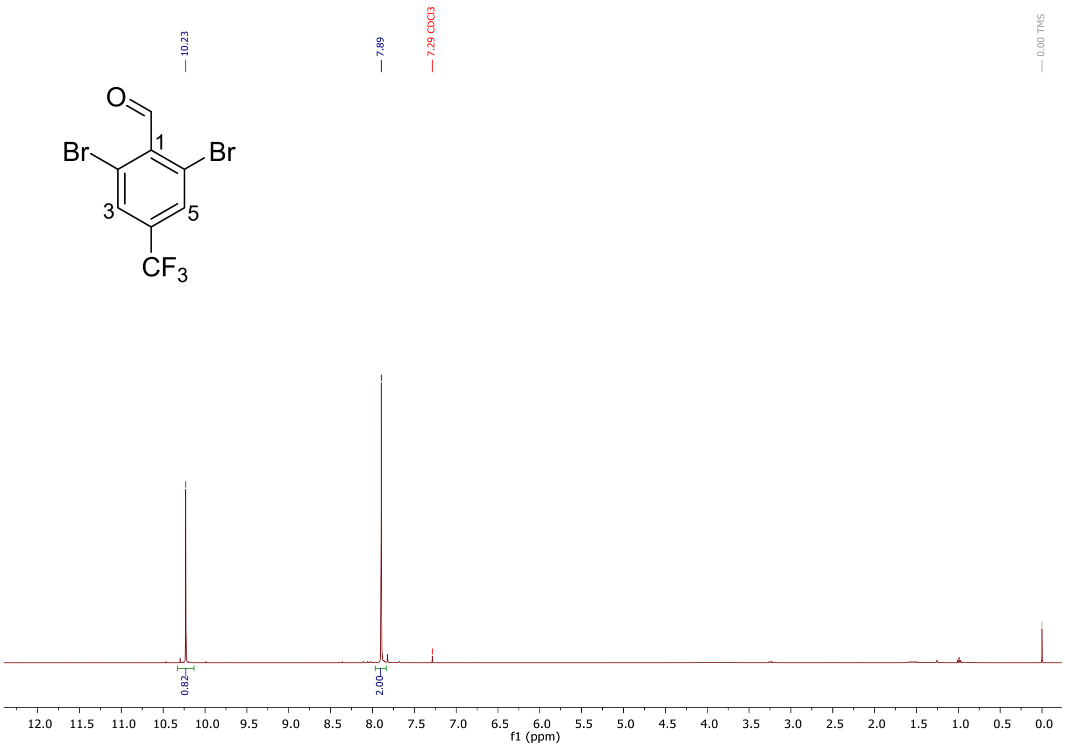

Figure S18. ^{1}H NMR spectrum of 2,6-dibromo-4-(trifluoromethyl)benzaldehyde (**16**) ($CDCl_3$, 400 MHz).

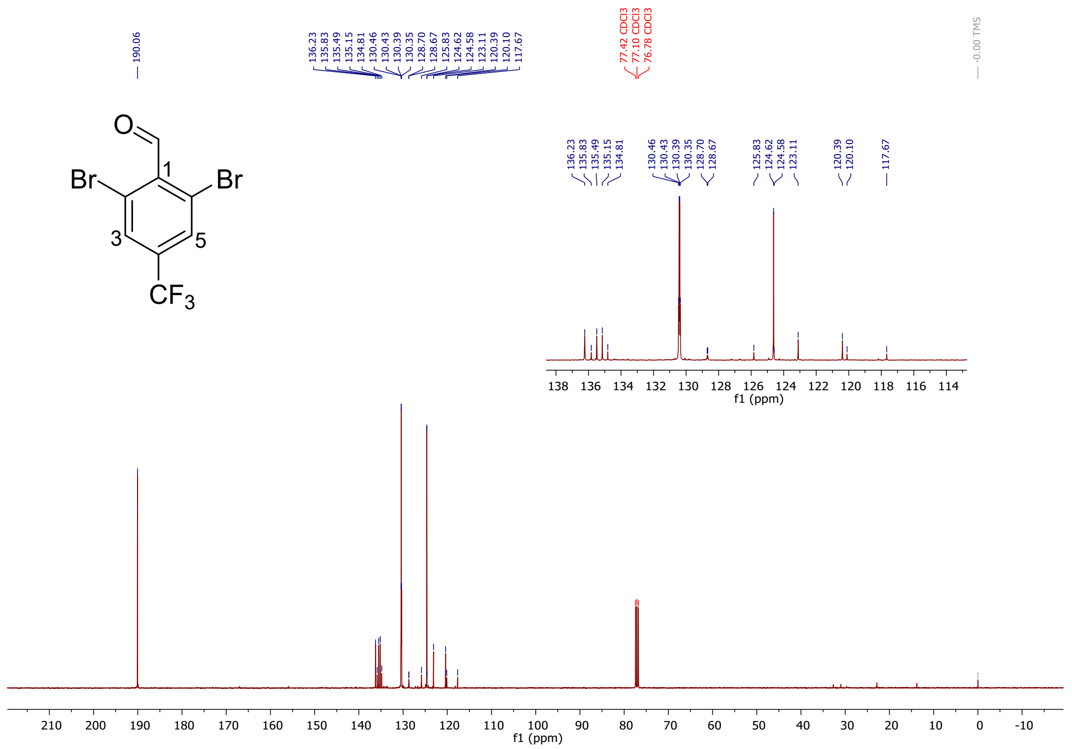

Figure S19. ^{13}C NMR spectrum of 2,6-dibromo-4-(trifluoromethyl)benzaldehyde (**16**) ($CDCl_3$, 101 MHz).

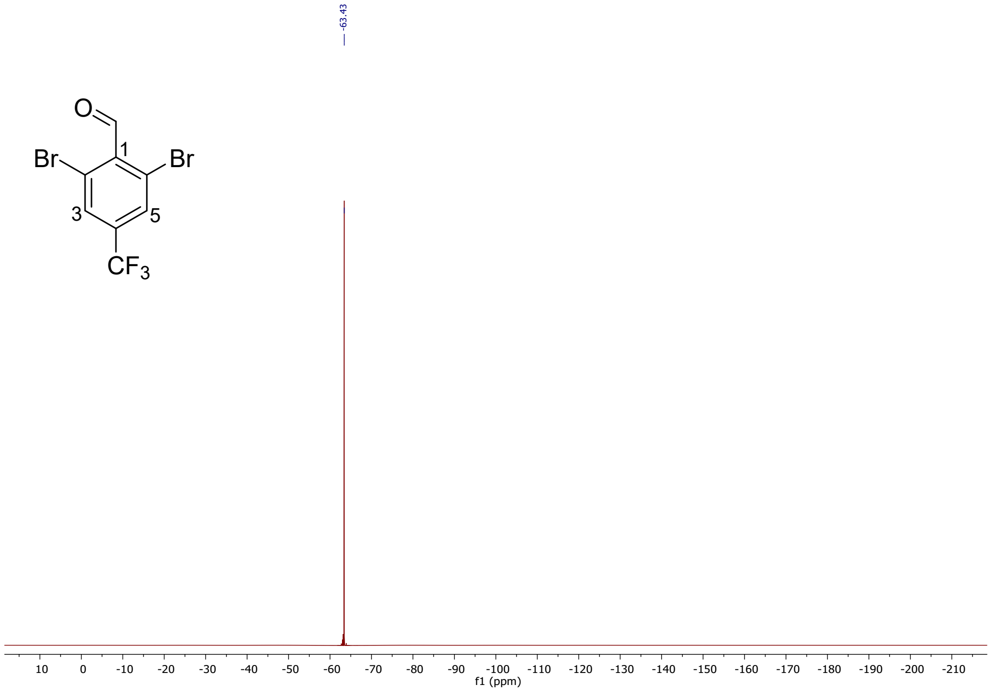

Figure S20. ^{19}F NMR spectrum of 2,6-dibromo-4-(trifluoromethyl)benzaldehyde (**16**) ($CDCl_3$, 376 MHz).

ESI 2.3 NMR spectra of α-hydroxyphosphine oxides

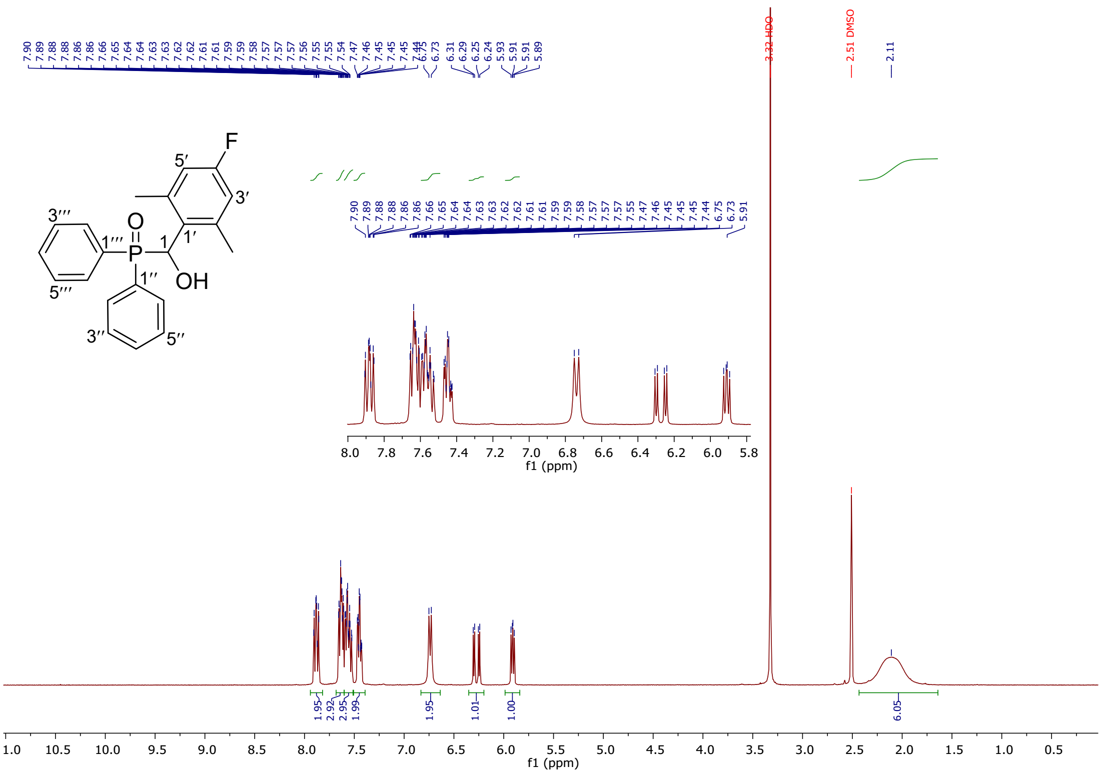

Figure S21. ^{1}H NMR spectrum of ((4-fluoro-2,6-dimethylphenyl)(hydroxy)methyl)diphenylphosphine oxide (**17**) (DMSO-d_6 400 MHz).

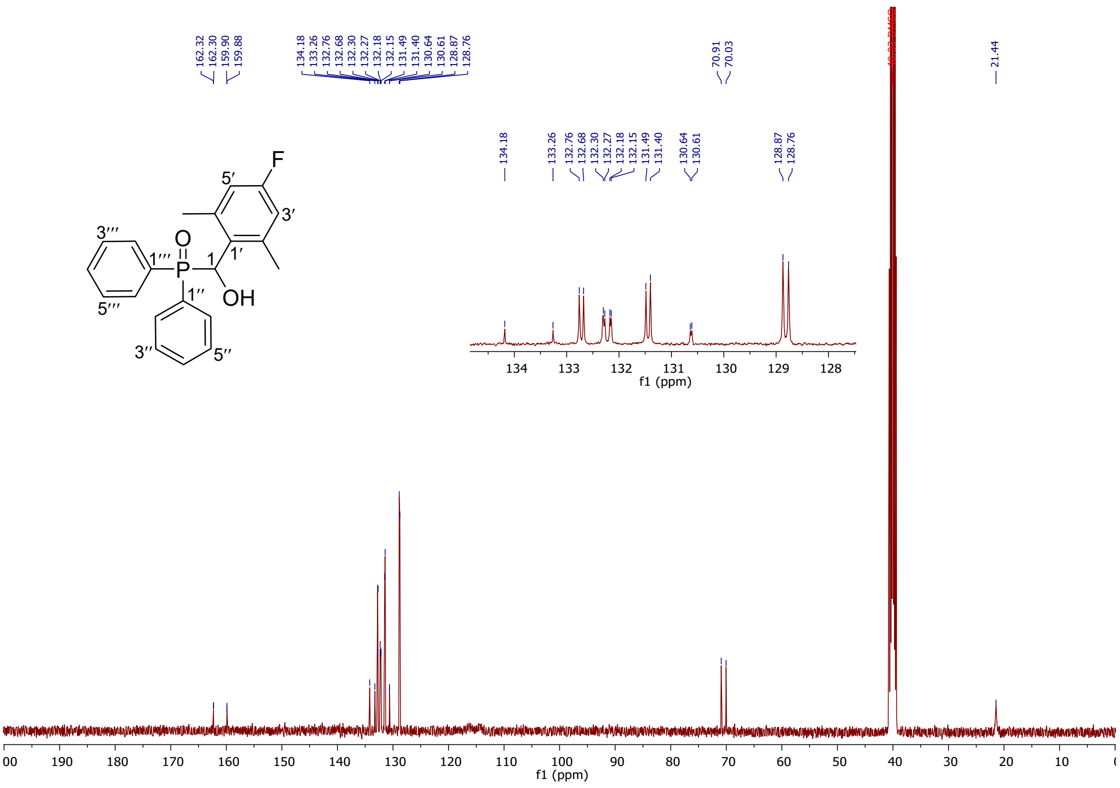

Figure S22. ^{13}C NMR spectrum of ((4-fluoro-2,6-dimethylphenyl)(hydroxy)methyl)diphenylphosphine oxide (**17**) (DMSO-d_6 101 MHz).

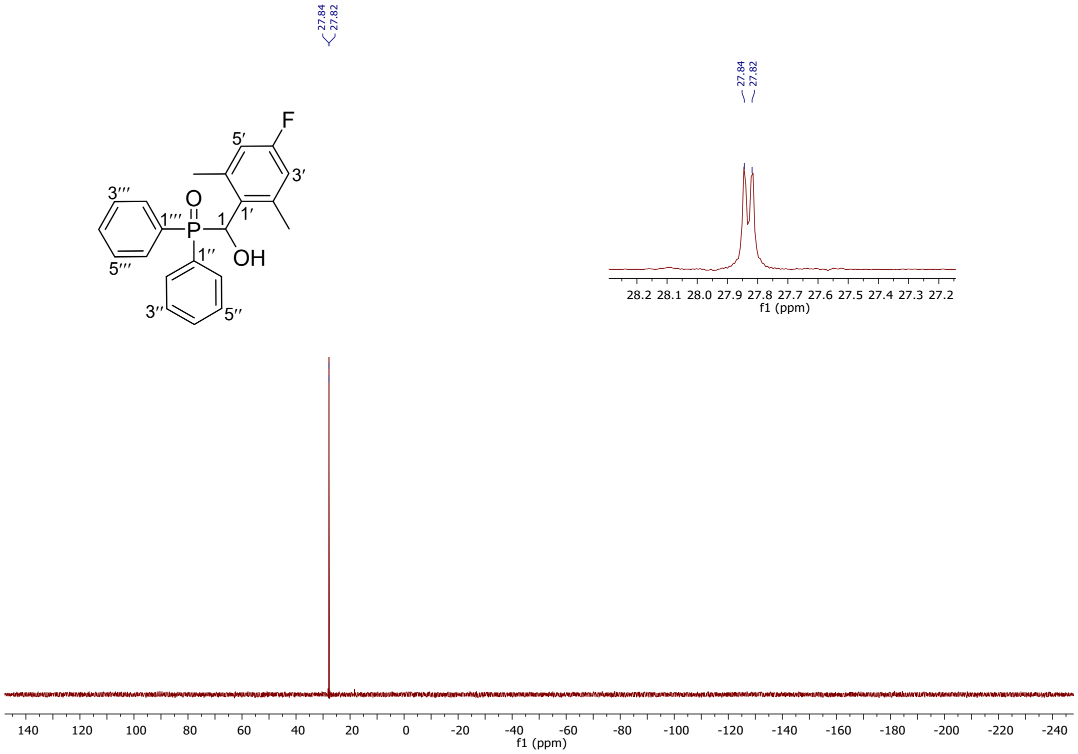

Figure S23. ^{31}P NMR spectrum of ((4-fluoro-2,6-dimethylphenyl)(hydroxy)methyl)diphenylphosphine oxide (**17**) (DMSO-d_6 162 MHz).

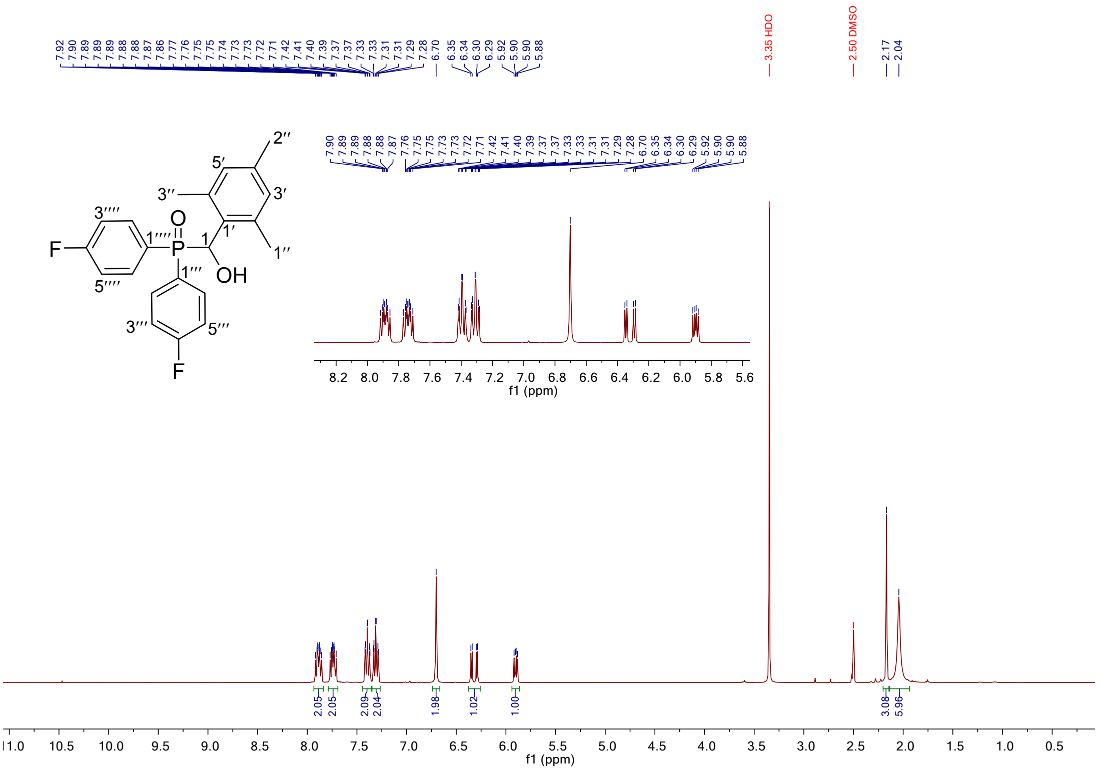

Figure S24. ^{1}H NMR spectrum of bis(4-fluorophenyl)(hydroxy(mesityl)methyl)phosphine oxide (**18**) (DMSO-d_6 400 MHz).

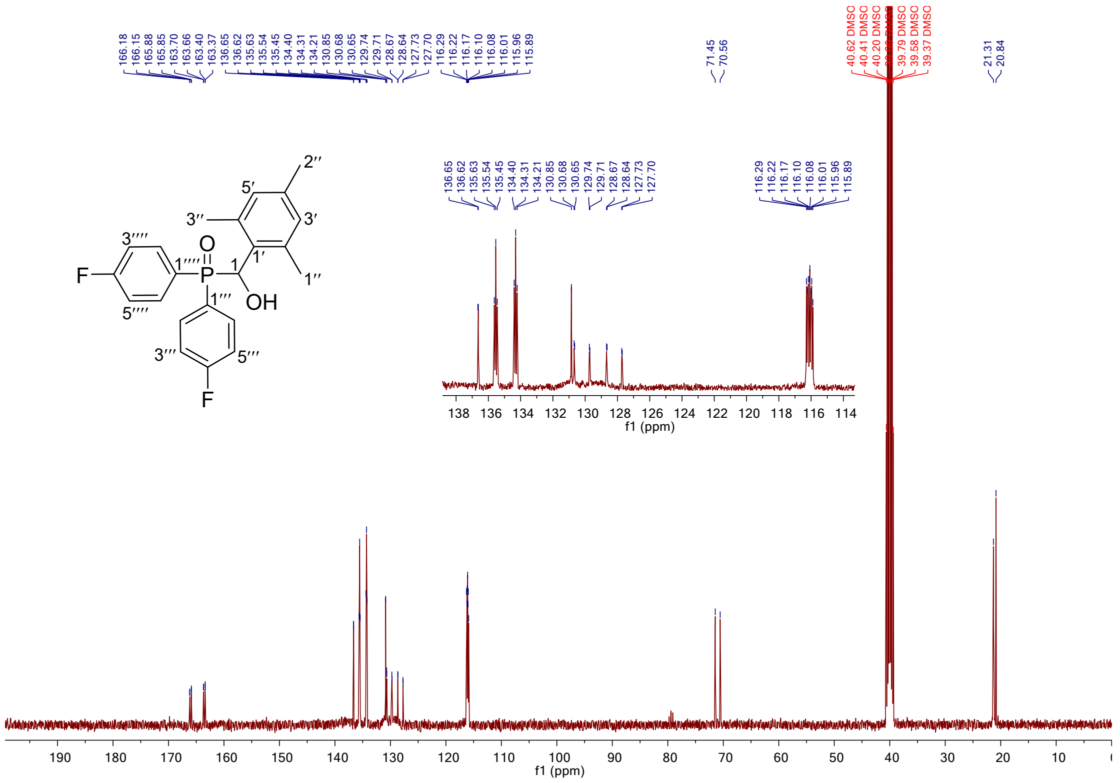

Figure S25. ^{13}C NMR spectrum of bis(4-fluorophenyl)(hydroxy(mesityl)methyl)phosphine oxide (**18**) (DMSO-d_6 101 MHz).

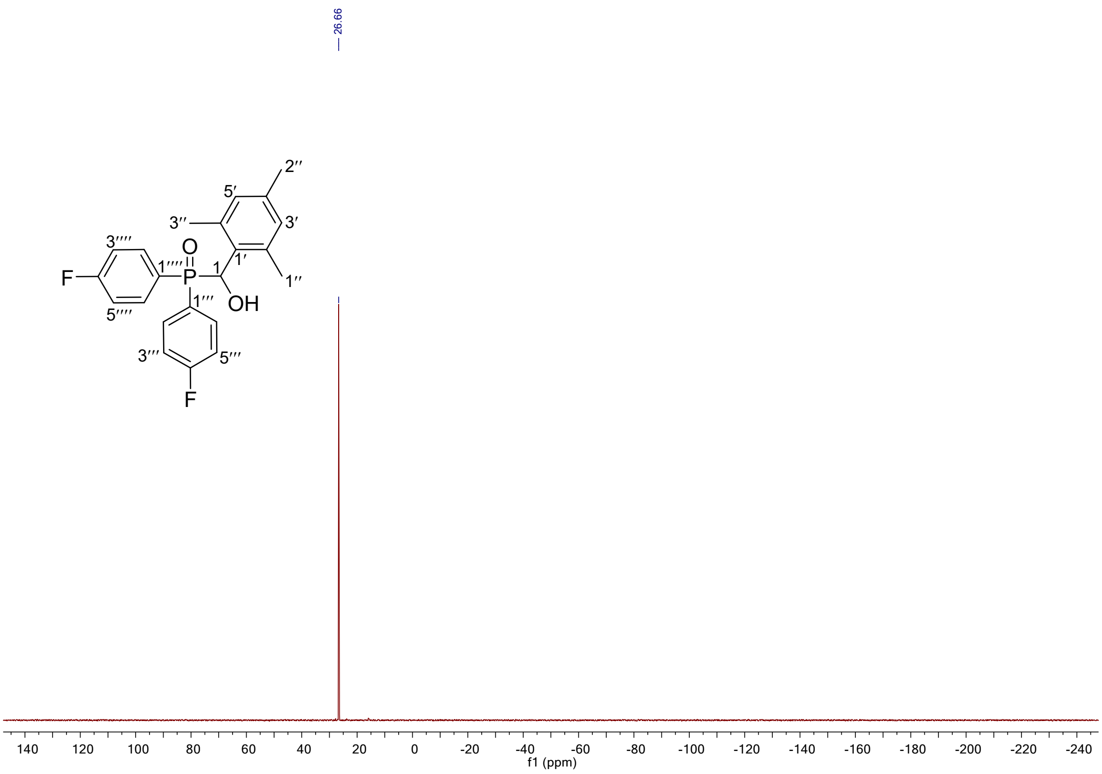

Figure S26. ^{31}P NMR spectrum of bis(4-fluorophenyl)(hydroxy(mesityl)methyl)phosphine oxide (**18**) (DMSO-d_6 162 MHz).

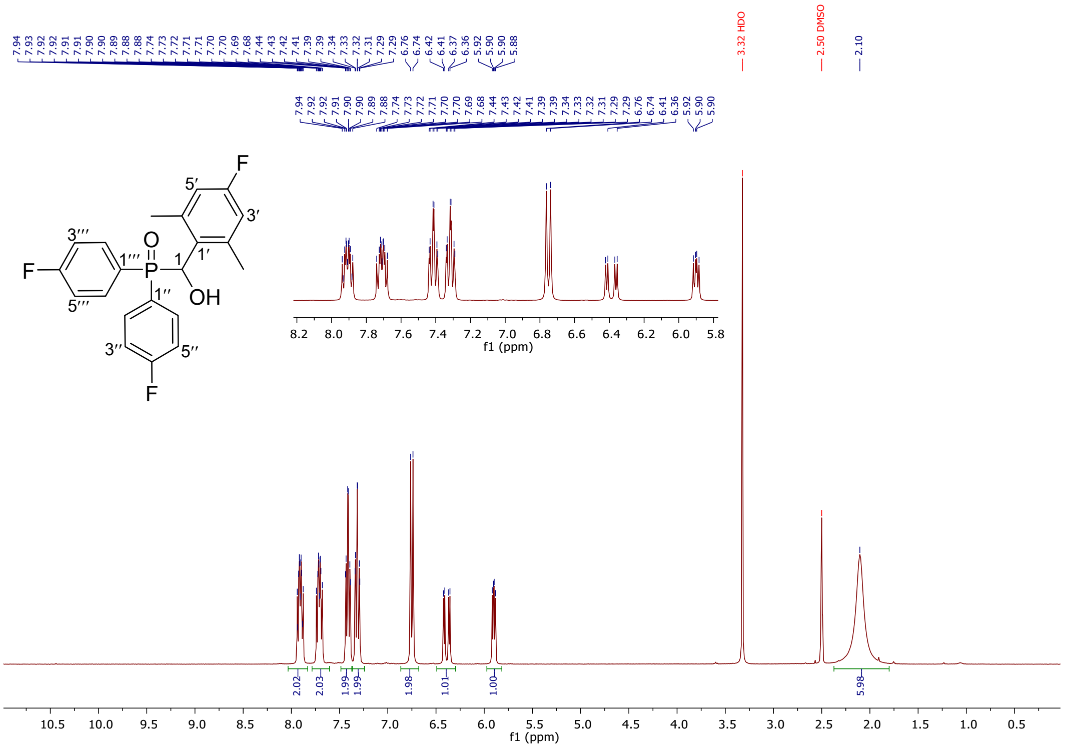

Figure S27. ^{1}H NMR spectrum of ((4-fluoro-2,6-dimethylphenyl)(hydroxy)methyl)bis(4-fluorophenyl)phosphine oxide (**19**) (DMSO-d_6 400 MHz).

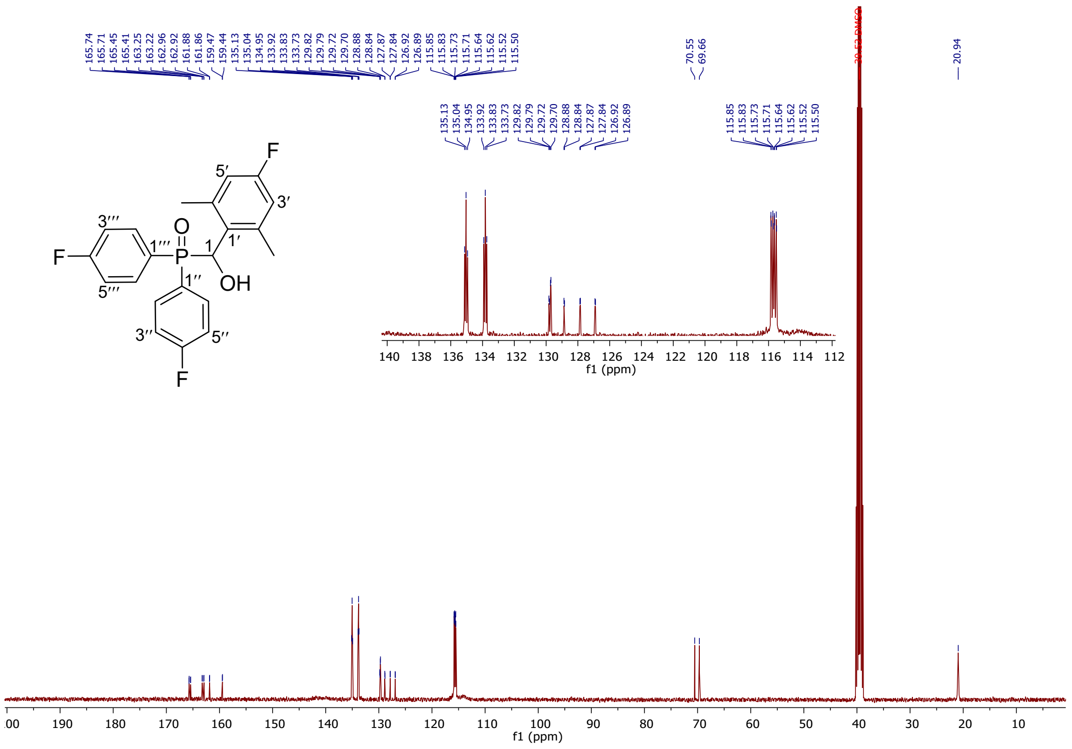

Figure S28. ^{13}C NMR spectrum of ((4-fluoro-2,6-dimethylphenyl)(hydroxy)methyl)bis(4-fluorophenyl)phosphine oxide (**19**) (DMSO-d_6 101 MHz).

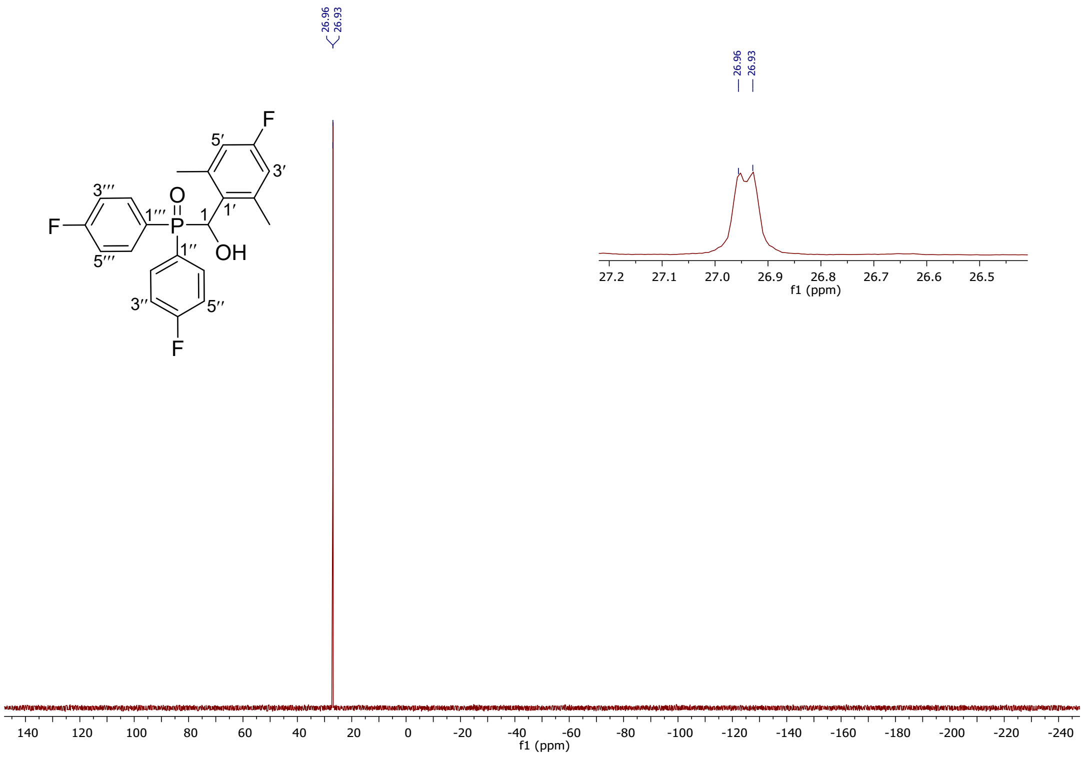

Figure S29. ^{31}P NMR spectrum of ((4-fluoro-2,6-dimethylphenyl)(hydroxy)methyl)bis(4-fluorophenyl)phosphine oxide (**19**) (DMSO-d_6 162 MHz).

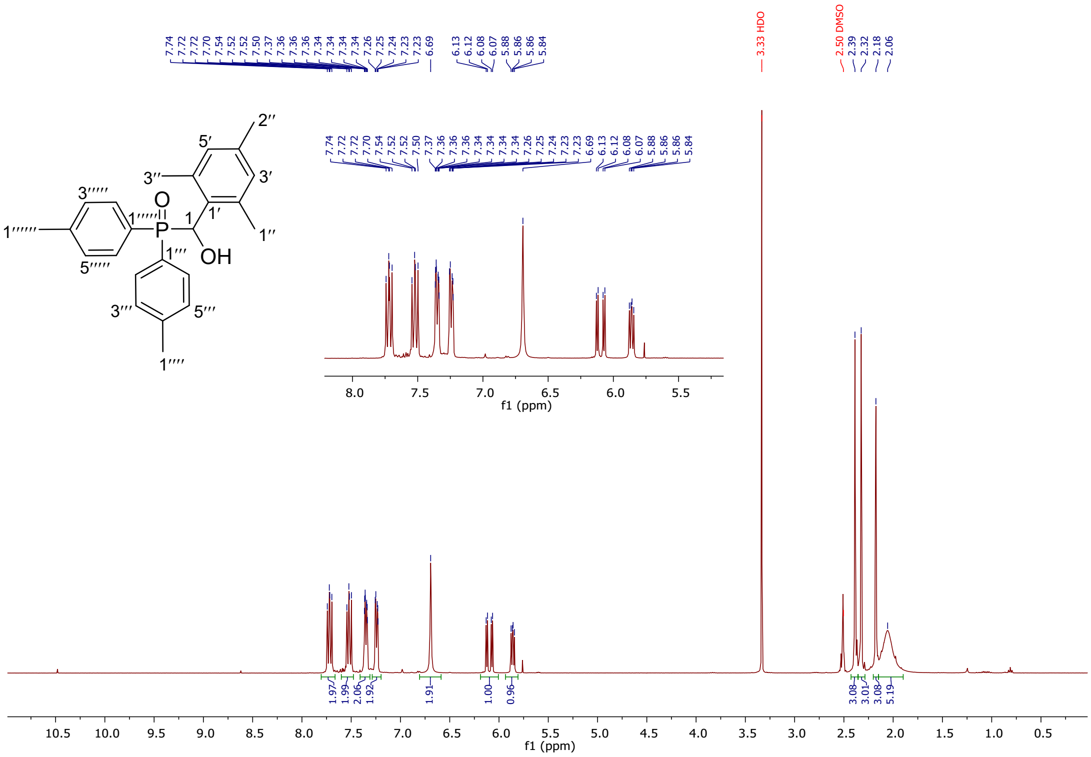

Figure S30. ^{1}H NMR spectrum of (hydroxy(mesityl)methyl)di-p-tolylphosphine oxide (**20**) (DMSO-d_6 400 MHz).

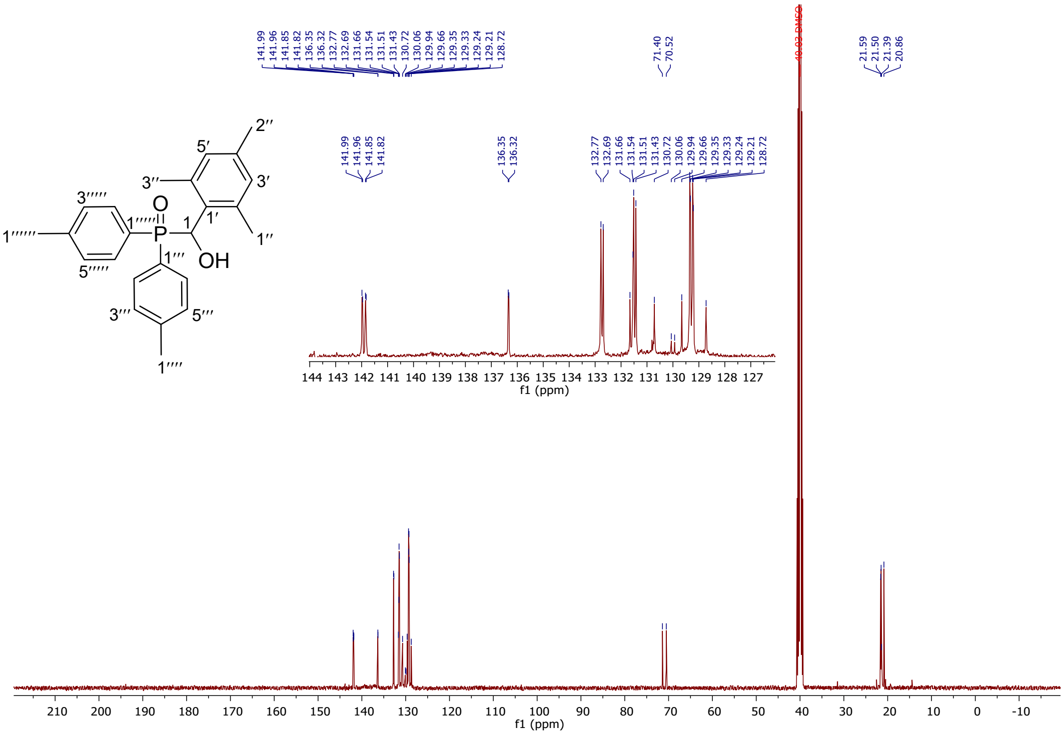

Figure S31. ^{13}C NMR spectrum of (hydroxy(mesityl)methyl)di-p-tolylphosphine oxide (**20**) (DMSO-d_6 101 MHz).

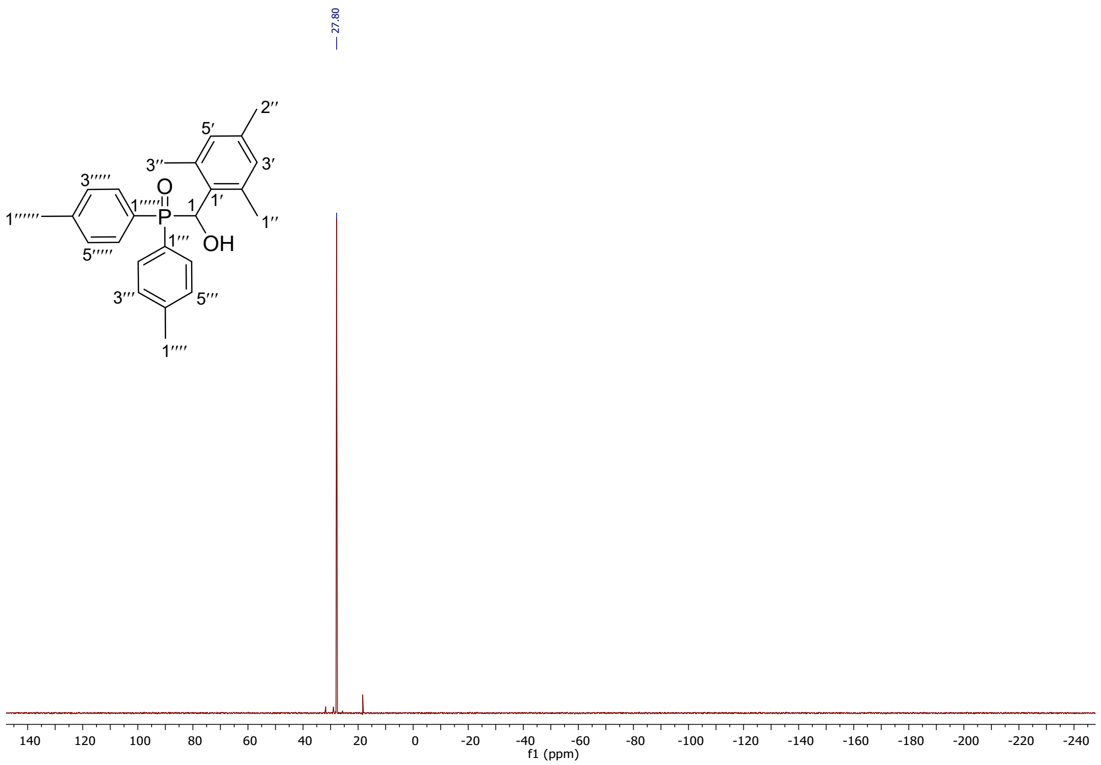

Figure S32. ^{31}P NMR spectrum of (hydroxy(mesityl)methyl)di-p-tolylphosphine oxide (**20**) (DMSO-d_6 162 MHz).

ESI 2.4 NMR spectra of monoacylphosphine oxides

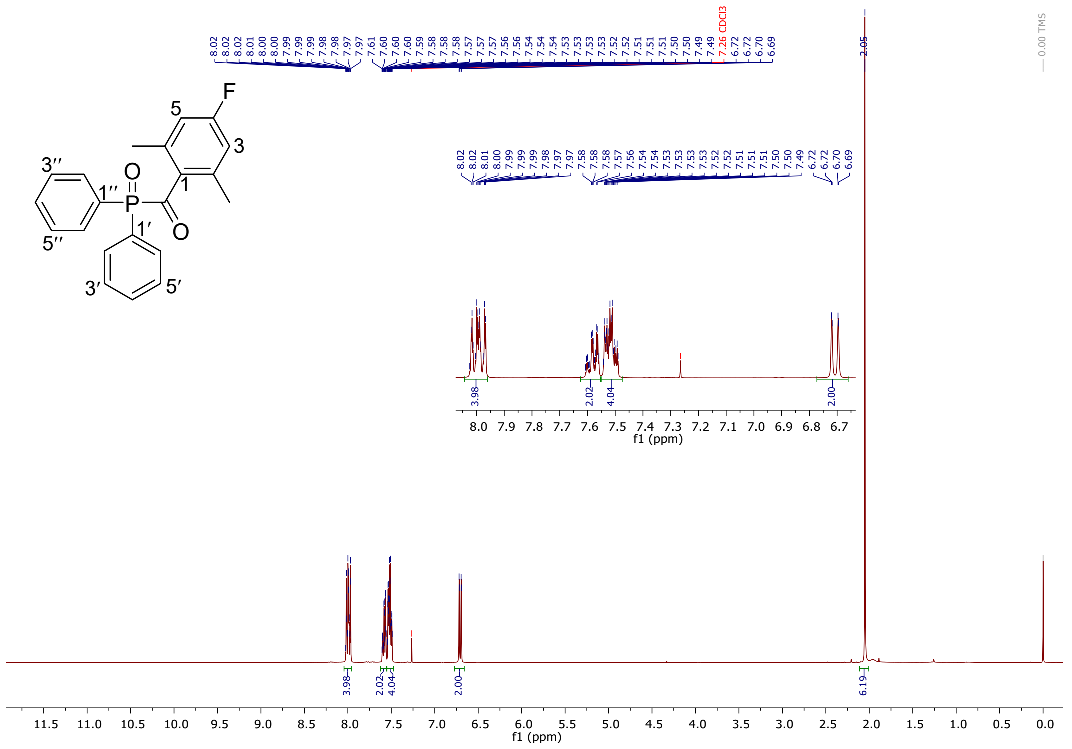

Figure S33. ^{1}H NMR spectrum of (diphenylphosphoryl)(4-fluoro-2,6-dimethylphenyl)methanone (**1**) ($CDCl_3$, 400 MHz).

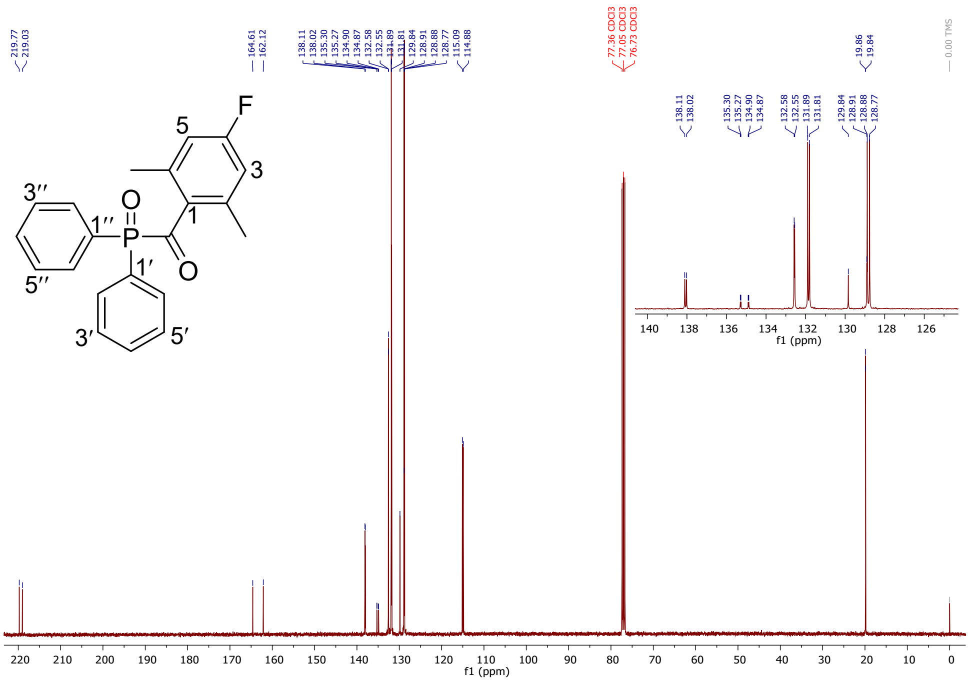

Figure S34. ^{13}C NMR spectrum of (diphenylphosphoryl)(4-fluoro-2,6-dimethylphenyl)methanone (**1**) ($CDCl_3$, 101 MHz).

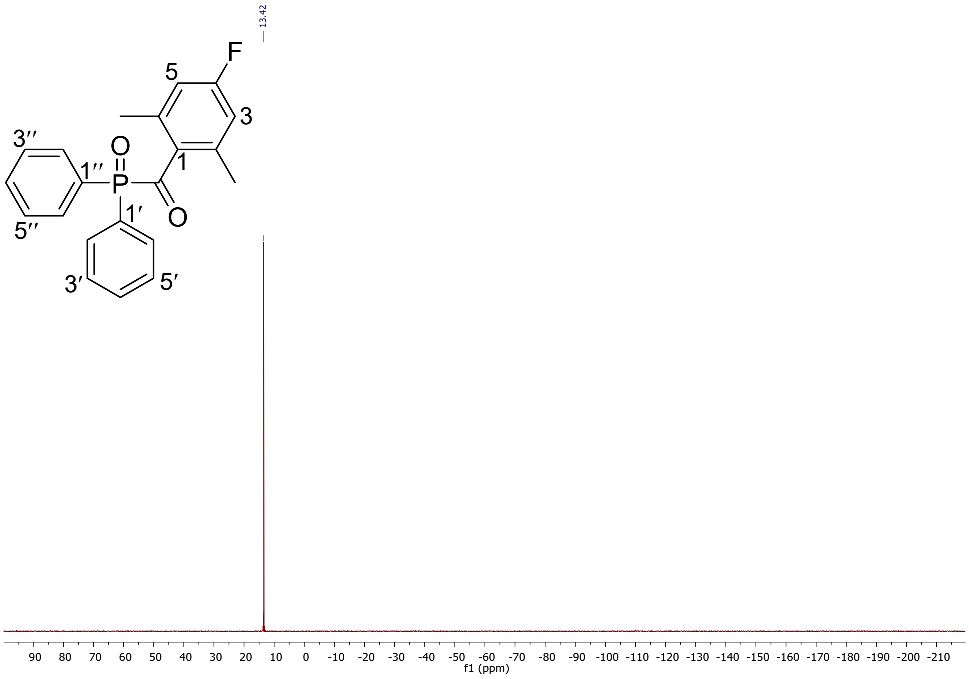

Figure S35. ^{19}F NMR spectrum of (diphenylphosphoryl)(4-fluoro-2,6-dimethylphenyl)methanone (**1**) ($CDCl_3$, 162 MHz).

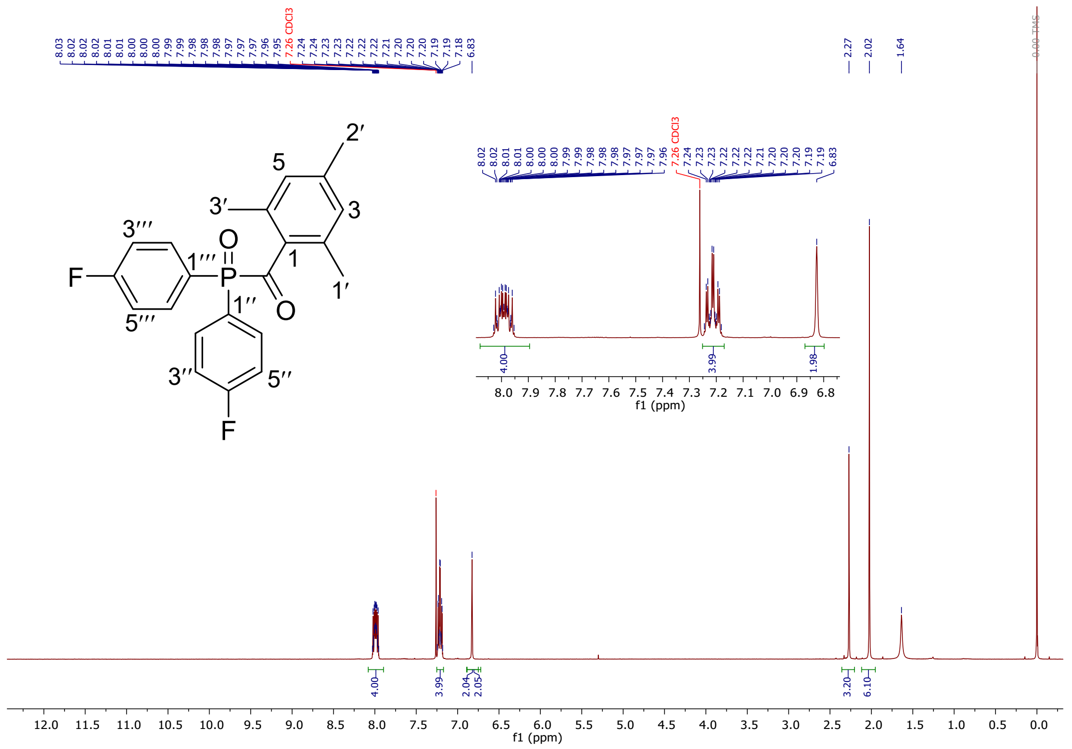

Figure S36. ^{1}H NMR spectrum of (bis(4-fluorophenyl)phosphoryl)(mesityl)methanone (**2**) ($CDCl_3$, 400 MHz).

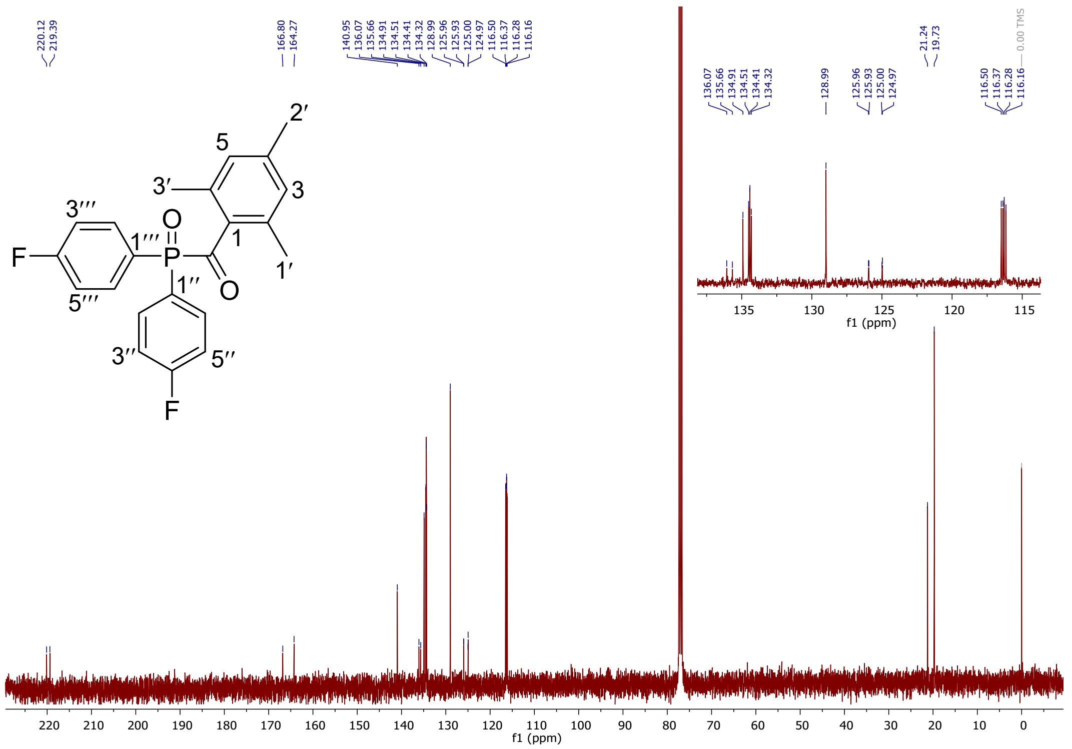

Figure S37. ^{13}C NMR spectrum of (bis(4-fluorophenyl)phosphoryl)(mesityl)methanone (**2**) ($CDCl_3$, 101 MHz).

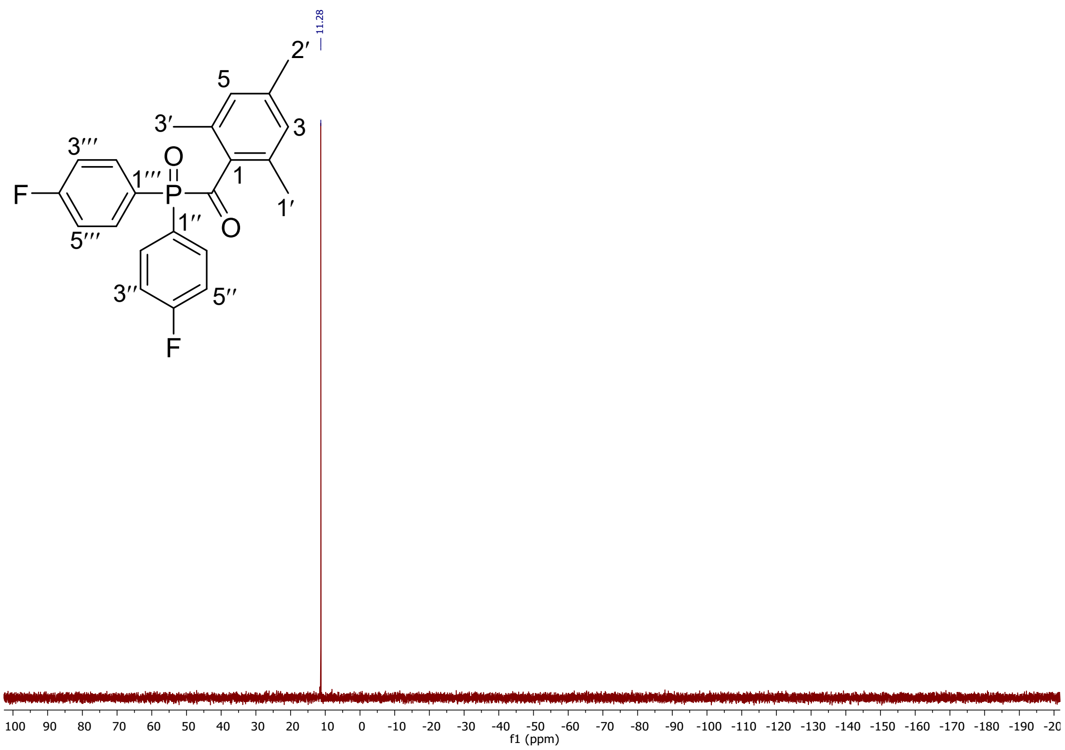

Figure S38. ^{31}P NMR spectrum of (bis(4-fluorophenyl)phosphoryl)(mesityl)methanone (**2**) ($CDCl_3$, 162 MHz).

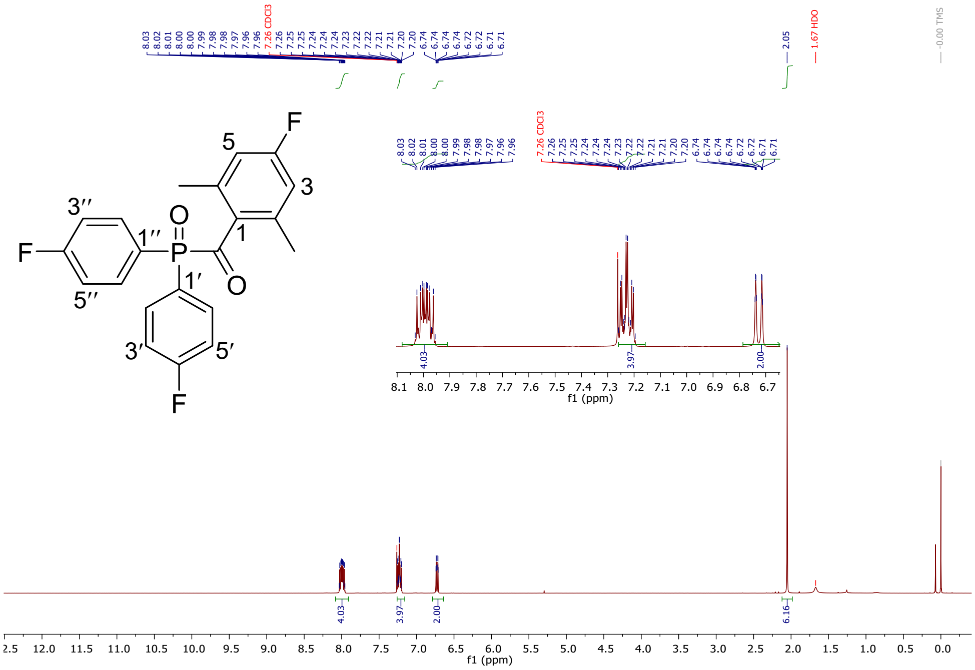

Figure S39. ^{1}H NMR spectrum of (bis(4-fluorophenyl)phosphoryl)(4-fluoro-2,6-dimethylphenyl)methanone (**3**) ($CDCl_3$, 400 MHz).

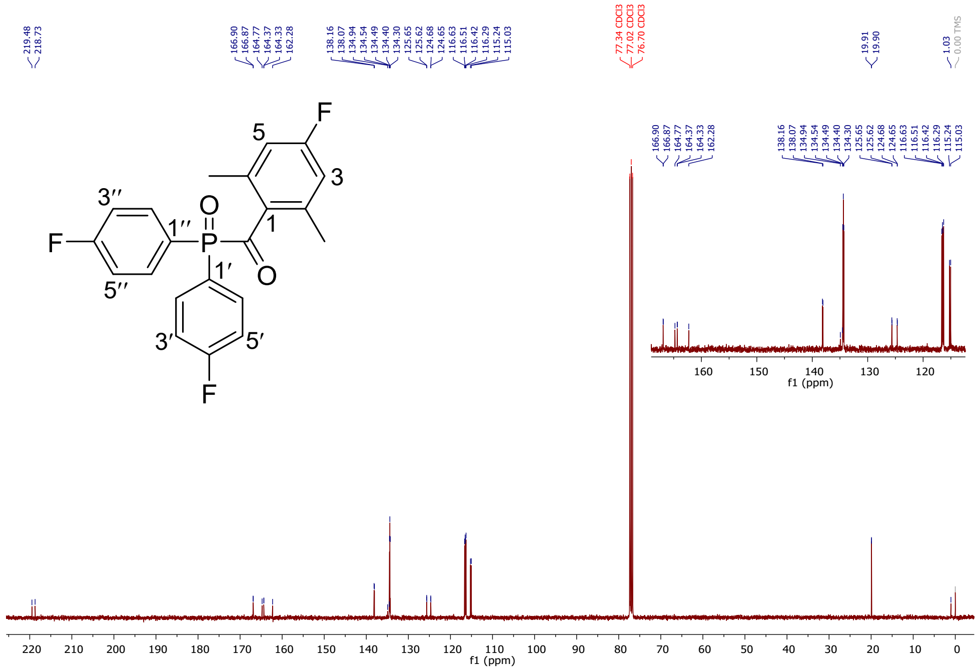

Figure S40. ^{13}C NMR spectrum of (bis(4-fluorophenyl)phosphoryl)(4-fluoro-2,6-dimethylphenyl)methanone (**3**) ($CDCl_3$, 101 MHz).

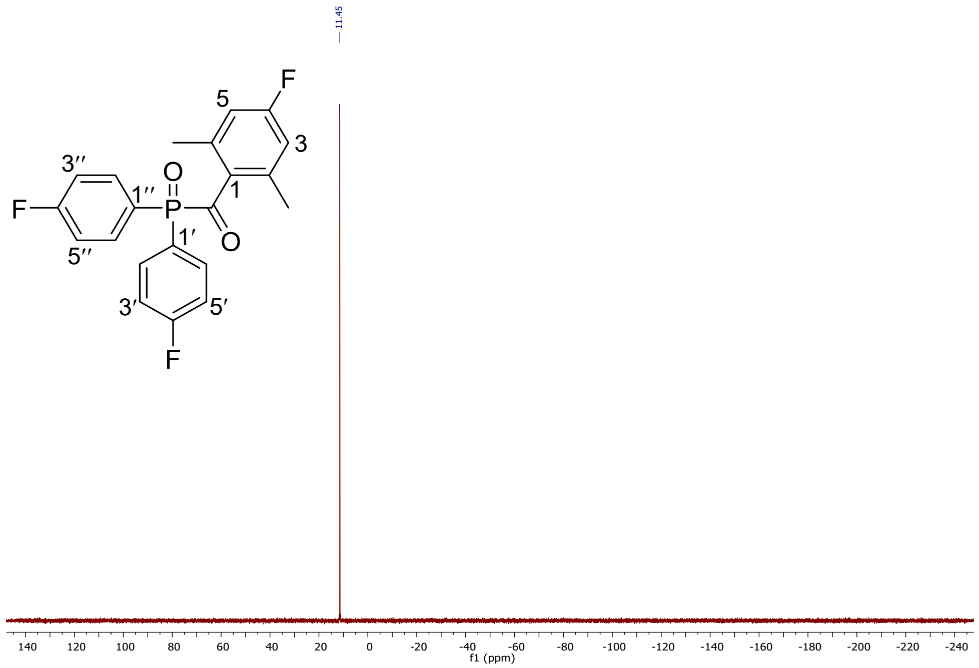

Figure S41. ^{31}P NMR spectrum of (bis(4-fluorophenyl)phosphoryl)(4-fluoro-2,6-dimethylphenyl)methanone (**3**) ($CDCl_3$, 162 MHz).

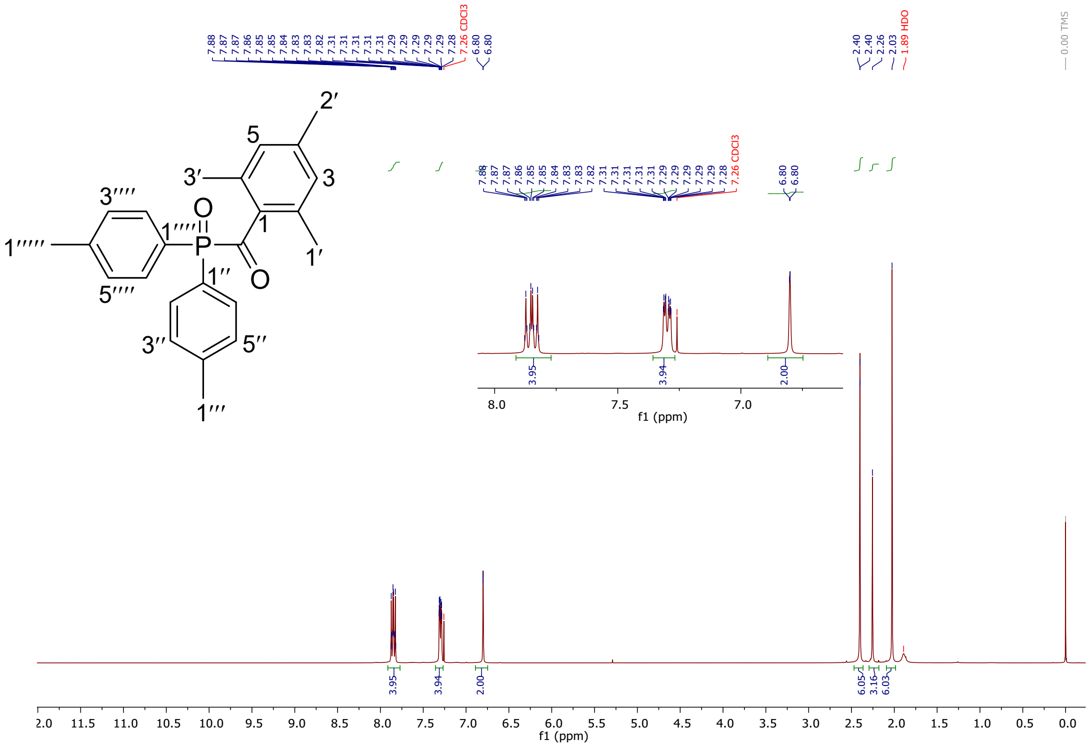

Figure S42. ^{1}H NMR spectrum of (di-*p*-tolylphosphoryl)(mesityl)methanone (**4**) ($CDCl_3$, 400 MHz).

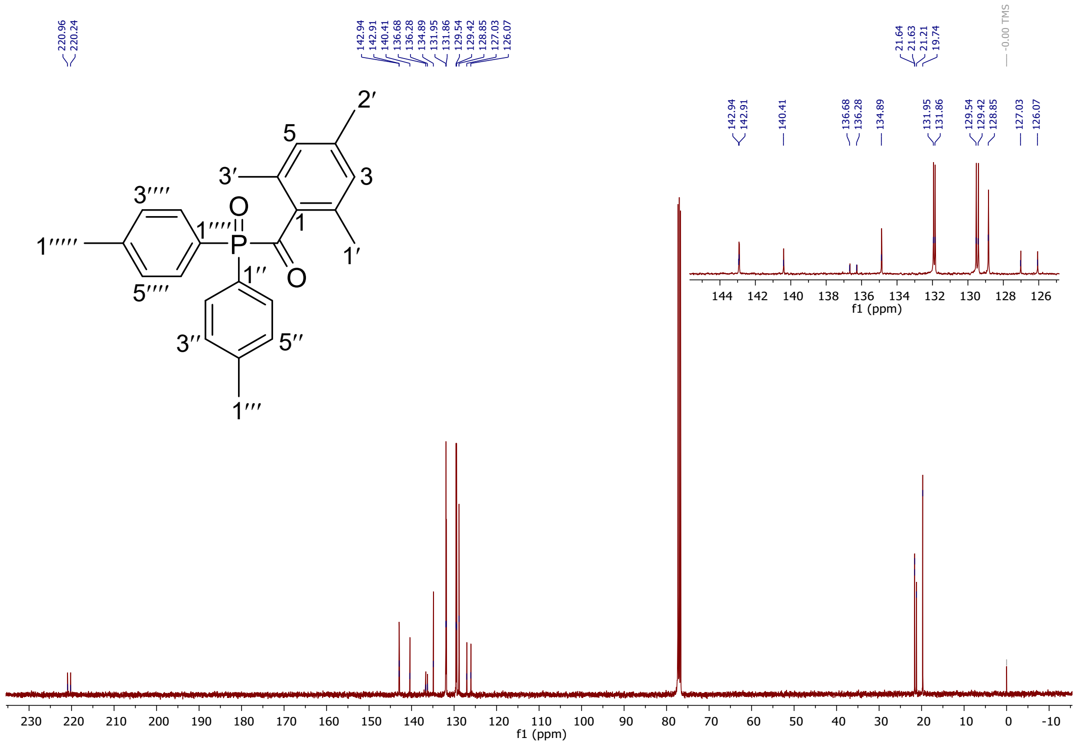

Figure S43. ^{13}C NMR spectrum of (di-*p*-tolylphosphoryl)(mesityl)methanone (**4**) ($CDCl_3$, 101 MHz).

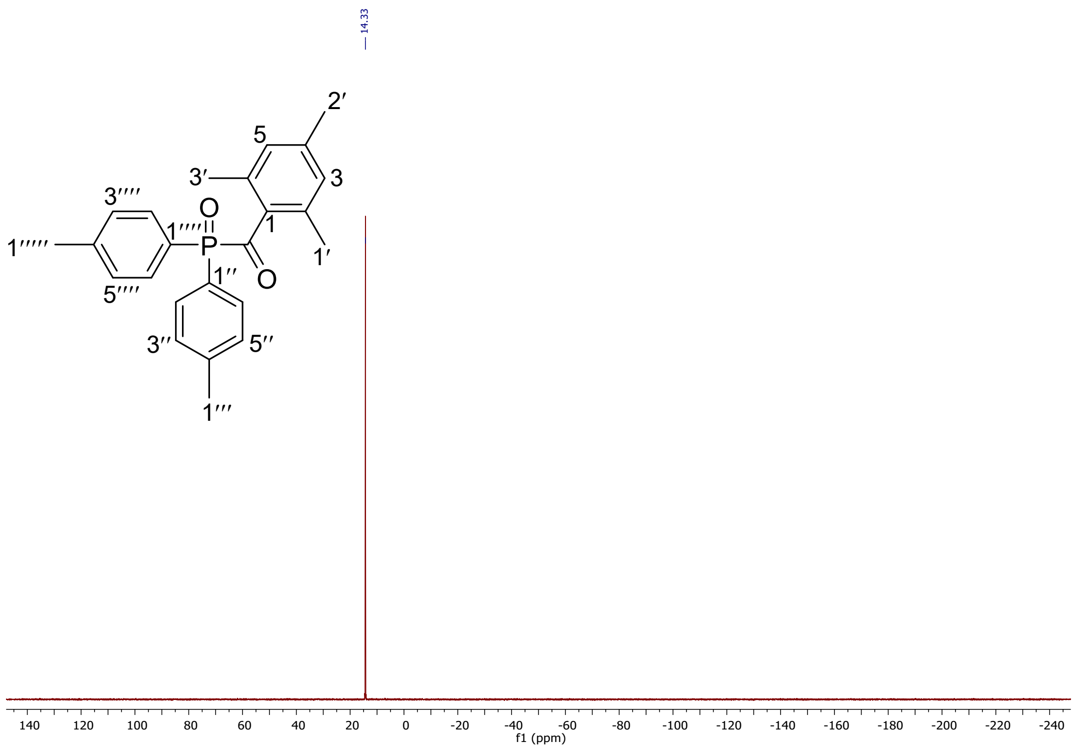

Figure S44. ^{31}P NMR spectrum of (di-*p*-tolylphosphoryl)(mesityl)methanone (**4**) ($CDCl_3$, 162 MHz).

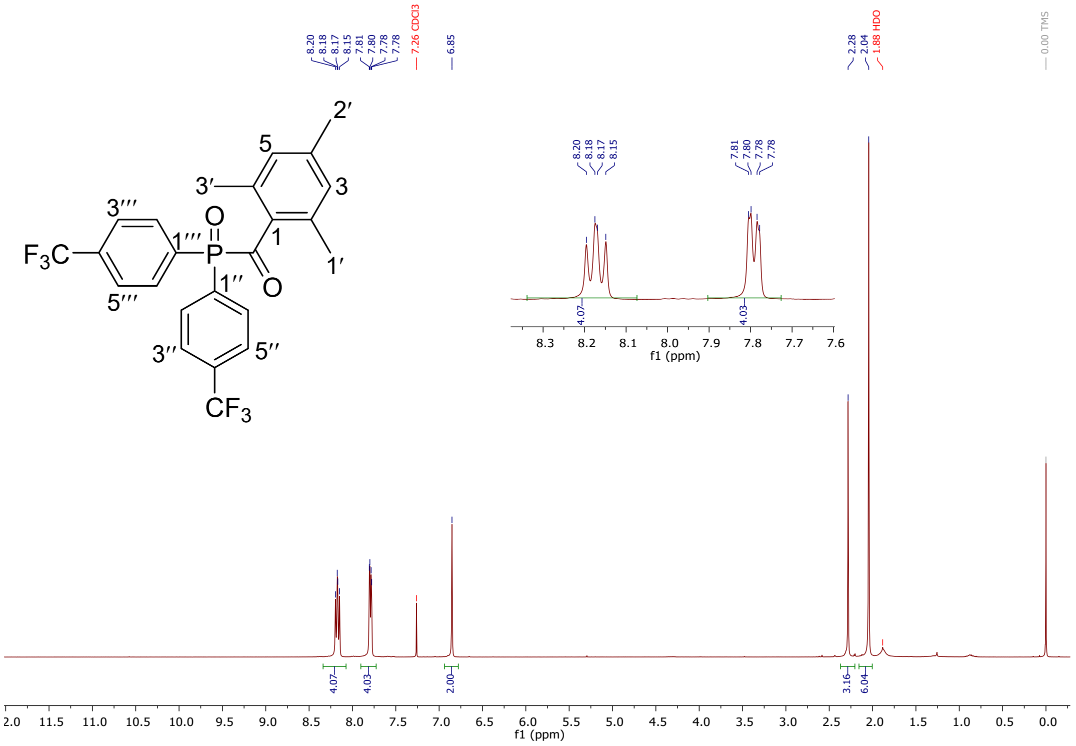

Figure S45. ^{1}H NMR spectrum of (bis(4-(trifluoromethyl)phenyl)phosphoryl)(mesityl)methanone (**5**) ($CDCl_3$, 400 MHz).

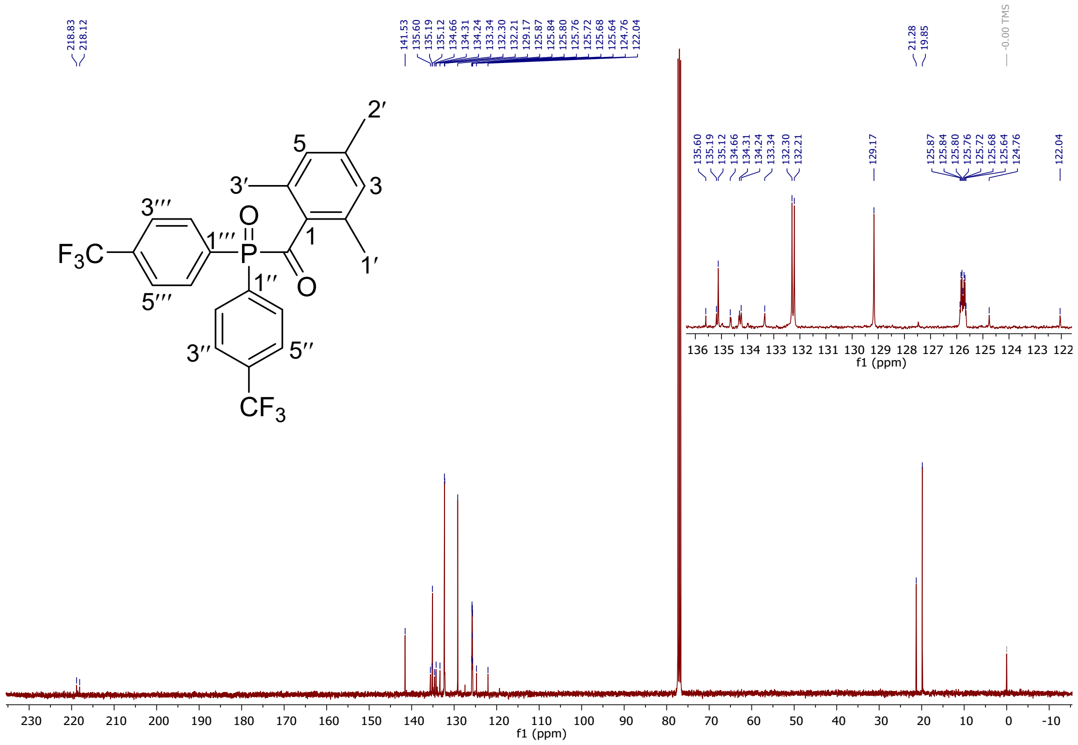

Figure S46. ^{13}C NMR spectrum of (bis(4-(trifluoromethyl)phenyl)phosphoryl)(mesityl)methanone (**5**) ($CDCl_3$, 101 MHz).

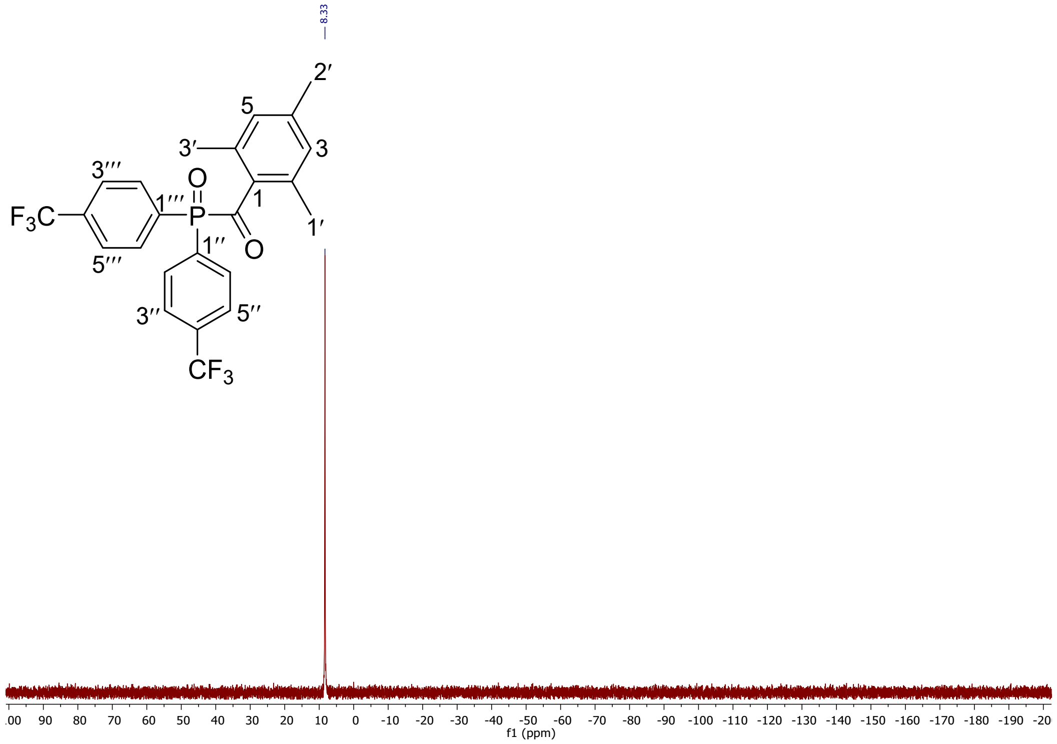

Figure S47. ^{31}P NMR spectrum of (bis(4-(trifluoromethyl)phenyl)phosphoryl)(mesityl)methanone (**5**) ($CDCl_3$, 162 MHz).

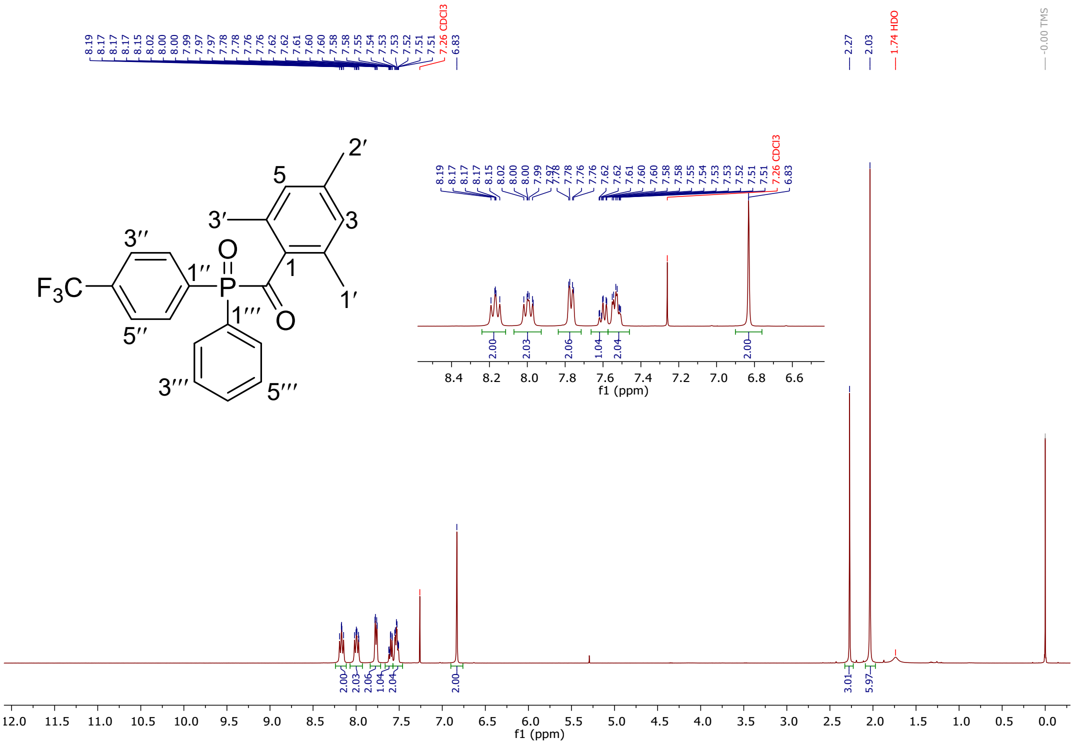

Figure S48. ^{1}H NMR spectrum of mesityl(phenyl(4-(trifluoromethyl)phenyl)phosphoryl)methanone (**6**) ($CDCl_3$, 400 MHz).

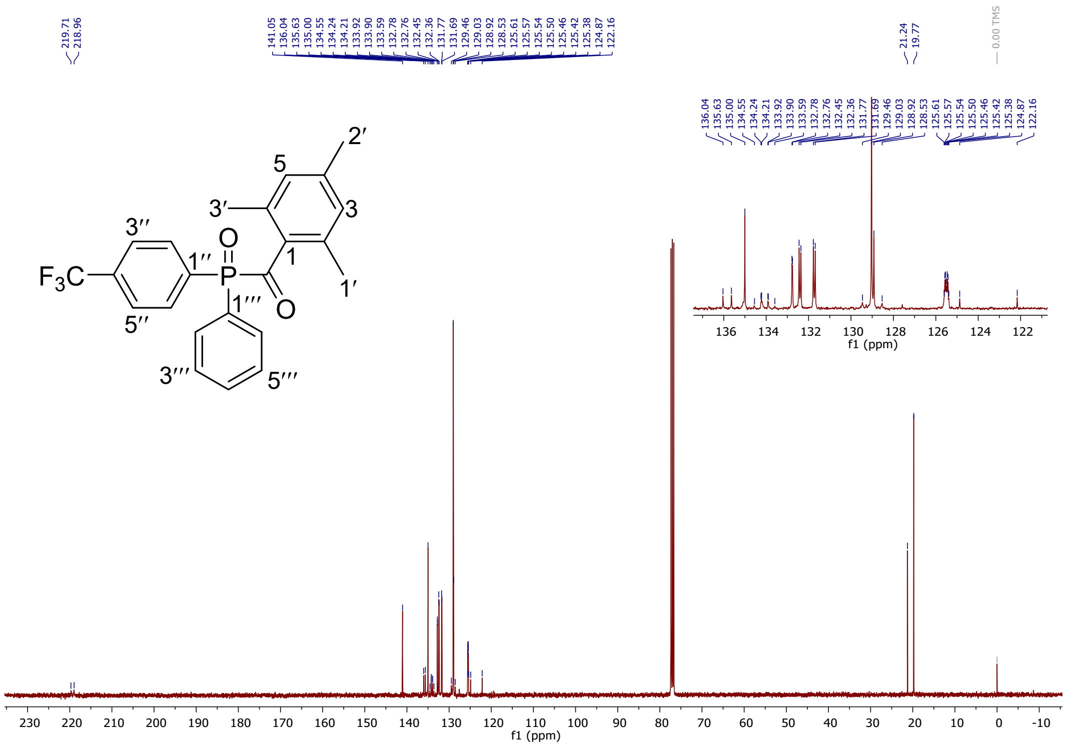

Figure S49. ^{13}C NMR spectrum of mesityl(phenyl(4-(trifluoromethyl)phenyl)phosphoryl)methanone (**6**) ($CDCl_3$, 101 MHz).

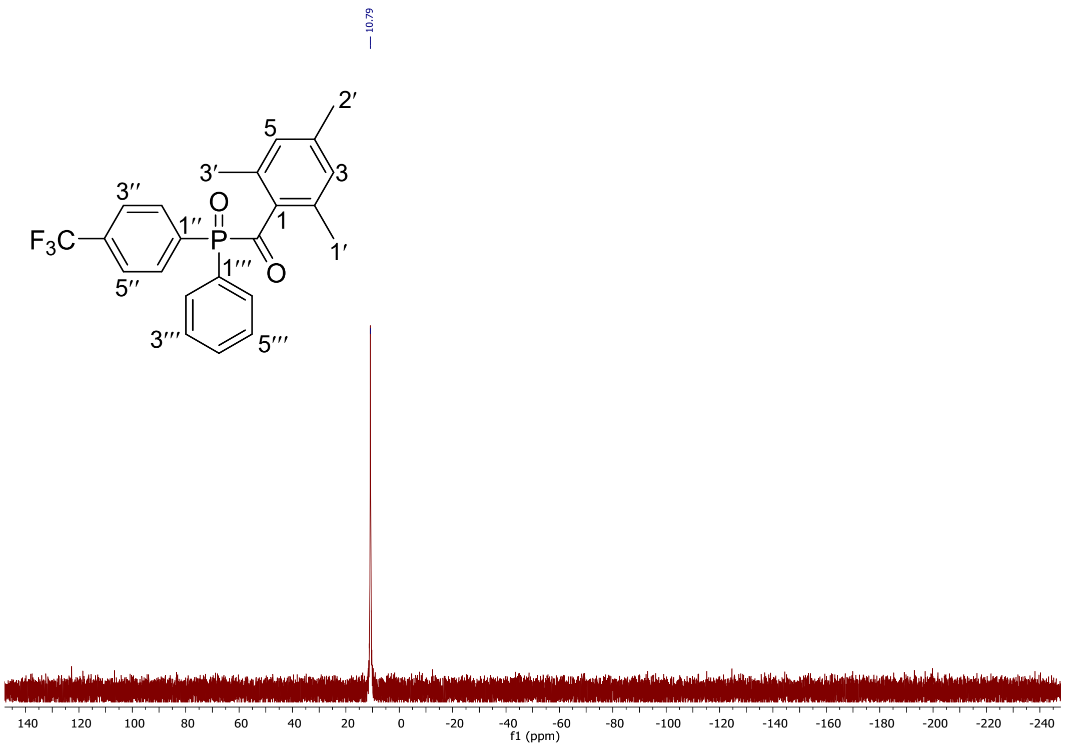

Figure S50. ^{31}P NMR spectrum of mesityl(phenyl(4-(trifluoromethyl)phenyl)phosphoryl)methanone (**6**) ($CDCl_3$, 162 MHz).

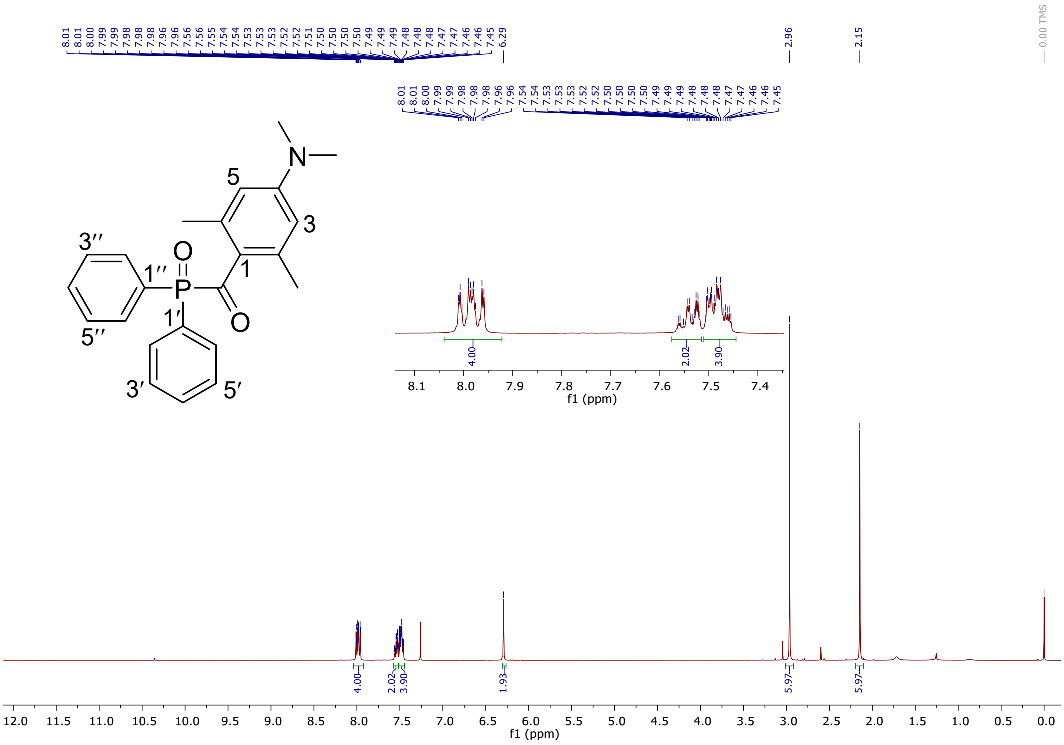

Figure S51. ^{1}H NMR spectrum of (4-(dimethylamino)-2,6-dimethylphenyl)(diphenylphosphoryl)methanone (**7**) ($CDCl_3$, 400 MHz).

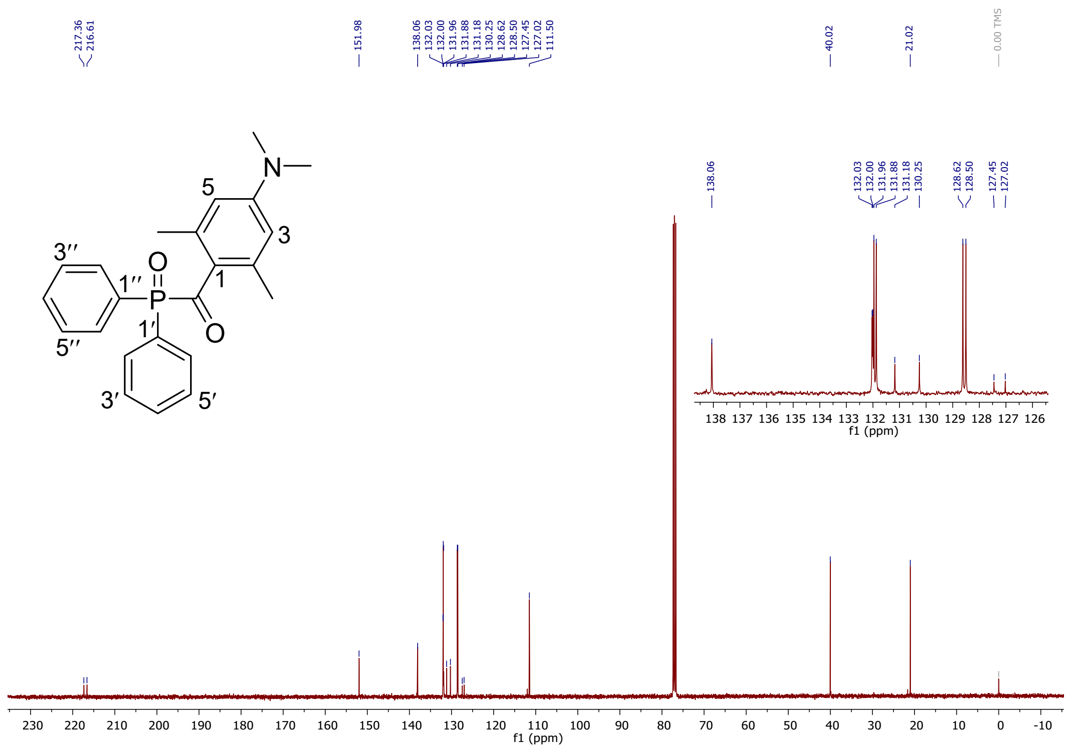

Figure S52. ^{13}C NMR spectrum of (4-(dimethylamino)-2,6-dimethylphenyl)(diphenylphosphoryl)methanone (**7**) ($CDCl_3$, 101 MHz).

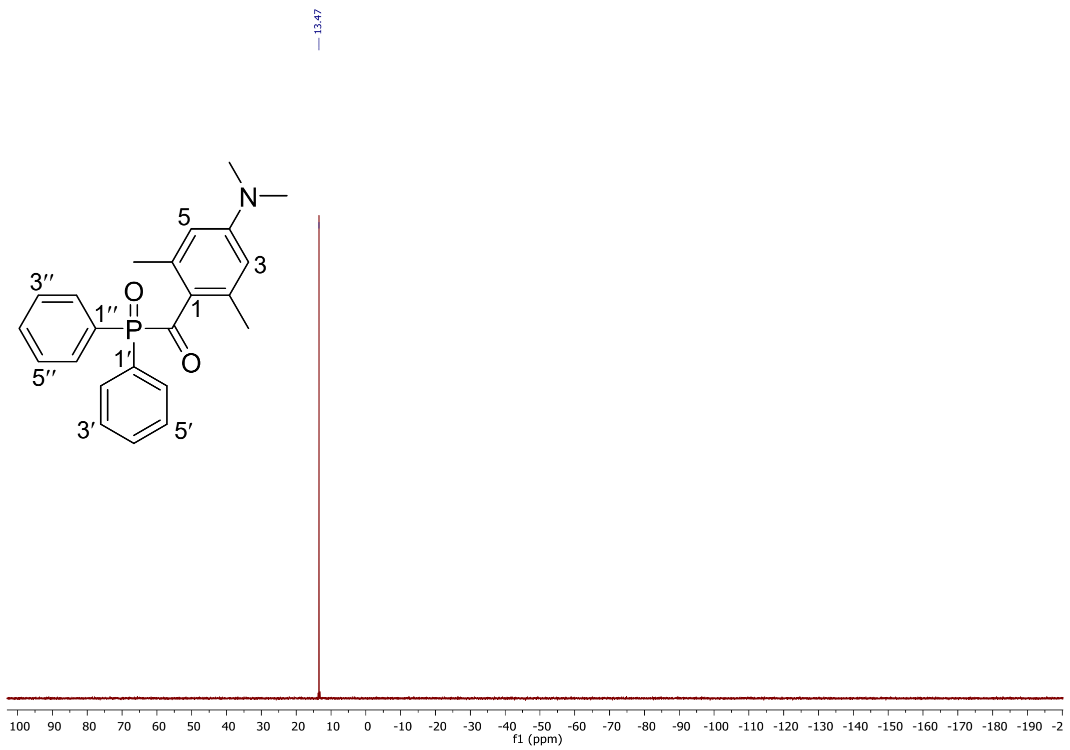

Figure S53. ^{31}P NMR spectrum of (4-(dimethylamino)-2,6-dimethylphenyl)(diphenylphosphoryl)methanone (**7**) ($CDCl_3$, 162 MHz).

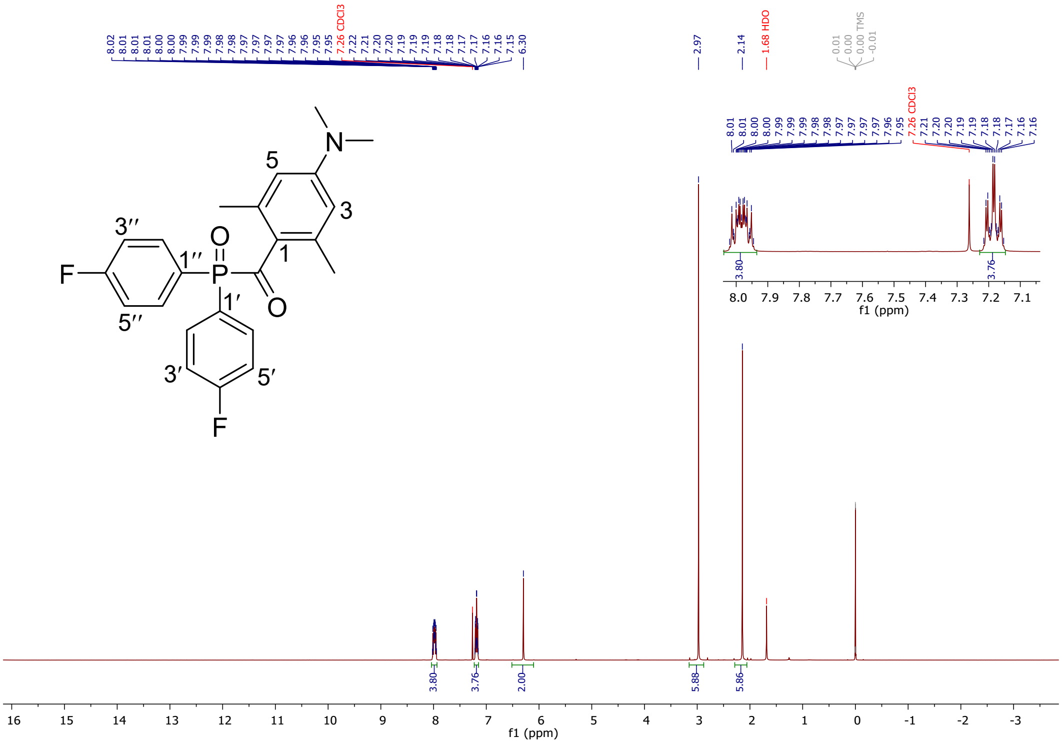

Figure S54. ^{1}H NMR spectrum of (bis(4-fluorophenyl)phosphoryl)(4-(dimethylamino)-2,6-dimethylphenyl)methanone (**8**) ($CDCl_3$, 400 MHz).

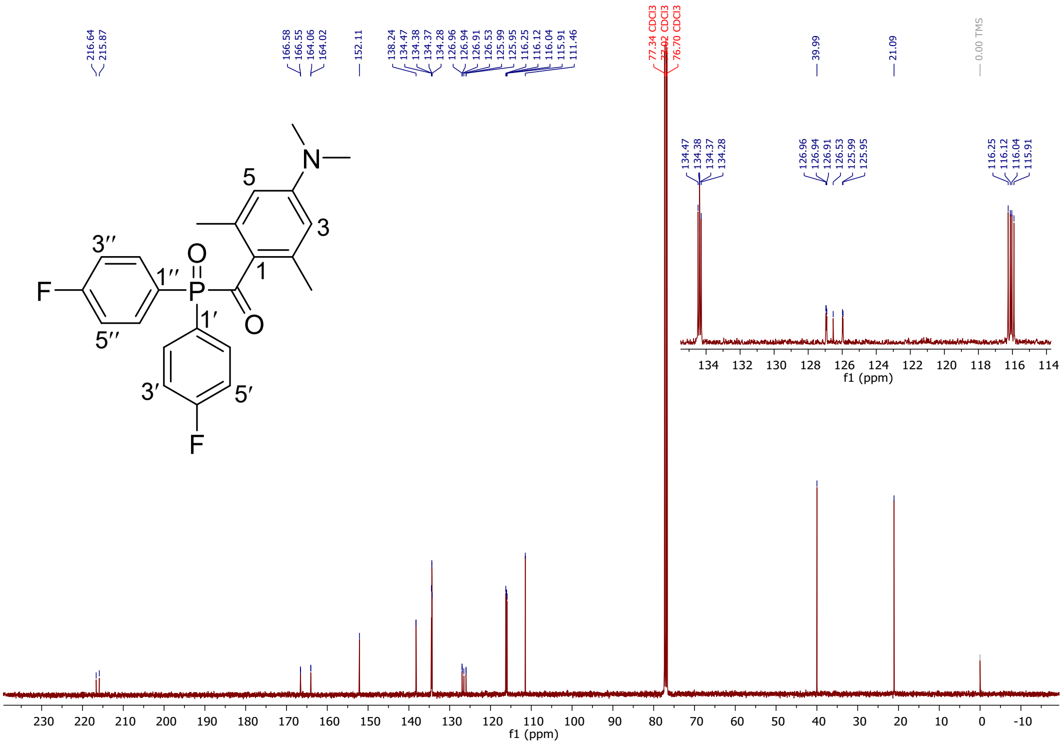

Figure S55. ^{13}C NMR spectrum of (bis(4-fluorophenyl)phosphoryl)(4-(dimethylamino)-2,6-dimethylphenyl)methanone (**8**) ($CDCl_3$, 101 MHz).

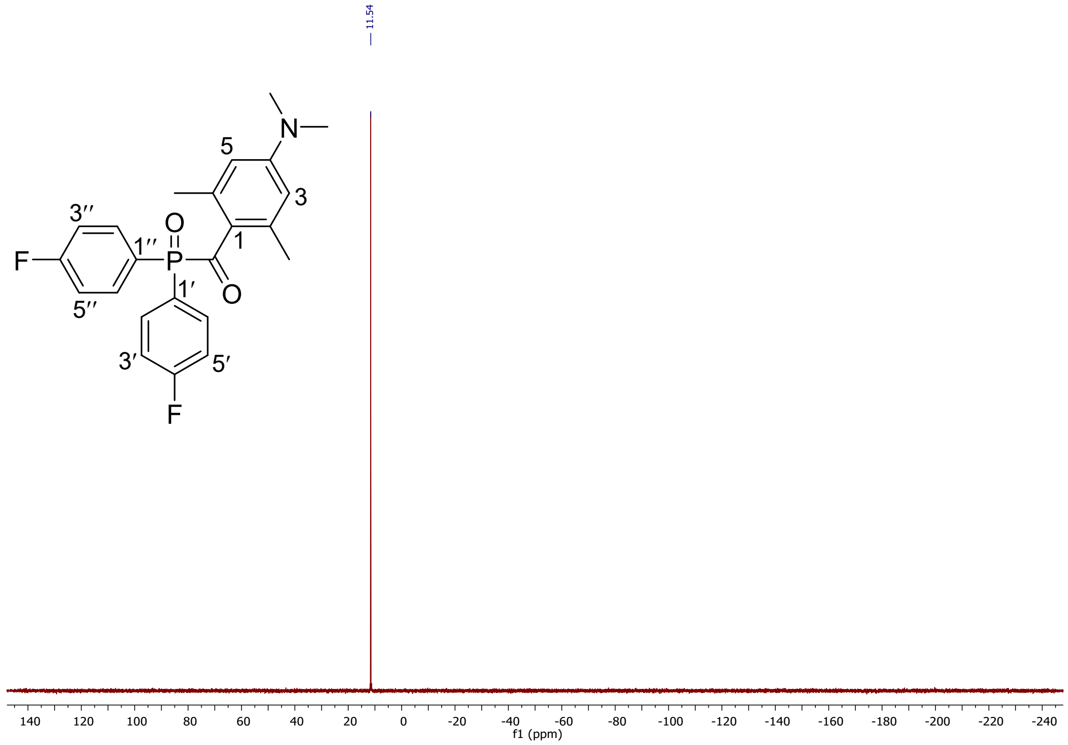

Figure S56. ^{31}P NMR spectrum of (bis(4-fluorophenyl)phosphoryl)(4-(dimethylamino)-2,6-dimethylphenyl)methanone (**8**) ($CDCl_3$, 162 MHz).

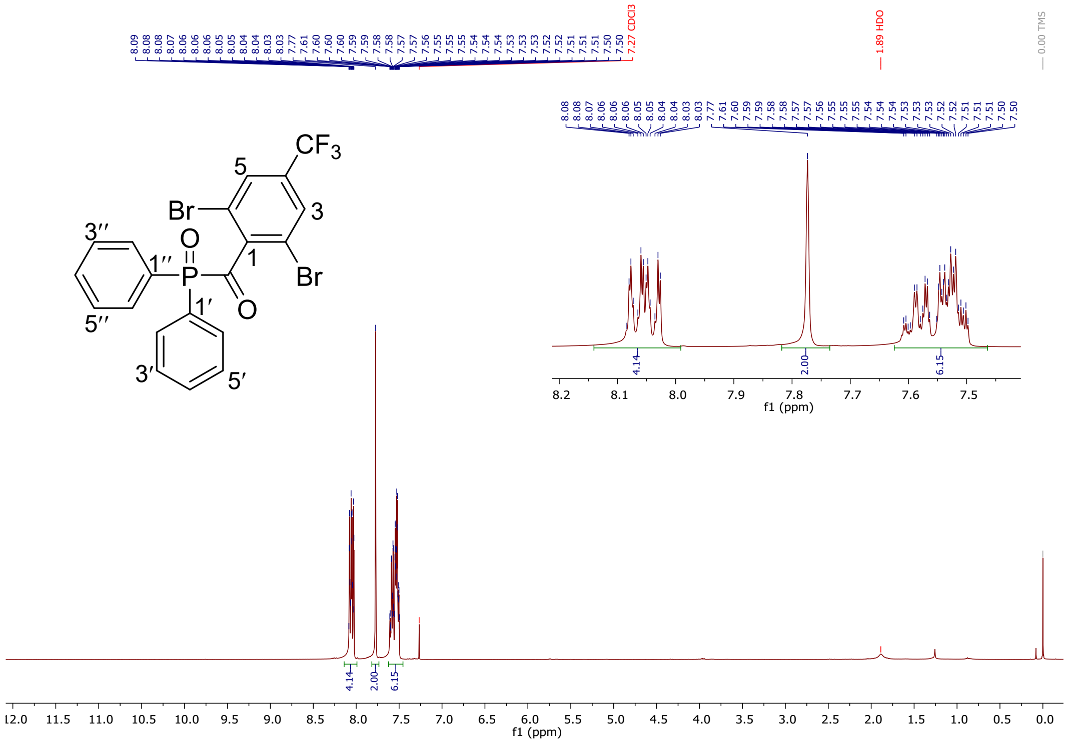

Figure S57. ^{1}H NMR spectrum of (2,6-dibromo-4-(trifluoromethyl)phenyl)(diphenylphosphoryl)methanone (**9**) ($CDCl_3$, 400 MHz).

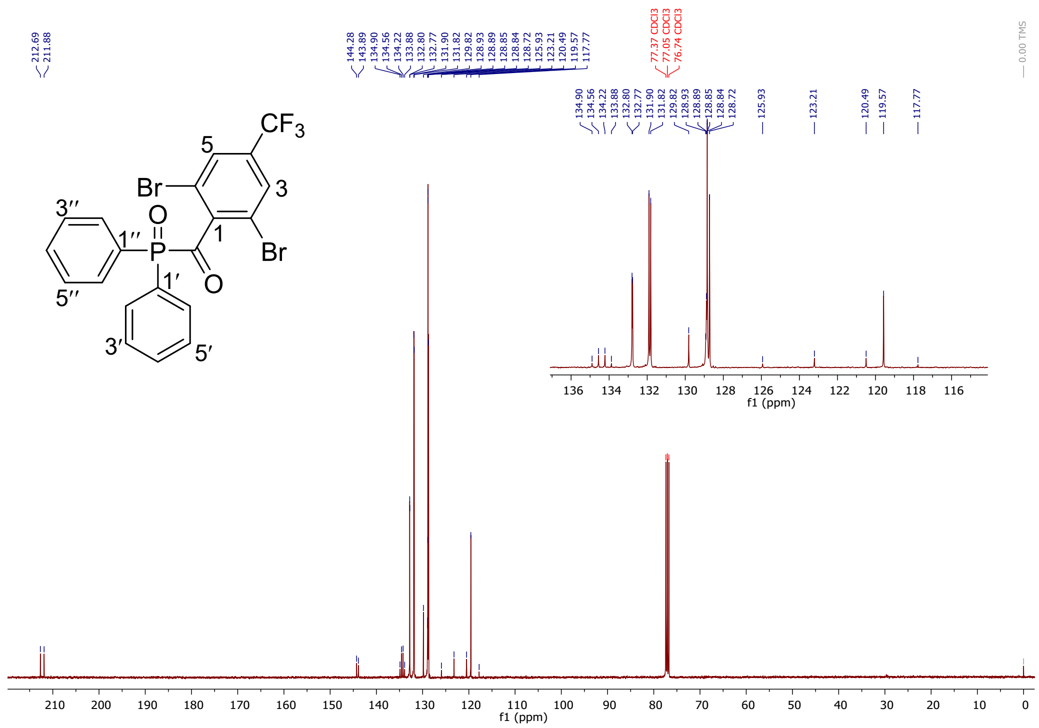

Figure S58. ^{13}C NMR spectrum of (2,6-dibromo-4-(trifluoromethyl)phenyl)(diphenylphosphoryl)methanone (**9**) ($CDCl_3$, 101 MHz).

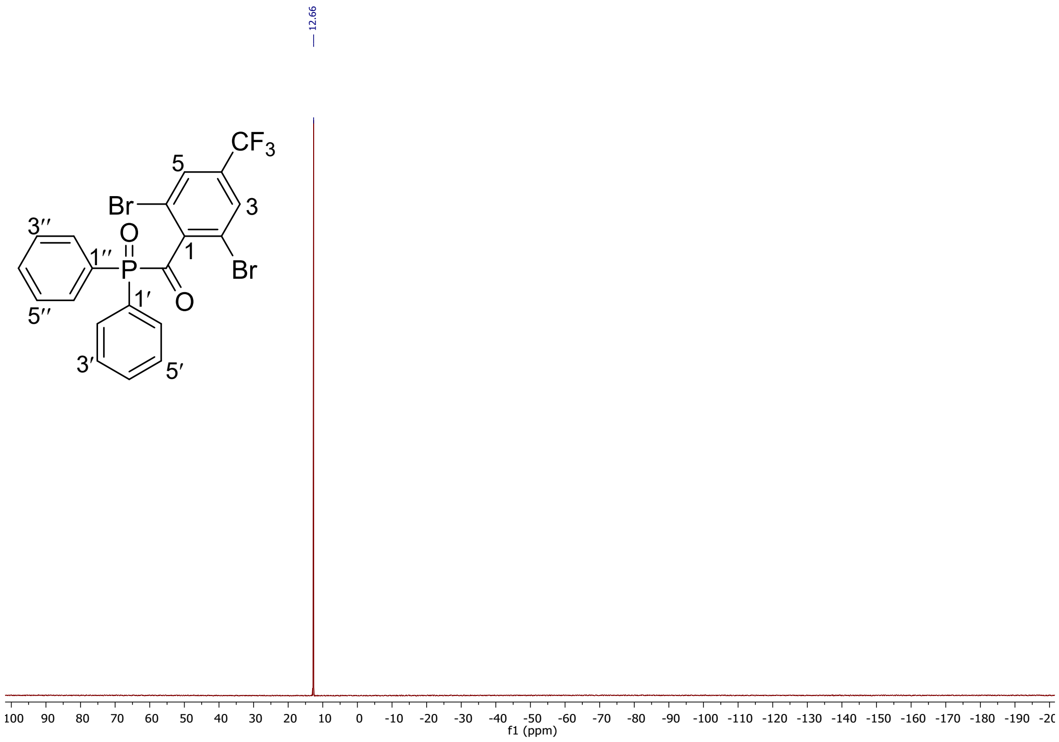

Figure S59. ^{31}P NMR spectrum of (2,6-dibromo-4-(trifluoromethyl)phenyl)(diphenylphosphoryl)methanone (**9**) ($CDCl_3$, 162 MHz).

ESI 3 Mass spectra

ESI 3.1 Mass spectra of all photoinitiators before and after irradiation at 395 nm

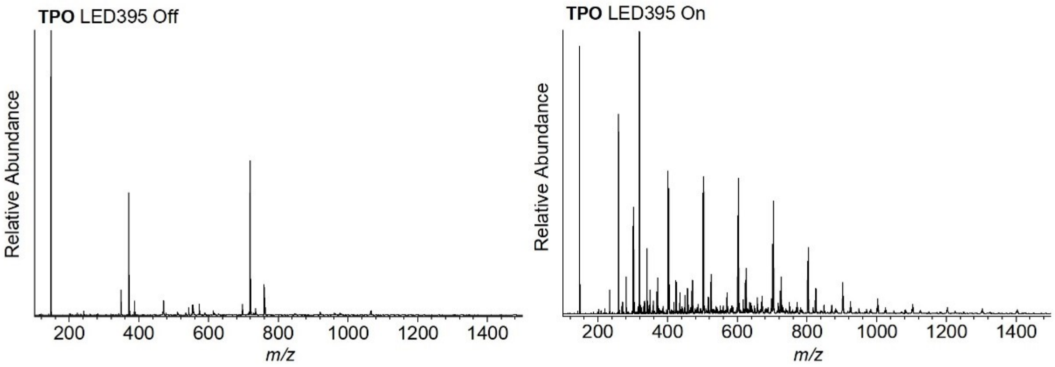

Figure S60. Mass spectra of MMA polymerisation with **TPO** polymerisation (LED395 off (left) and LED395 on (right)).

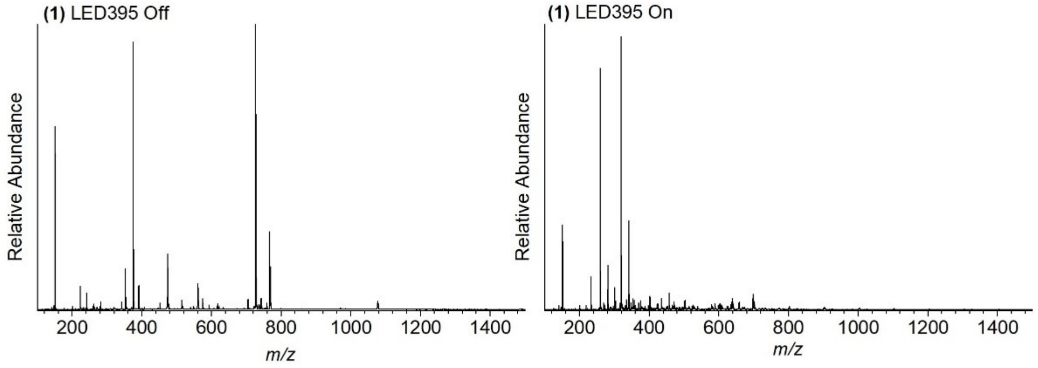

Figure S61. Mass spectra of MMA polymerisation with **1** polymerisation (LED395 off (left) and LED395 on (right)).

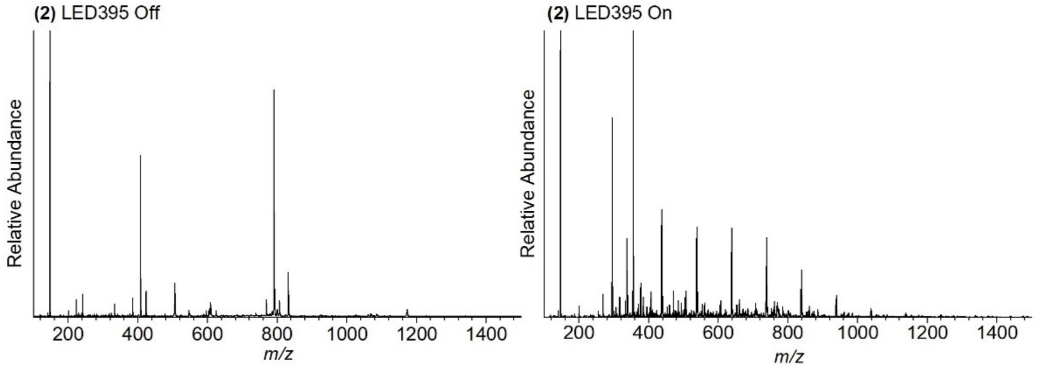

Figure S62. Mass spectra of MMA polymerisation with **2** polymerisation (LED395 off (left) and LED395 on (right)).

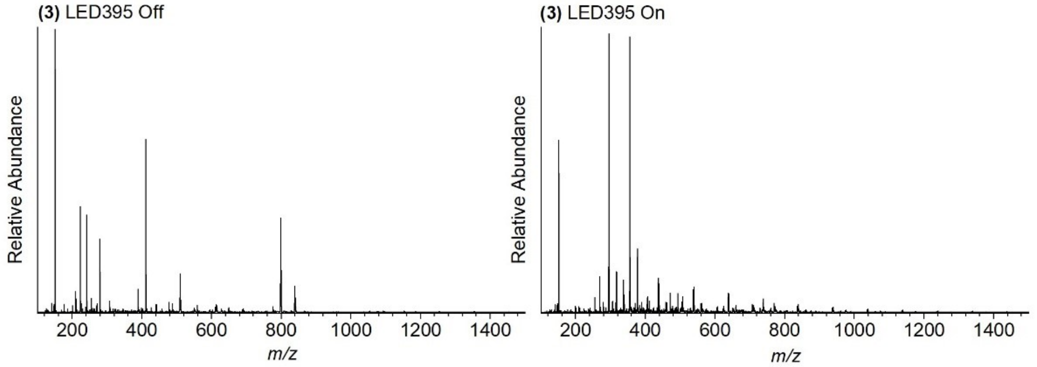

Figure S63. Mass spectra of MMA polymerisation with **3** polymerisation (LED395 off (left) and LED395 on (right)).

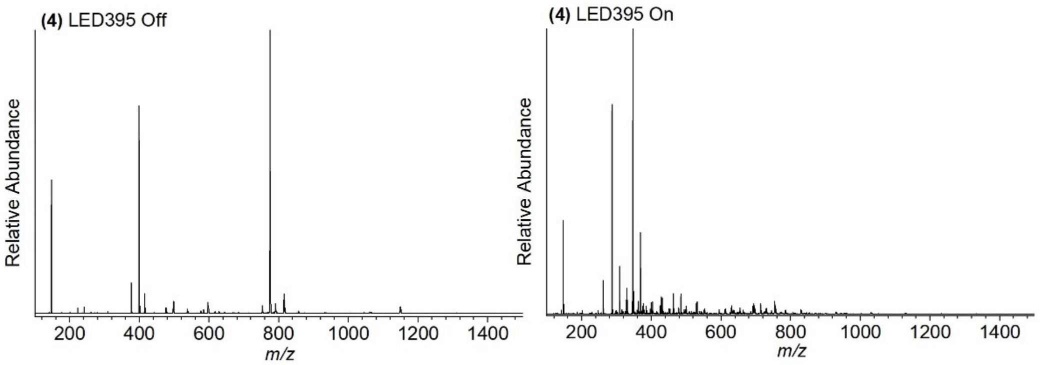

Figure S64. Mass spectra of MMA polymerisation with **4** polymerisation (LED395 off (left) and LED395 on (right)).

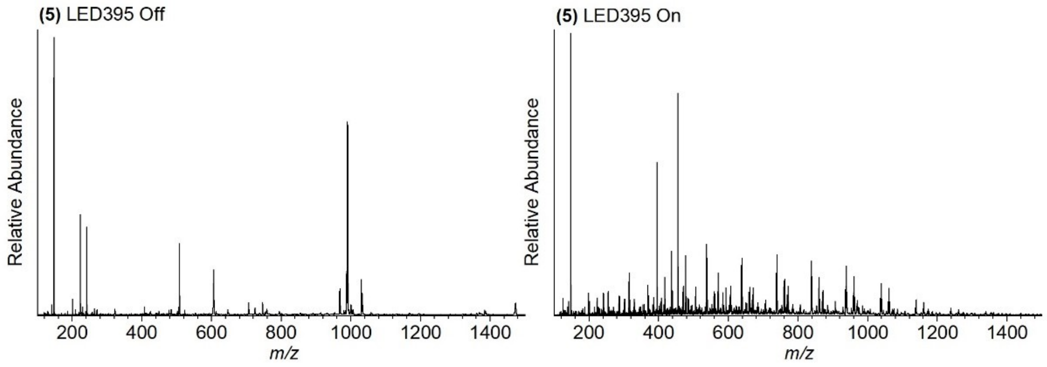

Figure S65. Mass spectra of MMA polymerisation with **5** polymerisation (LED395 off (left) and LED395 on (right)).

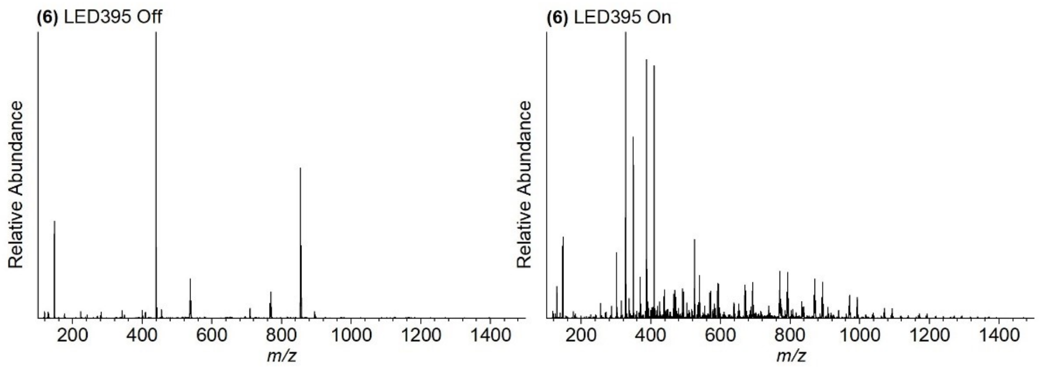

Figure S66. Mass spectra of MMA polymerisation with **6** polymerisation (LED395 off (left) and LED395 on (right)).

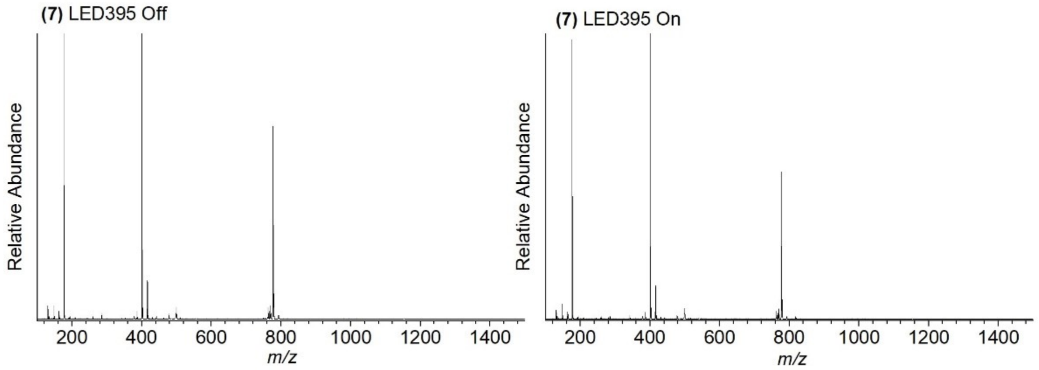

Figure S67. Mass spectra of MMA polymerisation with **7** polymerisation (LED395 off (left) and LED395 on (right)).

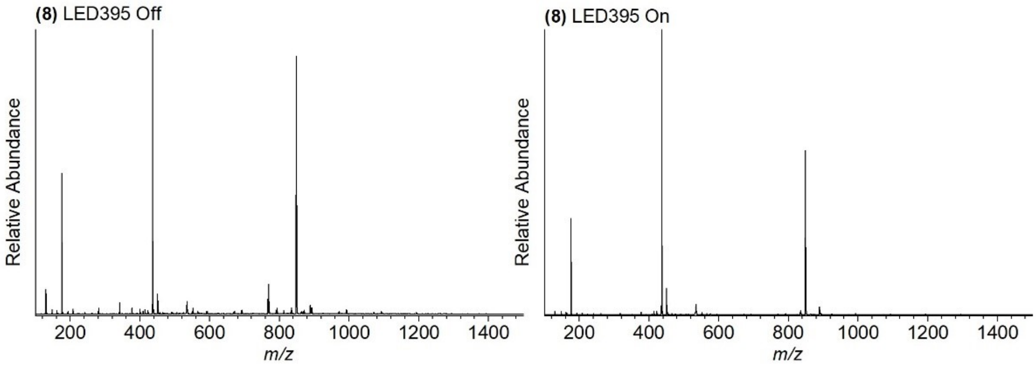

Figure S68. Mass spectra of MMA polymerisation with **8** polymerisation (LED395 off (left) and LED395 on (right)).

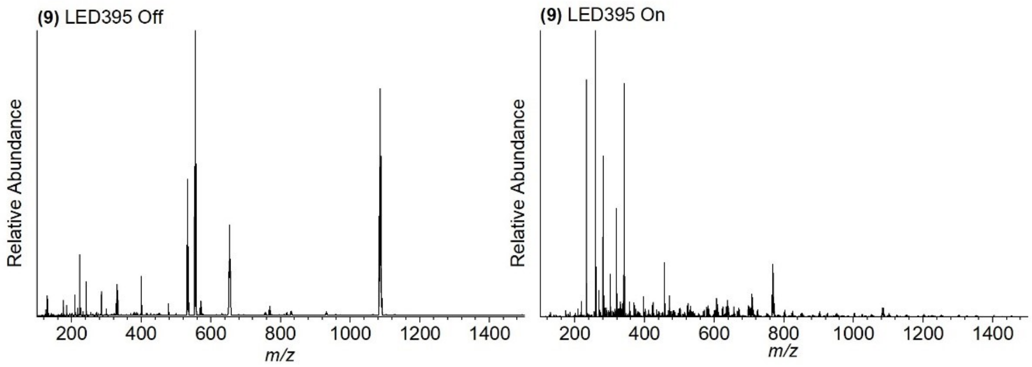

Figure S69. Mass spectra of MMA polymerisation with **9** polymerisation (LED395 off (left) and LED395 on (right)).

ESI 3.2 CID mass spectra

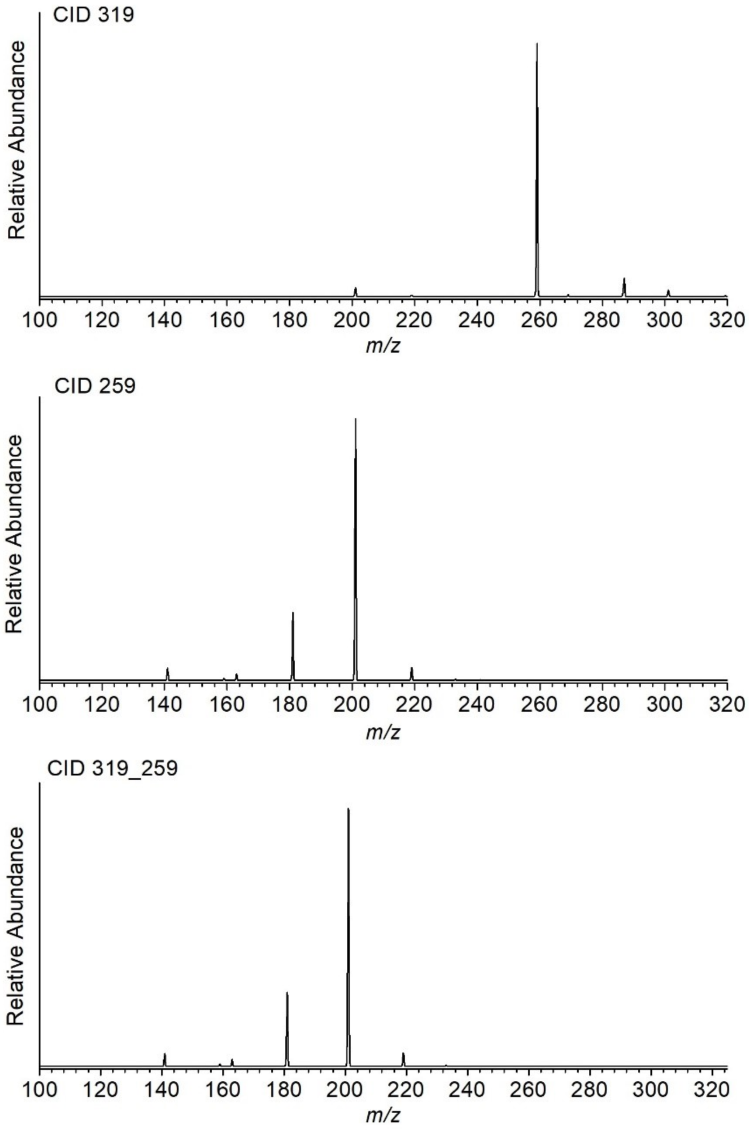

Figure S70. Mass spectra of CID of *m/z* 319 (top), *m/z* 259 (middle), and *m/z* 259 generated by CID of *m/z* 319 (bottom).

ESI 4 References

1. T. Inc., M395L5 - 395 nm, 1130 mW (min) Mounted LED, https://www.thorlabs.com/thorproduct.cfm?partnumber=M395L5, (accessed 23/06, 2025).
2. H.-S. Lin and L. A. Paquette, *Synth. Commun.*, 1994, **24**, 2503-2506.
3. A. M. Borys, *Organometallics*, 2023, **42**, 182-196.
4. R. Nazir, P. Danilevicius, D. Gray, M. Farsari and D. T. Gryko, *Macromol.*, 2013, **46**, 7239-7244.
5. A. K. King, A. Buchard, M. F. Mahon and R. L. Webster, *Chem. Eur. J.*, 2015, **21**, 15960-15963.
6. B. Varga, P. Szemesi, P. Nagy, R. Herbay, T. Holczbauer, E. Fogassy, G. Keglevich and P. Bagi, *J. Org. Chem.*, 2021, **86**, 14493-14507.
7. S. Wiedbrauk, B. Maerz, E. Samoylova, A. Reiner, F. Trommer, P. Mayer, W. Zinth and H. Dube, *J. Am. Chem. Soc.*, 2016, **138**, 12219-12227.
8. E. Zysman-Colman, K. Arias and J. S. Siegel, *Can. J. Chem.*, 2009, **87**, 440-447.
9. C. M. Q. Le, T. Petitory, X. Wu, A. Spangenberg, J. Ortyl, M. Galek, L. Infante, H. Thérien-Aubin and A. Chemtob, *Macromol. Chem. Phys.*, 2021, **222**, 2100217.
10. J. Zheng, X. Feng, Y. Yu, X. Zhen and Y. Zhao, *Phosphorus, Sulfur, and Silicon and the Related Elements*, 2013, **188**, 1080-1087.
11. Y. Sato, S.-i. Kawaguchi and A. Ogawa, *Chem. Commun.*, 2015, **51**, 10385-10388.
12. CN110283206, 2019.
13. J.-Q. Zhang and L.-B. Han, *Org. Lett.*, 2020, **22**, 4633-4637.
